# Supplementary material for: Social and psychological impact of the COVID-19 pandemic on UK medical and nursing students: protocol for a national medical and nursing student survey
Source: BMJ Open. 2022 May 6;12(5):e057467. doi: 10.1136/bmjopen-2021-057467 (PMC9082730; doi:10.1136/bmjopen-2021-057467)
Supplement: Supplementary data [file bmjopen-2021-057467supp001.pdf]

UK N.M.C Approved Adult Nursing Programmes

Approved education institution	Course
**University Of Essex**	Adult Nursing
**University Of Essex**	Adult Nursing (nursing degree apprenticeship route)
**Edinburgh Napier University**	Bachelor of Nursing (Adult)
**University Of Wolverhampton**	Bachelor of Nursing (BNurs (Hons)) Adult Nursing
**University Of Wolverhampton**	Bachelor of Nursing (BNurs (Hons)) Adult Nursing [Apprenticeship]
**University Of Glasgow**	Bachelor of Nursing (Honours)
**University Of Manchester**	Bachelor of Nursing (Hons) (Adult)
**University Of Birmingham**	Bachelor of Nursing (Hons) (Adult)
**University Of Liverpool**	Bachelor of Nursing (Hons) Nursing (Adult)
**University Of Glasgow**	Bachelor of Nursing (ordinary) [fall back award]
**Cardiff University**	Bachelor of Nursing Adult
**University of Derby**	Bachelor of Science (Honours) in Nursing (Adult)
**University of Derby**	Bachelor of Science (Honours) in Nursing (adult) (apprenticeship)
**University Of Sheffield**	BMed Sci (Hons) Nursing (Adult)
**University Of Sheffield**	BMedSci (Hons) Nursing (Adult) Apprenticeship route
**University of Chester**	BN (Hons) Adult Nursing
**University of Chester**	BN (Hons) Adult Nursing
**University of Chester**	BN (Hons) Adult Nursing
**University of Chester**	BN (Hons) Adult Nursing
**University of Chester**	BN (Hons) Adult Nursing

Approved education institution	Course
**University of Chester**	BN (Hons) Adult Nursing Apprenticeship
**University of Chester**	BN (Hons) Adult Nursing Apprenticeship
**University Of Portsmouth**	BN (Hons) Nursing (Adult)
**University of Winchester**	BN (Hons) Nursing (Adult)
**Glyndwr University (formerly North East Wales Inst. of HE)**	BN Nursing
**University Of Edinburgh**	BN nursing (adult)
**University Of Edinburgh**	BN nursing with Honours (adult)
**University of Chester**	BNurs (Hons) Adult
**University of Chester**	BNurs (Hons) Adult (part-time)
**University of Chester**	BNurs (Hons) Adult Apprenticeship
**University Of Wolverhampton**	BNurs (Hons) Adult Nursing
**University Of Wolverhampton**	BNurs (Hons) Adult Nursing Apprenticeship
**University of West London**	BNursing (Hons) Adult
**University Of Hertfordshire**	BSc Hons Nursing (Adult)
**University Of Stirling**	BSc (Honours) Nursing (Adult)
**Coventry University**	BSc (Hons) Adult Nursing
**Coventry University**	BSc (Hons) Adult Nursing (Degree apprenticeship)
**Oxford Brookes University**	BSc (Hons) (Adult)
**London South Bank University**	BSc (Hons) adult nursing
**University of Suffolk**	BSc (Hons) Adult Nursing
**University of West of Scotland**	BSc (Hons) Adult Nursing
**Leeds Beckett University**	BSc (Hons) Adult Nursing
**University of Northampton**	BSc (Hons) Adult Nursing
**Bournemouth University**	BSc (Hons) Adult Nursing

Approved education institution	Course
**University of Roehampton**	BSc (Hons) Adult Nursing
**University Of Dundee**	BSc (Hons) Adult Nursing
**University of West of Scotland**	BSc (Hons) Adult Nursing
**University Of Greenwich**	BSc (Hons) Adult Nursing
**University of Bedfordshire**	BSc (Hons) Adult Nursing
**Bournemouth University**	BSc (Hons) Adult Nursing (Apprenticeship)
**University of Bedfordshire**	BSc (Hons) Adult Nursing (apprenticeship)
**University of Sunderland**	BSc (Hons) Adult Nursing (blended)
**University of Sunderland**	BSc (Hons) Adult Nursing (blended)
**Leeds Beckett University**	BSc (Hons) Adult Nursing (degree apprenticeship)
**Manchester Metropolitan University**	BSc (Hons) Adult Nursing (Pre-registration)
**University of Suffolk**	BSc (Hons) Adult Nursing [degree apprenticeship]
**Coventry University**	BSc (Hons) Adult Nursing Blended Learning
**Coventry University**	BSc (Hons) Adult Nursing Blended Learning Nursing Degree Apprenticeship
**University Of Greenwich**	BSc (Hons) Adult Nursing Degree Apprenticeship
**Solent University**	BSc (Hons) Adult Nursing Practice – apprenticeship
**University of Sunderland**	BSc (Hons) adult nursing practice
**Solent University**	BSc (Hons) Adult Nursing Practice
**University Of Huddersfield**	BSc (Hons) Degree Apprenticeship - Registered Nurse Child (Blended Learning)
**University Of Huddersfield**	BSc (Hons) Degree Apprenticeship-Registered Nurse: Adult

Approved education institution	Course
**BPP**	BSc (Hons) Nursing (Adult) [Nursing Degree Apprenticeship]
**BPP**	BSc (Hons) Nursing (Adult) [pre registration nursing]
**University of East Anglia**	BSc (Hons) nursing - Adult
**University Of Huddersfield**	BSc (Hons) Nursing - Adult
**University Of The West Of England, Bristol**	BSc (Hons) Nursing - Adult
**University of East Anglia**	BSc (Hons) Nursing - Adult Degree Apprenticeship
**Queen Margaret University**	BSc (Hons) Nursing
**University Of Plymouth**	BSc (Hons) Nursing (Adult Health)
**University Of Plymouth**	BSc (Hons) Nursing (Adult Health) - Nurse Degree Apprenticeship
**University Of York**	BSc (hons) Nursing (Adult)
**University of Salford**	BSc (Hons) Nursing (adult)
**University of Bolton**	BSc (Hons) Nursing (adult)
**University of Nottingham**	BSc (Hons) Nursing (adult)
**Buckinghamshire New University**	BSc (Hons) Nursing (Adult)
**Anglia Ruskin University**	BSc (Hons) Nursing (Adult)
**Swansea University**	BSc (Hons) Nursing (Adult)
**Edge Hill University**	BSc (Hons) Nursing (Adult)
**Edge Hill University**	BSc (Hons) Nursing (Adult)
**The Open University**	BSc (Hons) Nursing (Adult)
**The University of Hull**	BSc (Hons) Nursing (Adult)
**The University of Hull**	BSc (Hons) Nursing (Adult)
**University of Ulster**	BSc (Hons) Nursing (Adult)

Approved education institution	Course
**University Of East London**	BSc (Hons) Nursing (Adult)
**University Of Leeds**	BSc (Hons) Nursing (Adult)
**University College Birmingham**	BSc (Hons) Nursing (Adult)
**University Of Southampton**	BSc (Hons) Nursing (Adult)
**Canterbury Christ Church University**	BSc (Hons) Nursing (Adult)
**Birmingham City University**	BSc (Hons) Nursing (Adult)
**Anglia Ruskin University**	BSc (Hons) Nursing (Adult)
**Swansea University**	BSc (Hons) Nursing (Adult)
**The Robert Gordon University**	BSc (Hons) Nursing (Adult)
**University of Bradford**	BSc (Hons) Nursing (Adult)
**The Open University**	BSc (Hons) Nursing (Adult)
**The Open University**	BSc (Hons) Nursing (Adult)
**University of Gloucestershire**	BSc (Hons) Nursing (Adult)
**University Of Hertfordshire**	BSc (Hons) Nursing (adult) - Nursing Degree Apprenticeship
**The Open University**	BSc (Hons) Nursing (Adult) - Nursing Degree Apprenticeship
**Birmingham City University**	BSc (Hons) Nursing (Adult) - Nursing Degree Apprenticeship
**University of Gloucestershire**	BSc (Hons) Nursing (Adult) (Apprenticeship)
**University of Gloucestershire**	BSc (Hons) Nursing (Adult) (Blended Learning)
**Edge Hill University**	BSc (Hons) Nursing (Adult) (fall back award)
**University Of Leeds**	BSc (Hons) Nursing (Adult) (International)
**Canterbury Christ Church University**	BSc (Hons) Nursing (Adult) (NDA)
**University of Bolton**	BSc (Hons) Nursing (Adult) (Part-time)

Approved education institution	Course
**University of Bolton**	BSc (Hons) Nursing (Adult) (Registered Nursing Higher Apprenticeship) (Part-time)
**University of Bradford**	BSc (Hons) Nursing (Adult) [fall back award]
**Buckinghamshire New University**	BSc (Hons) Nursing (Adult) Apprenticeship
**University of Salford**	BSc (Hons) Nursing (adult) Nurse Degree Apprentice
**University of Bolton**	BSc (Hons) Nursing (adult) nurse degree apprenticeship (NDA) route
**Middlesex University**	BSc (Hons) Nursing (adult) seconded pathway
**De Montfort University**	BSc (Hons) Nursing (Adult) with NMC Registration (Apprenticeship route)
**University Of East London**	BSc (Hons) Nursing (Adult)-Registered Nurse (Degree) Apprenticeship Route
**Queen Margaret University**	BSc (Hons) Nursing (fall back award)
**University of Lincoln**	BSc (Hons) Nursing (Registered Nurse - Adult)
**University of South Wales**	BSc (Hons) Nursing Adult
**Nottingham Trent University**	BSc (Hons) Nursing Adult
**University Of Brighton**	BSc (Hons) Nursing Adult
**University of Worcester**	BSc (Hons) Nursing Adult
**Sheffield Hallam University**	BSc (Hons) Nursing Adult (Part Time)
**University Of The West Of England, Bristol**	BSc (Hons) Nursing apprenticeship - Adult
**University Of Huddersfield**	BSc (Hons) Nursing Child (Blended Learning)
**University of Sunderland**	BSc (Hons) nursing degree apprenticeship (adult)
**Nottingham Trent University**	BSc (Hons) Nursing Degree Apprenticeship Adult
**Swansea University**	BSc (Hons) Nursing part-time (Adult)

Approved education institution	Course
**University Of Northumbria At Newcastle**	BSc (Hons) nursing science (adult)
**University Of Northumbria At Newcastle**	BSc (Hons) nursing science apprenticeship (adult)
**Teesside University**	BSc (Hons) Nursing Studies (Adult) (Pre-registration) Apprenticeship route
**Glasgow Caledonian University**	BSc (Hons) Nursing Studies: Adult Nursing
**Liverpool John Moores University**	BSc (Hons) Nursing with Registered Nurse Status (Adult)
**Liverpool John Moores University**	BSc (Hons) Nursing with Registered Nurse Status (Adult)
**University of Cumbria**	BSc (Hons) Nursing/RN:Adult Nursing
**University of Cumbria**	BSc (Hons) Nursing/RN:Adult Nursing (Apprenticeship)
**Keele University**	BSc (Hons) Nursing: Adult
**Keele University**	BSc (Hons) Nursing: Adult (NDA)
**Kingston University & St George's University of London**	BSc Adult Nursing
**University Of Dundee**	BSc Adult Nursing
**University of West of Scotland**	BSc Adult Nursing
**University Of Greenwich**	BSc Adult Nursing (Degree Apprenticeship) (fall back award)
**University Of Greenwich**	BSc Adult Nursing (fall back award)
**Middlesex University**	BSc Hons Nursing Adult
**University of Highlands and Islands**	BSc Nursing
**City, University of London**	BSc Nursing (Adult)
**Oxford Brookes University**	BSc Nursing (Adult)
**The Robert Gordon University**	BSc Nursing (Adult)

Approved education institution	Course
**The Robert Gordon University**	BSc Nursing (Adult)
**University Of Stirling**	BSc Nursing (Adult)
**The Robert Gordon University**	BSc Nursing (Adult) (fall back award)
**Glasgow Caledonian University**	BSc Nursing Studies: Adult Nursing
**King's College London**	BSc Nursing with Registration as an Adult Nurse
**University of Ulster**	BSc(Hons) Nursing (Adult)
**University of Lincoln**	Degree Science Nursing (Registered Nurse - Adult: Apprenticeship Route)
**University of Salford**	MA Nursing (adult)
**University Of Wolverhampton**	Master of Adult Nursing
**University Of Wolverhampton**	Master of Nursing (Adult Nursing)
**University Of Birmingham**	Master of Nursing (Adult)
**University Of Wolverhampton**	Master of Nursing (MNurs) Adult Nursing
**University Of Birmingham**	Master of Science in Nursing (Adult)
**University of Derby**	Master of Science in Nursing (Adult)
**University of Derby**	Master of Science in Nursing (adult) (apprenticeship)
**University Of Manchester**	Masters in Nursing (Adult)
**University Of Edinburgh**	Masters in Nursing with pre-registration (adult)
**University Of Sheffield**	MMedSci Nursing (Adult)
**Queen Margaret University**	MN Nursing
**Edge Hill University**	MNSW Adult Nursing and Social Work
**University of Sunderland**	MNurse (Adult)
**University Of York**	MNursing (Adult)
**University Of Hertfordshire**	MSc Hons Nursing (Adult)

Approved education institution	Course
**University Of Northumbria At Newcastle**	MSc (Hons) nursing science (adult)
**University Of Dundee**	MSc Adult Nursing
**University Of Greenwich**	MSc Adult Nursing
**University of Chester**	MSc Adult Nursing
**University of Chester**	MSc Adult Nursing
**University of Chester**	MSc Adult Nursing
**University of Bedfordshire**	MSc Adult Nursing
**London South Bank University**	MSc adult nursing
**University of West of Scotland**	MSc Adult Nursing
**University of Chester**	MSc Adult Nursing
**Bournemouth University**	MSc Adult Nursing
**Kingston University & St George's University of London**	MSc Adult Nursing
**Coventry University**	MSc Adult Nursing
**University of Bedfordshire**	MSc Adult Nursing (apprenticeship)
**Birmingham City University**	MSc Adult Nursing (pre-registration)
**Manchester Metropolitan University**	MSc Adult Nursing (Pre-registration)
**University of Chester**	MSc Adult Nursing Apprenticeship
**Coventry University**	MSc Adult Nursing Blended Learning (pre-registration)
**Coventry University**	MSc Adult Nursing Blended Learning (pre-registration) Nursing Degree Apprenticeship
**University Of Southampton**	MSc Nursing (Adult)
**Canterbury Christ Church University**	MSc Nursing (Adult)
**City, University of London**	MSc Nursing (Adult)

Approved education institution	Course
**University Of Hertfordshire**	MSc Nursing (adult) - Nursing Degree Apprenticeship (part time route)
**University of Nottingham**	MSc nursing (graduate entry) (adult)
**University of Lincoln**	MSc Nursing (Pre-Registration – Adult)
**Edge Hill University**	MSc Nursing (Pre-registration Adult)
**University Of Huddersfield**	MSc Nursing (pre-registration) - Adult
**Swansea University**	MSc Nursing Pre-registration (Adult)
**Teesside University**	MSc Nursing Studies (Adult Apprenticeship)
**Glasgow Caledonian University**	MSc Nursing Studies: Adult Nursing
**University of Central Lancashire**	MSc nursing with registered nurse (adult)
**Liverpool John Moores University**	MSc Nursing with Registered Nurse Status (Adult)
**Liverpool John Moores University**	MSc Nursing with Registered Nurse Status (Adult)
**King's College London**	MSc Nursing with Registration as an Adult Nurse
**University Of Plymouth**	MSc Pre-registration Nursing (Adult Health)
**University Of Huddersfield**	MSc Pre-registration Nursing apprenticeship-Adult
**University Of Huddersfield**	MSc Pre-registration Nursing Apprenticeship Child (Blended Learning)
**University Of Huddersfield**	MSc Pre-registration Nursing Child (Blended Learning
**University of Cumbria**	MSc Pre-Registration Nursing/Registered Nurse: Adult Nursing
**Edge Hill University**	MSci Nurse Paramedic (Adult)
**University Of Exeter**	MSci nursing (adult)
**University of Leicester**	MSci Nursing with Leadership Adult Nursing

Approved education institution	Course
**Middlesex University**	Nurse Degree Apprenticeship Adult (4 year direct entry route)
**Middlesex University**	Nurse Degree Apprenticeship Adult (4 year part-time employed route)
**University of West London**	Nurse Degree Apprenticeship BNursing (Hons) Adult
**University of Central Lancashire**	Nursing Degree Apprenticeship route – Adult
**Teesside University**	Nursing Studies (Adult) (Pre-registration) BSc (Hons)
**Teesside University**	Nursing Studies (Adult) (Pre-Registration) MSc
**University of West of Scotland**	PG Dip Adult Nursing
**London South Bank University**	PG Dip adult nursing
**University Of Greenwich**	PG Dip Adult Nursing (fall back award)
**University of South Wales**	PG Dip Nursing Adult
**University of West London**	PG Diploma Nursing Adult
**Edge Hill University**	PgDip Nursing (second registration Adult)
**University Of Southampton**	Postgraduate Diploma Nursing (Adult)
**The Queen's University Of Belfast**	Pre-registration nursing - Adult
**Sheffield Hallam University**	Pre-registration nursing - Adult
**The Queen's University Of Belfast**	Pre-registration nursing - Adult (Graduate Entry)
**Staffordshire University**	Registered Nurse (Adult)
**University Of Brighton**	Registered Nurse Degree Apprenticeship (Adult)

UK M.S.C Approved Medicine and Surgery Programmes

Institution Name
University of Aberdeen School of Medicine and Dentistry
Anglia Ruskin University School of Medicine
Aston University Medical School
Barts and The London School of Medicine and Dentistry
University of Birmingham College of Medical and Dental Sciences
Brighton and Sussex Medical School
University of Bristol Medical School
University of Buckingham Medical School
University of Cambridge School of Clinical Medicine
Cardiff University School of Medicine
University of Dundee School of Medicine
Edge Hill University Medical School
The University of Edinburgh Medical School
University of Exeter Medical School
University of Glasgow School of Medicine
Hull York Medical School
Imperial College London Faculty of Medicine
Keele University School of Medicine
Kent and Medway Medical School
King's College London GKT School of Medical Education
Lancaster University Medical School
University of Leeds School of Medicine
University of Leicester Medical School
University of Liverpool School of Medicine
University of Manchester Medical School
Newcastle University School of Medical Education
Norwich Medical School
University of Nottingham School of Medicine

University of Nottingham - Lincoln Medical School
University of Oxford Medical Sciences Division
Plymouth University Peninsula Schools of Medicine and Dentistry
Queen's University Belfast School of Medicine
University of Sheffield Medical School
University of Southampton School of Medicine
University of St Andrews School of Medicine
St George's, University of London
University of Sunderland School of Medicine
Swansea University Medical School
University of Central Lancashire School of Medicine
University College London Medical School
University of Warwick Medical School
