# Supplementary material for: Social and psychological impact of the COVID-19 pandemic on UK medical and nursing students: protocol for a national medical and nursing student survey
Source: BMJ Open. 2022 May 6;12(5):e057467. doi: 10.1136/bmjopen-2021-057467 (PMC9082730; doi:10.1136/bmjopen-2021-057467)
Supplement: Supplementary data [file bmjopen-2021-057467supp002.pdf]

Patient Information Section

General information

The aim of this study is to determine the impact of the COVID-19 pandemic on the mental health of UK medical and nursing students, one year down the line from the initial outbreak. We are inviting all medical and nursing students enrolled at university in the UK to participate in this study. Please read the following section carefully before taking part. If you have any questions regarding the study please contact the research team via the following email: kate.saunders@psych.ox.ac.uk

What is the purpose of the study?

The purpose of SPICE-20 is to evaluate the continued impact of the COVID-19 pandemic on UK medical students, one year on from the SPICE-19 study. In addition, we aim to determine the effect on UK nursing students. In doing so we hope to provide feedback to UK medical schools and Nursing institutions about the current collective mental health of student healthcare professionals. We believe this information is vital to ensure adequate mental wellbeing service provision is in place at higher education institutions.

Why am I being invited to participate in this study?

You have been invited to take part in this study because we are recruiting medical and nursing students to participate and feedback on their continued mental wellbeing during the UK pandemic. You are eligible to participate if you are a current UK medical or nursing student in the UK and have access to the internet.

What happens if I choose to participate?

Participation in this study is entirely voluntary. To be involved you will be required to complete a short survey online. The survey will ask for general background details about you including: your current educational status, age, gender, ethnicity. Additionally, questions will ask about your current mood and mental wellbeing. Your decision to participate in this study will not have any academic impact and will be entirely anonymous. The survey is expected to take approximately 10-15 minutes to complete however it may be done as quick or slow as you like. There is no prior knowledge required to participate. We will ask for your consent for collection and storage of data in accordance with General

Data Protection Regulation (GDPR) before the survey begins. More information regarding the rules and regulations of GDPR can be found at www.gdpr-info.eu .

Do I have to participate?

As stated above, participation in the SPICE-20 study is entirely voluntary and anonymous. Consent for involvement may be withdrawn anytime prior to completion of the survey. To do so, simply close the tab or browser containing the survey. Following this, no personal data provided up to that point in the survey will be stored or used for research or any other purpose. Completion of all questions is entirely optional. This means that participation is still possible even if there are certain questions you would prefer not to answer. If there is a question you would like to omit from your responses, simply select the option "Prefer not to answer". Any data collected will not be provided to your higher education institution without being anonymised, meaning your feedback will not make your identifiable.

Are there any risks of being involved?

Some of the questions included in the questionnaire may potentially cause some distress by brining up painful memories. If at any point during completion of the survey you are feeling distressed you may stop or choose not to answer troubling questions. Included with the study information is mental wellbeing support services that are available to access.

Are there any benefits to being involved?

The completion of this survey will not provide any academic benefit at your respective institutions. However, the results of SPICE-20 will be used to inform university wellbeing service provision. This means that by participating in the study you have the opportunity to provide anonymous feedback to your institution about what you feel is being done well, or could be done better to support students. Additionally, this survey may provide be useful as a personal opportunity for reflection.

How will my data be used?

Your answers will be completely anonymous and no identifiable data will be included. Data will be stored in a password protected file and will only be accessible by individuals on the steering/writing committee that have a reason to access it. IP addresses will not be stored. If you provide your email address, it will be deleted at the end of the study and not linked to any information you provide.

Anonymised responses to the survey will be stored for a minimum of ten years after publication or public release.

Who will have access to your data?

Qualtrics is the data platform used for SPICE-20. They are the data controller in respect to the personal data they hold and as such they determine how personal data is used. Their privacy notice can be found at: www.qualtrics.com/privacy-statement. Qualtrics will share any email address you provide with the University of Oxford for the purposes of research only. Responsible members of the University of Oxford may be given access to your data for monitoring or audit of the study and ensuring that guidelines are complied with.

Where will the results be published?

The results of SPICE-20 may be published in a peer reviewed academic journal. In addition, the results of the study may be presented at conferences, either in oral or poster format. The study is targeting a completion date of September 2021. Following this provisional date a summary of the results and outcomes will be available on request to the study team.

Who do I contact if I have any queries or wish to make a complaint about the study?

Our study team aims to operate in a transparent manner. If you have any queries or complaints regarding the study aims, distribution, or any other aspect please contact the study team at kate.saunders@psych.ox.ac.uk and we will do our best to provide an adequate response. Our research team aims to reply within 10 working days. If you have any further issues or wish to escalate your inquiry please contact the Chair of the Medical Sciences Inter-Divisional Research Ethics Committee. Email: ethics@medsci.ox.ac.uk. Address: Research Services, University of Oxford, Wellington Square, Oxford, OX1 2JD.

Who do I contact for further details?

For any queries or other details, contact the principle investigator Professor Kate Saunders at kate.saunders@psych.ox.ac.uk.

Please check the box to confirm you are the age of 18 years old or older

☐ I certify that I am 18 years or older.

If you have read the information above and agree to participate with the understanding that the data (including personal data) you submit will be processed accordingly, please check the relevant box to get started

☐ Yes, I agree to take part.
