# Supplementary material for: Social and psychological impact of the COVID-19 pandemic on UK medical and nursing students: protocol for a national medical and nursing student survey
Source: BMJ Open. 2022 May 6;12(5):e057467. doi: 10.1136/bmjopen-2021-057467 (PMC9082730; doi:10.1136/bmjopen-2021-057467)
Supplement: Author's manuscript [file bmjopen-2021-057467.draft_revisions.pdf]

BMJ Open is committed to open peer review. As part of this commitment we make the peer review history of every article we publish publicly available.

When an article is published we post the peer reviewers' comments and the authors' responses online. We also post the versions of the paper that were used during peer review. These are the versions that the peer review comments apply to.

The versions of the paper that follow are the versions that were submitted during the peer review process. They are not the versions of record or the final published versions. They should not be cited or distributed as the published version of this manuscript.

BMJ Open is an open access journal and the full, final, typeset and author-corrected version of record of the manuscript is available on our site with no access controls, subscription charges or pay-per-view fees (http://bmjopen.bmj.com).

If you have any questions on BMJ Open's open peer review process please email info.bmjopen@bmj.com

BMJ Open

Social and Psychological Impact of the Covid-19 pandemic on UK medical and nursing students: Protocol for a national medical and nursing student survey

Journal:	*BMJ Open*
Manuscript ID	bmjopen-2021-057467
Article Type:	Protocol
Date Submitted by the Author:	23-Sep-2021
Complete List of Authors:	Richardson, George; University of Liverpool School of Medicine, Institute of Translational Medicine<br>Gillespie, Conor; University of Liverpool, Institute of Systems Molecular and Integrative Biology<br>Mantle, Orla; King's College London Faculty of Life Sciences and Medicine<br>Clynch, Abigail; University of Liverpool, Institute of Systems Molecular and Integrative Biology<br>Ooi, Setthasorn Zhi Yang; Cardiff University School of Medicine<br>Park, Jay; The University of Edinburgh Edinburgh Medical School<br>Bligh, Emily R.; The University of Sheffield, School of Medicine<br>Kundu, Shantanu; University of Liverpool, School of Medicine<br>Georgiou, Ioannis; University of Aberdeen, School of Medicine<br>Bandyopadhyay, Soham; University of Oxford, Oxford University Medical School<br>Saunders, Kate E ; University of Oxford, Department of Psychiatry; Oxford Health NHS Foundation Trust
Keywords:	MENTAL HEALTH, MEDICAL EDUCATION & TRAINING, Child & adolescent psychiatry < PSYCHIATRY

SCHOLARONE™
Manuscripts

1

Social and Psychological Impact of the Covid-19 pandemic on UK medical and nursing students: Protocol for a national medical and nursing student survey

Authors and affiliations:

George E. Richardson*[1], Conor S. Gillespie*^[2], Orla Mantle[3], Abigail Clynch[2], Setthasorn Z. Y. Ooi[4], Jay Park[5], Emily R. Bligh[6], Shantanu Kundu[7], Ioannis Georgiou[8], Soham Bandyopadhyay[9], Kate E.A. Saunders[10,11], Neurology and Neurosurgery Interest Group (NANSIG), SPICE-20 Collaborative.

[1]Institute of Translational Medicine, University of Liverpool, Liverpool, UK

[2]Institute of Systems, Molecular and Integrative Biology, University of Liverpool, Liverpool, UK

[3]Faculty of Life Sciences and Medicine, King's College London, London, United Kingdom

[4]Cardiff University School of Medicine, University Hospital of Wales Main Building, Heath Park, Cardiff, UK

[5]Edinburgh Medical School, University of Edinburgh, Edinburgh, UK

[6]School of Medicine, University of Sheffield, Sheffield, UK

[7]School of Medicine, University of Liverpool, Liverpool, UK

[8]School of Medicine, University of Aberdeen, Aberdeen, UK

[9]Oxford University Global Surgery Group, Nuffield Department of Surgical Sciences, Medical Sciences Division, University of Oxford, Oxford, UK.

[10]Department of Psychiatry, Warneford Hospital, University of Oxford, Warneford Lane, Oxford, UK

[11]Oxford Health NHS Foundation Trust, Warneford Hospital, University of Oxford, Warneford Lane, Oxford, UK

*Joint first author, each author contributed equally to the work.

^Corresponding author

Abstract word count: 296

Full text word count: 2332

Corresponding author contact details:

Conor S. Gillespie
Institute of Systems, Molecular and Integrative Biology (ISMIB)
University of Liverpool
Biosciences Building
Crown Street
Liverpool
L69 7BE
Email address: hlcgill2@liv.ac.uk

Keywords: Wellbeing, Mental Health, Survey, Medical Students

ABSTRACT

Introduction

Healthcare students have played a significant role in the NHS during the COVID-19 pandemic. We captured data on the wellbeing of medical students during the acute phase of the pandemic with the Social and Psychological Impact of COVID-19 on medical students: a national survey Evaluation (SPICE-19) study. We will evaluate changes in mental health and wellbeing of medical and nursing students one year after the study in order to understand the ongoing impact of the pandemic, and inform wellbeing policies.

Methods and Analysis

This study will be a national, multi-institution, cross-discipline study. An online, 53-item survey of demographics, mental health and wellbeing will be used to record responses. Students studying for a medical or nursing degree at any UK universities will be eligible to participate. The survey will be advertised through the Neurology and Neurosurgery Interest Group (NANSIG) national network. Participation is anonymous and voluntary, with relevant mental health resources made available to participants.

Ethics and Dissemination

Ethical approval was granted by the University of Oxford Central University Research Ethics Committee (R75719/RE001) on 21st May 2021. The findings of the study will be presented at national and international meetings, and submitted for publication in a peer-reviewed journal.

Strengths and limitations

- This is a multi-centre, national questionnaire-based survey of medical and nursing student mental health and wellbeing in the UK.

- This is the first study to assess mental health and wellbeing of the student multi-disciplinary team during the new normal after the Covid-19 pandemic.

For peer review only

3

- The study results will provide valuable insight into healthcare workers, which can be used to identify and target further areas of wellbeing support and inform policy.

- Response bias may overestimate participation of students based on age, sex, and medical/nursing school, with those interested in neuroscience and mental health more likely to participate.

INTRODUCTION

Medical and nursing students in the UK have been significantly affected by the COVID-19 pandemic. During the first wave, many were active components of the clinical team, either by undertaking extending clinical placements, paid work in the NHS, or through voluntary roles, while others saw placements reduced, suspended, or cancelled completely.(1, 2) We evaluated medical student wellbeing and mental health during the acute phase of the pandemic with the Social and Psychological Impact of COVID-19 on medical students: a national service evaluation (SPICE-19) study,(3) a cross sectional study with a prospective component, which identified several points for service provision improvement based on 2275 responses across 34 medical schools in the UK.(4-6)

It is pertinent to re-evaluate the mental health of medical students, one year removed from SPICE-19, to explore how they are navigating their training, education, and the impact of adapting to the 'new normal' of medical education provision.(7, 8) It is also important to elucidate the effect of COVID-19 on the wellbeing and mental health of UK nursing students, many of whom were asked to volunteer on the front line in COVID wards,(2) and a large-scale study examining this has not been completed. A single centre survey in the US noted that nursing students reported increased levels of anxiety, reduction in concentration, and difficulties with academic workload throughout the pandemic,(9) and a large multi-institutional study is required to elucidate this further. This is in accordance with previously published work by the SPICE-19 research team. (10, 11)

Medical and nursing schools have each responded differently to the pandemic, with the accessibility of wellbeing and mental health services variable between universities.(12) A national survey assessing mental health and wellbeing in medical and nursing students, as well as support systems available for those facing mental health difficulty is required. Evaluating the mental health and wellbeing of the student multi-disciplinary team is vital for

For peer review only

ensuring adequate service provision is provided - it is paramount to safeguard the wellbeing of the next generation of healthcare professionals.(13, 14)

The primary aim of this study is to comprehensively evaluate mental health and wellbeing of medical and nursing students during the evolving COVID-19 pandemic, one year after the initial outbreak. The secondary aims are to elucidate students' perceptions of institutional efforts to provide support, and accessibility and appropriateness of such support. The study logos are shown in Figure 1.

Figure 1. SPICE-20 study logos.

METHODS AND ANALYSIS

<u>Study partners</u>

NANSIG

The Neurology and Neurosurgery Interest Group (NANSIG) was formed in 2010. It is a student and junior doctor led interest group designed to increase participation, diversity and engagement in Neurosciences, and has over 1000 members internationally.(15) The organisation has an affiliation with the Society of British Neurosurgeons (SBNS) and the Association of British Neurologists (ABN) (www.nansig.org). The organisation led the SPICE-19 national cohort study last year, assessing wellbeing in 2275 medical students in response to the pandemic, and is familiar with survey design and distribution. The collective organisation runs established national and international events, and has published over 20 peer-reviewed publications.

<u>Study design</u>

SPICE-20 is a national, multi-institution, cross-sectional study. An online survey will be used to record participant responses. The survey contains 53 items - section 1 of the survey includes background and demographic information. Section 2 assesses wellbeing by asking participants to complete the Warwick-Edinburgh Mental Well Being Scale (WEMWBS); a 14-point, validated evaluation of wellbeing.(16) Section 3 encompasses the Patient Health Questionnaire-9 (PHQ-9) and Generalised Anxiety Disorder-7 (GAD-7). The PHQ-9 is a valid diagnostic and severity measure for depressive disorder,(17) and GAD-7 is a seven-item,

For peer review only

5

validated assessment of generalised anxiety.(18, 19) Section 4 includes questions about university support offered by each institution, if participants had accessed this support, and any significant changes to teaching encountered during the pandemic.

The survey was iteratively defined by the student-led study management team until a consensus was reached. Based on our experience with SPICE-19 survey, we have reduced the number of questions and limited the survey to validated measures of wellbeing and mental health. The final, 53-item questionnaire is shown in Figure 2.

Figure 2: Final SPICE-20 survey design.

<u>Survey piloting</u>

The survey was piloted by a group of 15 medical students from the NANSIG core committee. These students were not involved in the design of the study and were consulted in order to provide feedback, improve clarity and ensure objectivity. Data was analysed to identify any points of concern, estimated completion time, and difficult survey items/questions. Students were contacted to identify any suggestions for improvement, with none identified. Therefore, no further alterations were made.

<u>Survey administration</u>

The survey was hosted on the Qualtrics™ survey platform (Provo, Utah, USA), a GDPR-compliant online survey platform, that facilitates both mobile and desktop devices.

<u>Study dissemination</u>

To maximise distribution across the UK, a national network of SPICE-20 Collaborative members were recruited, representing all 33 medical schools in the UK. Most held concurrent committee or regional lead roles in NANSIG at time of recruitment. Each member was asked to acquire the most up to date (as of April 2021) available resources, guidance and support policies in place for their current medical school, plus an additional nursing school. A standard advertising method was developed by the steering committee and based on the successful recruitment strategy employed in the SPICE-19 study, which involved

For peer review only

members contacting their respective medical school deans to request distribution of the survey at inception, as well as regular advertising over the 8-week study collection period, from 7st June 2021 to the 7rd August 2021. The distribution links for the survey includes university mailing lists, student society pages, and social media platforms. Figure 3 provides an example of the email template used to disseminate the survey through student mailing lists. Adverts will also be placed on NANSIG social media platforms, as well as the monthly NANSIG newsletter.

In the invitation to complete the study, based on their medical/nursing school, participants will also receive wellbeing, mental health, and guidance services specific to their institution, should they need to access them at any point. Participants will also be made aware of our patient and public involvement organisation, and their contact details. No incentives for participation will be offered.

Figure 3. Standard email template used for all survey dissemination through student email lists, developed by the study team and approved by the research ethics committee.

Eligibility and representation

All current students enrolled at UK medical schools recognised by the General Medical Council (GMC) and the Medical Schools Council (MSC) will be eligible to participate. Additionally, all current home and international students enrolled on an adult, child and mental health nursing degree at a UK university are eligible for inclusion. A list of eligible medical schools, and approved programmes by the Nursing and Midwifery Council (NMC) is included in the supplementary materials (document “Supplementary Material – MSC and NMC”).

Consent and confidentiality

A patient information sheet will be shown on the first page of the study (supplementary material - patient information). This encompasses the rationale, purpose, and voluntary nature of participation. Participants must verify that they are over 18 years old and provide informed consent via tick box. It will be emphasised that participation is anonymous, confidential, voluntary, and that participants can withdraw consent at any time.

Data security

For peer review only

All data will be collected and stored on the secure online server, Qualtrics™. Survey data will be extracted from the software to a password-protected, Microsoft Excel spreadsheet (Microsoft, California, USA) only available to the study team. Data handling and record keeping will adhere to the University of Oxford standards for data security.

Statistical analysis

Data will be analysed using SPSS statistics V25 and presented using RStudio V4.2. The overall wellbeing of medical and nursing students, as assessed using the WEMWBS, and mental health, using the PHQ-9 and GAD-7, will be presented using descriptive statistics. No information that identifies or is specific to institutions will be presented. Appropriate statistical methods will be selected based on the distribution and type of data; it is anticipated data will be non-parametric, and, will therefore be compared using tests such as $\chi2$ or Kruskal-Wallis tests. Differences between medical and nursing students will be compared using these statistical tests.

Sample size calculation

Approximate estimates of total UK medical and nursing students were calculated from yearly undergraduate intake figures. The total number of UK medical and adult nursing students was estimated to be 84,000. An online calculator was used to determine the sample size needed for the survey. The minimum sample size required from our target population for a margin error of 5% and a confidence interval level of 95% is 383, assuming a 50% response distribution for each question. For every medical student response, 1.28 nursing student responses would be needed to achieve a 50:50 distribution in response rate.

Patient and public involvement

As the study population was medical and nursing student wellbeing and mental health, we worked collaboratively with the Be Free Campaign - a mental health and wellbeing awareness charity, from study inception. Be Free were involved in the conception, design and development of the study. The charity works with young people and students, emphasising mental health and wellbeing, to tackle the stigma behind mental health, and promote expression of individual values (UK registered charity number 1189704). The

For peer review only

charity director and clinical study team for the charity comprehensively reviewed the protocol for appropriateness of content, question format, and ensured the questions asked were sensitive and pertinent to medical and nursing students.

Be Free have extensive knowledge of indicators of mental health, and the questions selected (WEMWBS, PHQ-9 and GAD-7) were done so in concordance with their advice. The number of questions, layout, and design were also prepared in accordance with their suggestions. The charity inspired the conception of this study and approved the final survey design. The charity founder and director (SK) is also a member of the steering committee for this study.

DISCUSSION

Study rationale

This is the first national, multi-institutional study to evaluate the mental health of UK medical and nursing students during the COVID-19 pandemic.(20) It will therefore be possible to elucidate the mental health and wellbeing of healthcare students across the UK. By comparing medical and nursing schools, it will be possible to evaluate the nature of successful support, as well as students' confidence and ability to access this support. The findings will inform service provision, and highlight areas where improvement is required. The survey will also highlight local and national wellbeing resources available to students.

Why assess medical and nursing student wellbeing and mental health?

Medical and nursing students form the healthcare multi-disciplinary team of the future. Mental health and wellbeing are positively correlated with job satisfaction, retention, and performance, and is an essential component of good health.(21) If the NHS is to retain, recruit and develop its workforce of the future, ascertaining their mental health at this point in the COVID-19 pandemic is essential. Young people have been disproportionately affected by the pandemic, and most medical and nursing students fall into this age category. As non-emergency care is delayed and rescheduled, students will have to deal with the long acting repercussions of the pandemic, including those directly affected by the virus itself, including

For peer review only

long-COVID,(22) declining mental health in the population exacerbated by the pandemic,(23) and implications of delayed access to healthcare.(24, 25)

Many medical students may not be aware that such support exists, either at their respective institution, or at a regional/national level, and we would do these students a disservice by not highlighting existing mental health and wellbeing services.

Benefits of a national medical student wellbeing survey

Medical and nursing schools across the UK adopt heterogenous practices to achieve the outcomes set in the GMC and NMC core competencies and curricula.(26) A survey of both medical and nursing students on a national scale is the best way to evaluate mental health and wellbeing. Many institutions reacted differently to the pandemic, and a survey is best placed to simultaneously decipher wellbeing and mental health at each institution, while assessing the impact of the pandemic on a national scale, and highlighting heterogeneity between institutions. Furthermore, there is a need to appreciate that multi-institutional studies of large magnitude are required to answer important clinical questions and elucidate concerns not identified by smaller studies. Multi-centre studies are also less susceptible to identity issues because the data is aggregated between multiple medical schools, and can be presented as all centres.

Limitations

Online surveys have the advantage of ubiquitous presence, and increased accessibility to medical and nursing schools in the UK. However, several limitations can arise from such a study method.

Firstly, those with an existing interest in wellbeing and mental health may be more likely to engage with and complete the survey. This could lead to selection bias towards those adversely affected by the pandemic. The anticipated magnitude of this effect is unclear. In addition, as the survey is being distributed through NANSIG, there may be over-participation from students with an interest in Neurosciences and their surrounding connections.

In order to ameliorate this as best as possible, we have adopted a varied dissemination approach. This includes having representatives at each UK medical school, survey distribution to medical and nursing school bulletins, and newsletters. We hope this will

For peer review only

improve visibility and participation. We decided not to incentivise participants with a potential prize for completing the survey, as we wanted responses to be motivated by a desire to complete the questions in as open a way as possible.

The study is also limited by its cross-sectional design, and will therefore only provide a snapshot of mental health and wellbeing at the time of survey completion. In addition, during capricious times such as COVID, fluctuations in mental health may occur in accordance with policy changes, as well as institutional policies towards placements and assessment, and we will assess date of study completion as a potential confounder as a variable in our analysis.

The self-reporting of symptoms, will also be a significant limitation, in addition to the fact that the questions included are screening tools for mental health and wellbeing, and are not immediately diagnostic.

Ethics and dissemination

Ethical approval for this study was granted by the Oxford Research ethics committee [R75719/RE001]. The findings of the SPICE-20 study as described in this protocol, will be presented at scientific conferences, and submitted for publication in a peer-review journal once finalised.

Acknowledgments

The authors would like to thank the SPICE-19 collaborative, whose work inspired this study, and are grateful to the Be Free Campaign for their assistance with the conception, design and inspiration for components of the study. We also thank NANSIG and members of the SPICE-20 collaborative, for their kind assistance disseminating the survey.

Funding statement

No external funding has been sought in the conception, design or implementation of this study. CSG is a recipient of a grant from the Wolfson Foundation. GER is the recipient of funding from North West Cancer Research. KS is supported by the NIHR Oxford Health Biomedical Research Centre. The views expressed are those of the author(s) and not necessarily those of the NHS, the NIHR or the Department of Health.

For peer review only

Competing interests' statement

None to declare.

Presented at: N.A.

Author Contributions

GER, CSG, and OM were responsible for initial drafting of protocol manuscript, conceptualisation and designing the study. AC, SZYO, JP, ERB, IG, SB, and KS formed the wider study group and reviewed and approved drafts of the manuscript. KS provided supervision of the project. SK reviewed the manuscript and also provided PPI involvement and approval for the study.

Data Sharing Statement

Data are available upon reasonable request. All data must be anonymised entirely prior to any sharing request completion.

For peer review only

12

REFERENCES

1. Miller DG, Pierson L, Doernberg S. The Role of Medical Students During the COVID-19 Pandemic. Annals of Internal Medicine. 2020;173(2):145-6.
2. Swift A, Banks L, Baleswaran A, Cooke N, Little C, McGrath L, et al. COVID-19 and student nurses: A view from England. Journal of Clinical Nursing. 2020;29(17-18):3111-4.
3. Soham B, Ioannis G, Bibire B, Conor SG, Marta de Andres C, Mohammad Talha B, et al. Research Square. 2021.
4. Coyle C, Ghazi H, Georgiou I. The mental health and well-being benefits of exercise during the COVID-19 pandemic: a cross-sectional study of medical students and newly qualified doctors in the UK. Irish Journal of Medical Science (1971 -). 2020.
5. Norton EJ, Georgiou I, Fung A, Nazari A, Bandyopadhyay S, Saunders KEA. Personal protective equipment and infection prevention and control: a national survey of UK medical students and interim foundation doctors during the COVID-19 pandemic. Journal of Public Health. 2020.
6. Georgiou I, Hounat A, Park JJ, Gillespie C, Bandyopadhyay S, Saunders KEA. The Factors That Influenced Medical Students' Decision to Work Within the NHS During the COVID-19 Pandemic-A National, Cross-sectional Study. J Occup Environ Med. 2021;63(4):296-301.
7. Lucey CR, Johnston SC. The Transformational Effects of COVID-19 on Medical Education. JAMA. 2020;324(11):1033-4.
8. Yuen J, Xie F. Medical education during the COVID-19 pandemic: perspectives from UK trainees. Postgraduate Medical Journal. 2020;96(1137):432.
9. Fitzgerald A, Konrad S. Transition in learning during COVID-19: Student nurse anxiety, stress, and resource support. Nurs Forum. 2021:10.1111/nuf.12547.
10. Coyle C, Ghazi H, Georgiou I. The mental health and well-being benefits of exercise during the COVID-19 pandemic: a cross-sectional study of medical students and newly qualified doctors in the UK. Ir J Med Sci. 2020:1-2.
11. Norton EJ, Georgiou I, Fung A, Nazari A, Bandyopadhyay S, Saunders KEA. Personal protective equipment and infection prevention and control: a national survey of UK medical students and interim foundation doctors during the COVID-19 pandemic. J Public Health (Oxf). 2021;43(1):67-75.
12. Arora A, Solomou G, Bandyopadhyay S, Simons J, Osborne A, Georgiou I, et al. Adjusting to Disrupted Assessments, Placements and Teaching (ADAPT): a snapshot of the early response by UK medical schools to COVID-19. medRxiv. 2020:2020.07.29.20163907.
13. Kinder F, Harvey A. Covid-19: the medical students responding to the pandemic. BMJ. 2020;369:m2160.
14. Karaca A, Yildirim N, Cangur S, Acikgoz F, Akkus D. Relationship between mental health of nursing students and coping, self-esteem and social support. Nurse Educ Today. 2019;76:44-50.

For peer review only

13

15. Gillespie CS, Bandyopadhyay S, Jenkins AJ, Hutchinson P. Inspiring the next generation. The Lancet Neurology. 2021;20(4):256-7.
16. Tennant R, Hiller L, Fishwick R, Platt S, Joseph S, Weich S, et al. The Warwick-Edinburgh Mental Well-being Scale (WEMWBS): development and UK validation. Health Qual Life Outcomes. 2007;5:63.
17. Kroenke K, Strine TW, Spitzer RL, Williams JB, Berry JT, Mokdad AH. The PHQ-8 as a measure of current depression in the general population. J Affect Disord. 2009;114(1-3):163-73.
18. Spitzer RL, Kroenke K, Williams JBW, Löwe B. A Brief Measure for Assessing Generalized Anxiety Disorder: The GAD-7. Archives of Internal Medicine. 2006;166(10):1092-7.
19. Löwe B, Decker O, Müller S, Brähler E, Schellberg D, Herzog W, et al. Validation and standardization of the Generalized Anxiety Disorder Screener (GAD-7) in the general population. Med Care. 2008;46(3):266-74.
20. Lyons Z, Wilcox H, Leung L, Dearsley O. COVID-19 and the mental well-being of Australian medical students: impact, concerns and coping strategies used. Australasian Psychiatry. 2020;28(6):649-52.
21. Spoorthy MS, Pratapa SK, Mahant S. Mental health problems faced by healthcare workers due to the COVID-19 pandemic-A review. Asian J Psychiatr. 2020;51:102119-.
22. Mandal S, Barnett J, Brill SE, Brown JS, Denneny EK, Hare SS, et al. 'Long-COVID': a cross-sectional study of persisting symptoms, biomarker and imaging abnormalities following hospitalisation for COVID-19. Thorax. 2020:thoraxjnl-2020-215818.
23. Xiong J, Lipsitz O, Nasri F, Lui LMW, Gill H, Phan L, et al. Impact of COVID-19 pandemic on mental health in the general population: A systematic review. J Affect Disord. 2020;277:55-64.
24. Lazzerini M, Barbi E, Apicella A, Marchetti F, Cardinale F, Trobia G. Delayed access or provision of care in Italy resulting from fear of COVID-19. Lancet Child Adolesc Health. 2020;4(5):e10-e1.
25. Czeisler M, Marynak K, Clarke KEN, Salah Z, Shakya I, Thierry JM, et al. Delay or Avoidance of Medical Care Because of COVID-19-Related Concerns - United States, June 2020. MMWR Morb Mortal Wkly Rep. 2020;69(36):1250-7.
26. McManus IC, Harborne AC, Horsfall HL, Joseph T, Smith DT, Marshall-Andon T, et al. Exploring UK medical school differences: the MedDifs study of selection, teaching, student and F1 perceptions, postgraduate outcomes and fitness to practise. BMC Med. 2020;18(1):136.

For peer review only

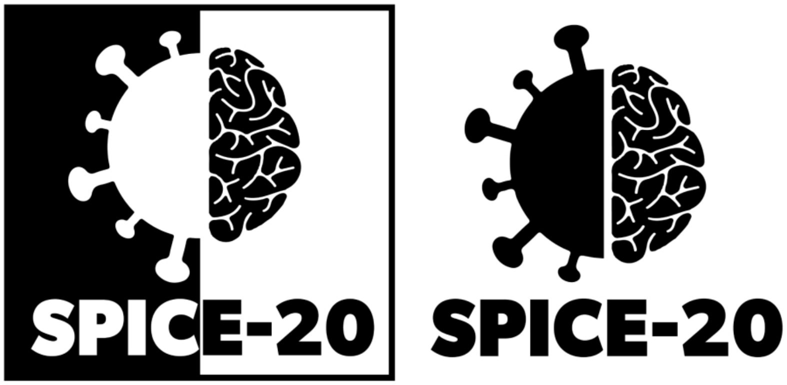

Figure 1. SPICE-20 study logos.

533x755mm (118 x 118 DPI)

Section 1 – Background Information

1. *Are you a medical or nursing student?*
    a. Medical student
        i. Which medical school are you currently enrolled in?
            1. Response from dropdown of medical schools
    b. Nursing Student
        i. Which nursing school are you currently enrolled in?
            1. Response from dropdown of nursing schools
2. How many years (if any) of your degree (excluding intercalation) have you completed so far?
    a. Choose year of study from list
3. Are you currently undertaking an intercalated degree?
    a. Respond yes or no
        i. Have you previously undertaken an intercalated degree?
            1. Respond yes or no
4. How old are you?
    a. Free text box for age
5. What is your gender?
    a. Choose from gender list with free-text box
6. What is your race or ethnicity?
    a. Choose from list of ethnicities with free-text box
7. What is your religion?
    a. Choose from list of religions with free-text box

Section 2 – Wellbeing and Mental Health

WEMWBS

Participants are shown a series of statements and asked to choose an appropriate response from either: 1) None of the time, 2) Rarely, 3) Some of the time, 4) Often and 5) All of the time

1. "I've been feeling optimistic about the future"
2. "I've been feeling useful"
3. "I've been feeling relaxed"
4. "I've been feeling interested in other people"
5. "I've had energy to spare"
6. "I've been dealing with problems well"
7. "I've been thinking clearly"
8. "I've been feeling good about myself"
9. "I've been feeling close to other people"
10. "I've been feeling confident"
11. "I've been able to make up my own mind about things"
12. "I've been feeling loved"
13. "I've been interested in new things"
14. "I've been cheerful"

PHQ-9

Participants are asked, over the last 2 weeks, how often have you been affected by any of the following problems? Responses include: 1) Not at all (0-1 days), 2) Several days (2-6 days), 3) More than half the days (7-11 days), 4) Nearly every day (12-14 days)

1. Little interest or pleasure in doing things?
2. Feeling down, depressed or hopeless?
3. Trouble falling or staying asleep, or sleeping too much?
4. Feeling tired or having little energy?
5. Poor appetite or overeating?
6. Feeling bad about yourself – or that you are a failure or have let yourself or your family down
7. Trouble concentrating on things, such as reading the newspaper or watching television?
8. Moving or speaking so slowly that other people could have noticed. Or the opposite – being so fidgety or restless that you have been moving around a lot more than usual?
9. Thoughts that you would be better off dead or of hurting yourself in some way?

GAD-7

Participants are asked, over the last 2 weeks, how often have you been bothered by the following problems? Responses include: 1) Not at all, 2) Several days, 3) More than half the days, 4) Nearly every day

1. Feeling nervous, anxious or on edge
2. Not being able to stop or control worrying
3. Worrying too much about different things
4. Trouble relaxing
5. Being so restless that it is hard to sit still
6. Becoming easily annoyed or irritable
7. Feeling afraid as if something awful might happen

Other Questions

1. How would you rate your mood before COVID-19 affected the UK (One year ago)? 0 being the worst mood you can imagine and 100 being the best mood you can imagine
    a. Response via sliding scale from 0 to 100
2. How would you rate your mood now? 0 being the worst mood you can imagine and 100 being the best mood you can imagine
    a. Response via sliding scale from 0 to 100

Section 3 – Support

1. Has your university provided any form of mental health and wellbeing support?
    a. Yes
        i. What support has the university provided you with? (please select one or more)
            1. Multiple selection boxes with options and free text box for other
        ii. How confident do you feel accessing the support services available to you?
            1. 5 point Likert scale from not confident at all to very confident
        iii. Have you accessed/used support that your university has provided?
            1. Yes
                a. Has your university support been useful?
                    i. Yes
                        1. In what way?
                            a. Free text response
                    ii. No
                        1. Why not?
                            a. Free text response
            2. No
        iv. Would you like your university to provide you with more wellbeing support?
            1. Yes (please select one or more)
                a. Multiple selection boxes with options and free text box for other
            2. No
                a. Why not?
                    i. Free text response
    b. No

Section 4 – Changes

1. Is your teaching different now compared to before the pandemic (do not answer if in first year)?
    a. Yes
        i. Please select one or more of the following ways teaching/placements have changed:
            1. Multiple selection boxes with options and free text box for other
    b. No
        i. Why not?
            1. Free text response
2. Has there been any positive aspects of the changes to teaching?
    a. Yes
        i. Please detail the positive changes to teaching
            1. Free text response
    b. No
3. What changes could be made to teaching in medical school to improve your wellbeing?
    a. Multiple selection boxes with options and free text box for other
4. Have you worked or are you currently working within a healthcare setting during the course of this pandemic?
    a. Yes
        i. Which of the following roles have you taken up?
            1. Multiple selection boxes with options and free text box for other
        ii. Have you been offered wellbeing support in response to the role you have undertaken?
            1. Yes
                a. Who has provided this support? (please select one or more)
                    i. Multiple selection boxes with options and free text box for other
            2. No
    b. No
5. After the pandemic, are you more or less likely to continue in a career in Medicine/Nursing?
    a. 5 point Likert scale from Much less likely to Much more likely
6. Have your future career plans changed since the start of the pandemic?
    a. Yes
        i. If Yes, how come?
            1. Free text response
    b. No

Figure 2: Final SPICE-20 survey design.

913x1291mm (118 x 118 DPI)

Dear Students,

We are a group of student researchers that are interested in investigating the current state of medical student mental health and wellbeing, one year on from the onset of the pandemic. We are inviting all UK medical students, from years 1 to 5 (and intercalating) to complete a short 15 minute survey to help us better understand how you are doing. Whilst completing the survey, you will be asked a variety of questions about your mental health and wellbeing, and the support that you have received. Your IP address will not be recorded and responses will be anonymous. There will not be any follow up survey. Useful links to support services have been provided along with the post-survey debrief. Additional information about the risks versus benefits of taking part in the survey can be found by following the study link below. **Please access the survey by the link below:**

https://psychiatryoxford.qualtrics.com/jfe/form/SV_eQmCH0FLewvbcWO

If you have any questions about the study or your involvement please contact a member of the study team at the following email and we will aim to reply within 24hrs: hlgricha@liverpool.ac.uk

Should you have concerns about your wellbeing do consider contacting you GP or accessing the resources available: <LOCAL SERVICE PROVISION INSERTED HERE>

Many thanks for taking the time to read this,

George Richardson
SPICE-20 Study Team

SPICE-20 NANSIG

Figure 3. Standard email template used for all survey dissemination through student email lists, developed by the study team and approved by the research ethics committee.

554x668mm (57 x 57 DPI)

UK N.M.C Approved Adult Nursing Programmes

Approved education institution	Course
**University Of Essex**	Adult Nursing
**University Of Essex**	Adult Nursing (nursing degree apprenticeship route)
**Edinburgh Napier University**	Bachelor of Nursing (Adult)
**University Of Wolverhampton**	Bachelor of Nursing (BNurs (Hons)) Adult Nursing
**University Of Wolverhampton**	Bachelor of Nursing (BNurs (Hons)) Adult Nursing [Apprenticeship]
**University Of Glasgow**	Bachelor of Nursing (Honours)
**University Of Manchester**	Bachelor of Nursing (Hons) (Adult)
**University Of Birmingham**	Bachelor of Nursing (Hons) (Adult)
**University Of Liverpool**	Bachelor of Nursing (Hons) Nursing (Adult)
**University Of Glasgow**	Bachelor of Nursing (ordinary) [fall back award]
**Cardiff University**	Bachelor of Nursing Adult
**University of Derby**	Bachelor of Science (Honours) in Nursing (Adult)
**University of Derby**	Bachelor of Science (Honours) in Nursing (adult) (apprenticeship)
**University Of Sheffield**	BMed Sci (Hons) Nursing (Adult)
**University Of Sheffield**	BMedSci (Hons) Nursing (Adult) Apprenticeship route
**University of Chester**	BN (Hons) Adult Nursing
**University of Chester**	BN (Hons) Adult Nursing
**University of Chester**	BN (Hons) Adult Nursing
**University of Chester**	BN (Hons) Adult Nursing
**University of Chester**	BN (Hons) Adult Nursing

For peer review only

Approved education institution	Course
University of Chester	BN (Hons) Adult Nursing Apprenticeship
University of Chester	BN (Hons) Adult Nursing Apprenticeship
University Of Portsmouth	BN (Hons) Nursing (Adult)
University of Winchester	BN (Hons) Nursing (Adult)
Glyndwr University (formerly North East Wales Inst. of HE)	BN Nursing
University Of Edinburgh	BN nursing (adult)
University Of Edinburgh	BN nursing with Honours (adult)
University of Chester	BNurs (Hons) Adult
University of Chester	BNurs (Hons) Adult (part-time)
University of Chester	BNurs (Hons) Adult Apprenticeship
University Of Wolverhampton	BNurs (Hons) Adult Nursing
University Of Wolverhampton	BNurs (Hons) Adult Nursing Apprenticeship
University of West London	BNursing (Hons) Adult
University Of Hertfordshire	BSc Hons Nursing (Adult)
University Of Stirling	BSc (Honours) Nursing (Adult)
Coventry University	BSc (Hons) Adult Nursing
Coventry University	BSc (Hons) Adult Nursing (Degree apprenticeship)
Oxford Brookes University	BSc (Hons) (Adult)
London South Bank University	BSc (Hons) adult nursing
University of Suffolk	BSc (Hons) Adult Nursing
University of West of Scotland	BSc (Hons) Adult Nursing
Leeds Beckett University	BSc (Hons) Adult Nursing
University of Northampton	BSc (Hons) Adult Nursing
Bournemouth University	BSc (Hons) Adult Nursing

For peer review only

Approved education institution	Course
**University of Roehampton**	BSc (Hons) Adult Nursing
**University Of Dundee**	BSc (Hons) Adult Nursing
**University of West of Scotland**	BSc (Hons) Adult Nursing
**University Of Greenwich**	BSc (Hons) Adult Nursing
**University of Bedfordshire**	BSc (Hons) Adult Nursing
**Bournemouth University**	BSc (Hons) Adult Nursing (Apprenticeship)
**University of Bedfordshire**	BSc (Hons) Adult Nursing (apprenticeship)
**University of Sunderland**	BSc (Hons) Adult Nursing (blended)
**University of Sunderland**	BSc (Hons) Adult Nursing (blended)
**Leeds Beckett University**	BSc (Hons) Adult Nursing (degree apprenticeship)
**Manchester Metropolitan University**	BSc (Hons) Adult Nursing (Pre-registration)
**University of Suffolk**	BSc (Hons) Adult Nursing [degree apprenticeship]
**Coventry University**	BSc (Hons) Adult Nursing Blended Learning
**Coventry University**	BSc (Hons) Adult Nursing Blended Learning Nursing Degree Apprenticeship
**University Of Greenwich**	BSc (Hons) Adult Nursing Degree Apprenticeship
**Solent University**	BSc (Hons) Adult Nursing Practice – apprenticeship
**University of Sunderland**	BSc (Hons) adult nursing practice
**Solent University**	BSc (Hons) Adult Nursing Practice
**University Of Huddersfield**	BSc (Hons) Degree Apprenticeship - Registered Nurse Child (Blended Learning)
**University Of Huddersfield**	BSc (Hons) Degree Apprenticeship-Registered Nurse: Adult

For peer review only - http://bmjopen.bmj.com/site/about/guidelines.xhtml

Approved education institution	Course
**BPP**	BSc (Hons) Nursing (Adult) [Nursing Degree Apprenticeship]
**BPP**	BSc (Hons) Nursing (Adult) [pre registration nursing]
**University of East Anglia**	BSc (Hons) nursing - Adult
**University Of Huddersfield**	BSc (Hons) Nursing - Adult
**University Of The West Of England, Bristol**	BSc (Hons) Nursing - Adult
**University of East Anglia**	BSc (Hons) Nursing - Adult Degree Apprenticeship
**Queen Margaret University**	BSc (Hons) Nursing
**University Of Plymouth**	BSc (Hons) Nursing (Adult Health)
**University Of Plymouth**	BSc (Hons) Nursing (Adult Health) - Nurse Degree Apprenticeship
**University Of York**	BSc (hons) Nursing (Adult)
**University of Salford**	BSc (Hons) Nursing (adult)
**University of Bolton**	BSc (Hons) Nursing (adult)
**University of Nottingham**	BSc (Hons) Nursing (adult)
**Buckinghamshire New University**	BSc (Hons) Nursing (Adult)
**Anglia Ruskin University**	BSc (Hons) Nursing (Adult)
**Swansea University**	BSc (Hons) Nursing (Adult)
**Edge Hill University**	BSc (Hons) Nursing (Adult)
**Edge Hill University**	BSc (Hons) Nursing (Adult)
**The Open University**	BSc (Hons) Nursing (Adult)
**The University of Hull**	BSc (Hons) Nursing (Adult)
**The University of Hull**	BSc (Hons) Nursing (Adult)
**University of Ulster**	BSc (Hons) Nursing (Adult)

For peer review only

Approved education institution	Course
**University Of East London**	BSc (Hons) Nursing (Adult)
**University Of Leeds**	BSc (Hons) Nursing (Adult)
**University College Birmingham**	BSc (Hons) Nursing (Adult)
**University Of Southampton**	BSc (Hons) Nursing (Adult)
**Canterbury Christ Church University**	BSc (Hons) Nursing (Adult)
**Birmingham City University**	BSc (Hons) Nursing (Adult)
**Anglia Ruskin University**	BSc (Hons) Nursing (Adult)
**Swansea University**	BSc (Hons) Nursing (Adult)
**The Robert Gordon University**	BSc (Hons) Nursing (Adult)
**University of Bradford**	BSc (Hons) Nursing (Adult)
**The Open University**	BSc (Hons) Nursing (Adult)
**The Open University**	BSc (Hons) Nursing (Adult)
**University of Gloucestershire**	BSc (Hons) Nursing (Adult)
**University Of Hertfordshire**	BSc (Hons) Nursing (adult) - Nursing Degree Apprenticeship
**The Open University**	BSc (Hons) Nursing (Adult) - Nursing Degree Apprenticeship
**Birmingham City University**	BSc (Hons) Nursing (Adult) - Nursing Degree Apprenticeship
**University of Gloucestershire**	BSc (Hons) Nursing (Adult) (Apprenticeship)
**University of Gloucestershire**	BSc (Hons) Nursing (Adult) (Blended Learning)
**Edge Hill University**	BSc (Hons) Nursing (Adult) (fall back award)
**University Of Leeds**	BSc (Hons) Nursing (Adult) (International)
**Canterbury Christ Church University**	BSc (Hons) Nursing (Adult) (NDA)
**University of Bolton**	BSc (Hons) Nursing (Adult) (Part-time)

For peer review only

Approved education institution	Course
**University of Bolton**	BSc (Hons) Nursing (Adult) (Registered Nursing Higher Apprenticeship) (Part-time)
**University of Bradford**	BSc (Hons) Nursing (Adult) [fall back award]
**Buckinghamshire New University**	BSc (Hons) Nursing (Adult) Apprenticeship
**University of Salford**	BSc (Hons) Nursing (adult) Nurse Degree Apprentice
**University of Bolton**	BSc (Hons) Nursing (adult) nurse degree apprenticeship (NDA) route
**Middlesex University**	BSc (Hons) Nursing (adult) seconded pathway
**De Montfort University**	BSc (Hons) Nursing (Adult) with NMC Registration (Apprenticeship route)
**University Of East London**	BSc (Hons) Nursing (Adult)-Registered Nurse (Degree) Apprenticeship Route
**Queen Margaret University**	BSc (Hons) Nursing (fall back award)
**University of Lincoln**	BSc (Hons) Nursing (Registered Nurse - Adult)
**University of South Wales**	BSc (Hons) Nursing Adult
**Nottingham Trent University**	BSc (Hons) Nursing Adult
**University Of Brighton**	BSc (Hons) Nursing Adult
**University of Worcester**	BSc (Hons) Nursing Adult
**Sheffield Hallam University**	BSc (Hons) Nursing Adult (Part Time)
**University Of The West Of England, Bristol**	BSc (Hons) Nursing apprenticeship - Adult
**University Of Huddersfield**	BSc (Hons) Nursing Child (Blended Learning)
**University of Sunderland**	BSc (Hons) nursing degree apprenticeship (adult)
**Nottingham Trent University**	BSc (Hons) Nursing Degree Apprenticeship Adult
**Swansea University**	BSc (Hons) Nursing part-time (Adult)

For peer review only

Approved education institution	Course
**University Of Northumbria At Newcastle**	BSc (Hons) nursing science (adult)
**University Of Northumbria At Newcastle**	BSc (Hons) nursing science apprenticeship (adult)
**Teesside University**	BSc (Hons) Nursing Studies (Adult) (Pre-registration) Apprenticeship route
**Glasgow Caledonian University**	BSc (Hons) Nursing Studies: Adult Nursing
**Liverpool John Moores University**	BSc (Hons) Nursing with Registered Nurse Status (Adult)
**Liverpool John Moores University**	BSc (Hons) Nursing with Registered Nurse Status (Adult)
**University of Cumbria**	BSc (Hons) Nursing/RN:Adult Nursing
**University of Cumbria**	BSc (Hons) Nursing/RN:Adult Nursing (Apprenticeship)
**Keele University**	BSc (Hons) Nursing: Adult
**Keele University**	BSc (Hons) Nursing: Adult (NDA)
**Kingston University & St George's University of London**	BSc Adult Nursing
**University Of Dundee**	BSc Adult Nursing
**University of West of Scotland**	BSc Adult Nursing
**University Of Greenwich**	BSc Adult Nursing (Degree Apprenticeship) (fall back award)
**University Of Greenwich**	BSc Adult Nursing (fall back award)
**Middlesex University**	BSc Hons Nursing Adult
**University of Highlands and Islands**	BSc Nursing
**City, University of London**	BSc Nursing (Adult)
**Oxford Brookes University**	BSc Nursing (Adult)
**The Robert Gordon University**	BSc Nursing (Adult)

For peer review only

Approved education institution	Course
**The Robert Gordon University**	BSc Nursing (Adult)
**University Of Stirling**	BSc Nursing (Adult)
**The Robert Gordon University**	BSc Nursing (Adult) (fall back award)
**Glasgow Caledonian University**	BSc Nursing Studies: Adult Nursing
**King's College London**	BSc Nursing with Registration as an Adult Nurse
**University of Ulster**	BSc(Hons) Nursing (Adult)
**University of Lincoln**	Degree Science Nursing (Registered Nurse - Adult: Apprenticeship Route)
**University of Salford**	MA Nursing (adult)
**University Of Wolverhampton**	Master of Adult Nursing
**University Of Wolverhampton**	Master of Nursing (Adult Nursing)
**University Of Birmingham**	Master of Nursing (Adult)
**University Of Wolverhampton**	Master of Nursing (MNurs) Adult Nursing
**University Of Birmingham**	Master of Science in Nursing (Adult)
**University of Derby**	Master of Science in Nursing (Adult)
**University of Derby**	Master of Science in Nursing (adult) (apprenticeship)
**University Of Manchester**	Masters in Nursing (Adult)
**University Of Edinburgh**	Masters in Nursing with pre-registration (adult)
**University Of Sheffield**	MMedSci Nursing (Adult)
**Queen Margaret University**	MN Nursing
**Edge Hill University**	MNSW Adult Nursing and Social Work
**University of Sunderland**	MNurse (Adult)
**University Of York**	MNursing (Adult)
**University Of Hertfordshire**	MSc Hons Nursing (Adult)

Approved education institution	Course
**University Of Northumbria At Newcastle**	MSc (Hons) nursing science (adult)
**University Of Dundee**	MSc Adult Nursing
**University Of Greenwich**	MSc Adult Nursing
**University of Chester**	MSc Adult Nursing
**University of Chester**	MSc Adult Nursing
**University of Chester**	MSc Adult Nursing
**University of Bedfordshire**	MSc Adult Nursing
**London South Bank University**	MSc adult nursing
**University of West of Scotland**	MSc Adult Nursing
**University of Chester**	MSc Adult Nursing
**Bournemouth University**	MSc Adult Nursing
**Kingston University & St George's University of London**	MSc Adult Nursing
**Coventry University**	MSc Adult Nursing
**University of Bedfordshire**	MSc Adult Nursing (apprenticeship)
**Birmingham City University**	MSc Adult Nursing (pre-registration)
**Manchester Metropolitan University**	MSc Adult Nursing (Pre-registration)
**University of Chester**	MSc Adult Nursing Apprenticeship
**Coventry University**	MSc Adult Nursing Blended Learning (pre-registration)
**Coventry University**	MSc Adult Nursing Blended Learning (pre-registration) Nursing Degree Apprenticeship
**University Of Southampton**	MSc Nursing (Adult)
**Canterbury Christ Church University**	MSc Nursing (Adult)
**City, University of London**	MSc Nursing (Adult)

Approved education institution	Course
**University Of Hertfordshire**	MSc Nursing (adult) - Nursing Degree Apprenticeship (part time route)
**University of Nottingham**	MSc nursing (graduate entry) (adult)
**University of Lincoln**	MSc Nursing (Pre-Registration – Adult)
**Edge Hill University**	MSc Nursing (Pre-registration Adult)
**University Of Huddersfield**	MSc Nursing (pre-registration) - Adult
**Swansea University**	MSc Nursing Pre-registration (Adult)
**Teesside University**	MSc Nursing Studies (Adult Apprenticeship)
**Glasgow Caledonian University**	MSc Nursing Studies: Adult Nursing
**University of Central Lancashire**	MSc nursing with registered nurse (adult)
**Liverpool John Moores University**	MSc Nursing with Registered Nurse Status (Adult)
**Liverpool John Moores University**	MSc Nursing with Registered Nurse Status (Adult)
**King's College London**	MSc Nursing with Registration as an Adult Nurse
**University Of Plymouth**	MSc Pre-registration Nursing (Adult Health)
**University Of Huddersfield**	MSc Pre-registration Nursing apprenticeship-Adult
**University Of Huddersfield**	MSc Pre-registration Nursing Apprenticeship Child (Blended Learning)
**University Of Huddersfield**	MSc Pre-registration Nursing Child (Blended Learning
**University of Cumbria**	MSc Pre-Registration Nursing/Registered Nurse: Adult Nursing
**Edge Hill University**	MSci Nurse Paramedic (Adult)
**University Of Exeter**	MSci nursing (adult)
**University of Leicester**	MSci Nursing with Leadership Adult Nursing

For peer review only

Approved education institution	Course
**Middlesex University**	Nurse Degree Apprenticeship Adult (4 year direct entry route)
**Middlesex University**	Nurse Degree Apprenticeship Adult (4 year part-time employed route)
**University of West London**	Nurse Degree Apprenticeship BNursing (Hons) Adult
**University of Central Lancashire**	Nursing Degree Apprenticeship route – Adult
**Teesside University**	Nursing Studies (Adult) (Pre-registration) BSc (Hons)
**Teesside University**	Nursing Studies (Adult) (Pre-Registration) MSc
**University of West of Scotland**	PG Dip Adult Nursing
**London South Bank University**	PG Dip adult nursing
**University Of Greenwich**	PG Dip Adult Nursing (fall back award)
**University of South Wales**	PG Dip Nursing Adult
**University of West London**	PG Diploma Nursing Adult
**Edge Hill University**	PgDip Nursing (second registration Adult)
**University Of Southampton**	Postgraduate Diploma Nursing (Adult)
**The Queen's University Of Belfast**	Pre-registration nursing - Adult
**Sheffield Hallam University**	Pre-registration nursing - Adult
**The Queen's University Of Belfast**	Pre-registration nursing - Adult (Graduate Entry)
**Staffordshire University**	Registered Nurse (Adult)
**University Of Brighton**	Registered Nurse Degree Apprenticeship (Adult)

UK M.S.C Approved Medicine and Surgery Programmes

Institution Name
University of Aberdeen School of Medicine and Dentistry
Anglia Ruskin University School of Medicine
Aston University Medical School
Barts and The London School of Medicine and Dentistry
University of Birmingham College of Medical and Dental Sciences
Brighton and Sussex Medical School
University of Bristol Medical School
University of Buckingham Medical School
University of Cambridge School of Clinical Medicine
Cardiff University School of Medicine
University of Dundee School of Medicine
Edge Hill University Medical School
The University of Edinburgh Medical School
University of Exeter Medical School
University of Glasgow School of Medicine
Hull York Medical School
Imperial College London Faculty of Medicine
Keele University School of Medicine
Kent and Medway Medical School
King's College London GKT School of Medical Education
Lancaster University Medical School
University of Leeds School of Medicine
University of Leicester Medical School
University of Liverpool School of Medicine
University of Manchester Medical School
Newcastle University School of Medical Education
Norwich Medical School
University of Nottingham School of Medicine

For peer review only

University of Nottingham - Lincoln Medical School
University of Oxford Medical Sciences Division
Plymouth University Peninsula Schools of Medicine and Dentistry
Queen's University Belfast School of Medicine
University of Sheffield Medical School
University of Southampton School of Medicine
University of St Andrews School of Medicine
St George's, University of London
University of Sunderland School of Medicine
Swansea University Medical School
University of Central Lancashire School of Medicine
University College London Medical School
University of Warwick Medical School

For peer review only

Patient Information Section

General information

The aim of this study is to determine the impact of the COVID-19 pandemic on the mental health of UK medical and nursing students, one year down the line from the initial outbreak. We are inviting all medical and nursing students enrolled at university in the UK to participate in this study. Please read the following section carefully before taking part. If you have any questions regarding the study please contact the research team via the following email: kate.saunders@psych.ox.ac.uk

What is the purpose of the study?

The purpose of SPICE-20 is to evaluate the continued impact of the COVID-19 pandemic on UK medical students, one year on from the SPICE-19 study. In addition, we aim to determine the effect on UK nursing students. In doing so we hope to provide feedback to UK medical schools and Nursing institutions about the current collective mental health of student healthcare professionals. We believe this information is vital to ensure adequate mental wellbeing service provision is in place at higher education institutions.

Why am I being invited to participate in this study?

You have been invited to take part in this study because we are recruiting medical and nursing students to participate and feedback on their continued mental wellbeing during the UK pandemic. You are eligible to participate if you are a current UK medical or nursing student in the UK and have access to the internet.

What happens if I choose to participate?

Participation in this study is entirely voluntary. To be involved you will be required to complete a short survey online. The survey will ask for general background details about you including: your current educational status, age, gender, ethnicity. Additionally, questions will ask about your current mood and mental wellbeing. Your decision to participate in this study will not have any academic impact and will be entirely anonymous. The survey is expected to take approximately 10-15 minutes to complete

For peer review only

however it may be done as quick or slow as you like. There is no prior knowledge required to participate. We will ask for your consent for collection and storage of data in accordance with General Data Protection Regulation (GDPR) before the survey begins. More information regarding the rules and regulations of GDPR can be found at www.gdpr-info.eu .

Do I have to participate?

As stated above, participation in the SPICE-20 study is entirely voluntary and anonymous. Consent for involvement may be withdrawn anytime prior to completion of the survey. To do so, simply close the tab or browser containing the survey. Following this, no personal data provided up to that point in the survey will be stored or used for research or any other purpose. Completion of all questions is entirely optional. This means that participation is still possible even if there are certain questions you would prefer not to answer. If there is a question you would like to omit from your responses, simply select the option “Prefer not to answer”. Any data collected will not be provided to your higher education institution without being anonymised, meaning your feedback will not make your identifiable.

Are there any risks of being involved?

Some of the questions included in the questionnaire may potentially cause some distress by brining up painful memories. If at any point during completion of the survey you are feeling distressed you may stop or choose not to answer troubling questions. Included with the study information is mental wellbeing support services that are available to access.

Are there any benefits to being involved?

The completion of this survey will not provide any academic benefit at your respective institutions. However, the results of SPICE-20 will be used to inform university wellbeing service provision. This means that by participating in the study you have the opportunity to provide anonymous feedback to your institution about what you feel is being done well, or could be done better to support students. Additionally, this survey may provide be useful as a personal opportunity for reflection.

For peer review only

How will my data be used?

Your answers will be completely anonymous and no identifiable data will be included. Data will be stored in a password protected file and will only be accessible by individuals on the steering/writing committee that have a reason to access it. IP addresses will not be stored. If you provide your email address, it will be deleted at the end of the study and not linked to any information you provide. Anonymised responses to the survey will be stored for a minimum of ten years after publication or public release.

Who will have access to your data?

Qualtrics is the data platform used for SPICE-20. They are the data controller in respect to the personal data they hold and as such they determine how personal data is used. Their privacy notice can be found at: www.qualtrics.com/privacy-statement. Qualtrics will share any email address you provide with the University of Oxford for the purposes of research only. Responsible members of the University of Oxford may be given access to your data for monitoring or audit of the study and ensuring that guidelines are complied with.

Where will the results be published?

The results of SPICE-20 may be published in a peer reviewed academic journal. In addition, the results of the study may be presented at conferences, either in oral or poster format. The study is targeting a completion date of September 2021. Following this provisional date a summary of the results and outcomes will be available on request to the study team.

Who do I contact if I have any queries or wish to make a complaint about the study?

Our study team aims to operate in a transparent manner. If you have any queries or complaints regarding the study aims, distribution, or any other aspect please contact the study team at kate.saunders@psych.ox.ac.uk and we will do our best to provide an adequate response. Our research team aims to reply within 10 working days. If you have any further issues or wish to escalate your inquiry please contact the Chair of the Medical Sciences Inter-Divisional Research Ethics Committee.

For peer review only

Email: ethics@medsci.ox.ac.uk. Address: Research Services, University of Oxford, Wellington Square, Oxford, OX1 2JD.

Who do I contact for further details?

For any queries or other details, contact the principle investigator Professor Kate Saunders at kate.saunders@psych.ox.ac.uk.

Please check the box to confirm you are the age of 18 years old or older

☐ I certify that I am 18 years or older.

If you have read the information above and agree to participate with the understanding that the data (including personal data) you submit will be processed accordingly, please check the relevant box to get started

☐ Yes, I agree to take part.

For peer review only

BMJ Open

Social and Psychological Impact of the Covid-19 pandemic on UK medical and nursing students: Protocol for a national medical and nursing student survey

Journal:	*BMJ Open*
Manuscript ID	bmjopen-2021-057467.R1
Article Type:	Protocol
Date Submitted by the Author:	03-Feb-2022
Complete List of Authors:	Richardson, George; University of Liverpool School of Medicine, Institute of Translational Medicine<br>Gillespie, Conor; University of Liverpool, Institute of Systems Molecular and Integrative Biology<br>Mantle, Orla; King's College London Faculty of Life Sciences and Medicine<br>Clynch, Abigail; University of Liverpool, Institute of Systems Molecular and Integrative Biology<br>Ooi, Setthasorn Zhi Yang; Cardiff University School of Medicine<br>Park, Jay; The University of Edinburgh Edinburgh Medical School<br>Bligh, Emily R.; The University of Sheffield, School of Medicine<br>Kundu, Shantanu; University of Liverpool, School of Medicine<br>Georgiou, Ioannis; University of Aberdeen, School of Medicine<br>Bandyopadhyay, Soham; University of Oxford, Oxford University Medical School<br>Saunders, Kate E ; University of Oxford, Department of Psychiatry; Oxford Health NHS Foundation Trust<br>Interest Group, Neurology and Neurosurgery; Neurology and Neurosurgery Interest Group<br>SPICE-20, Collaborative; SPICE-20 Collaborative
**Primary Subject Heading**:	Medical education and training
Secondary Subject Heading:	Medical education and training, Mental health
Keywords:	MENTAL HEALTH, MEDICAL EDUCATION & TRAINING, Child & adolescent psychiatry < PSYCHIATRY

SCHOLARONE™
Manuscripts

1

Social and Psychological Impact of the Covid-19 pandemic on UK medical and nursing students: Protocol for a national medical and nursing student survey

Authors and affiliations:
George E. Richardson*[1], Conor S. Gillespie*^[2], Orla Mantle[3], Abigail Clynch[2], Setthasorn Z. Y. Ooi[4], Jay Park[5], Emily R. Bligh[6], Shantanu Kundu[7], Ioannis Georgiou[8], Soham Bandyopadhyay[9], Kate E.A. Saunders[10,11], Neurology and Neurosurgery Interest Group (NANSIG), SPICE-20 Collaborative.

[1]Institute of Translational Medicine, University of Liverpool, Liverpool, UK
[2]Institute of Systems, Molecular and Integrative Biology, University of Liverpool, Liverpool, UK
[3]Faculty of Life Sciences and Medicine, King's College London, London, United Kingdom
[4]Cardiff University School of Medicine, University Hospital of Wales Main Building, Heath Park, Cardiff, UK
[5]Edinburgh Medical School, University of Edinburgh, Edinburgh, UK
[6]School of Medicine, University of Sheffield, Sheffield, UK
[7]School of Medicine, University of Liverpool, Liverpool, UK
[8]School of Medicine, University of Aberdeen, Aberdeen, UK
[9]Oxford University Global Surgery Group, Nuffield Department of Surgical Sciences, Medical Sciences Division, University of Oxford, Oxford, UK.
[10]Department of Psychiatry, Warneford Hospital, University of Oxford, Warneford Lane, Oxford, UK
[11]Oxford Health NHS Foundation Trust, Warneford Hospital, University of Oxford, Warneford Lane, Oxford, UK

*Joint first author, each author contributed equally to the work.

^Corresponding author

Abstract word count: 296

Full text word count: 2804

Corresponding author contact details:
Conor S. Gillespie
Institute of Systems, Molecular and Integrative Biology (ISMIB)
University of Liverpool
Biosciences Building
Crown Street
Liverpool
L69 7BE
Email address: hlcgill2@liv.ac.uk

For peer review only

Keywords: Wellbeing, Mental Health, Survey, Medical Students

ABSTRACT

Introduction

Healthcare students have played a significant role in the NHS during the COVID-19 pandemic. We captured data on the wellbeing of medical students during the acute phase of the pandemic with the Social and Psychological Impact of COVID-19 on medical students: a national survey Evaluation (SPICE-19) study. We will evaluate changes in mental health and wellbeing of medical and nursing students one year after SPICE-19, in a cross-sectional study, to understand the impact of the pandemic, and inform wellbeing policies.

Methods and Analysis

This study will be a national, multi-institution, cross-discipline study. An online, 53-item survey of demographics, mental health and wellbeing will be used to record responses. Students studying for a medical or nursing degree at any UK universities will be eligible to participate. The survey will be advertised through the Neurology and Neurosurgery Interest Group (NANSIG) national network. Participation is anonymous and voluntary, with relevant mental health resources made available to participants.

Ethics and Dissemination

Ethical approval was granted by the University of Oxford Central University Research Ethics Committee (R75719/RE001) on 21st May 2021. Study findings will be presented at national and international meetings, and submitted for publication in a peer-reviewed journal.

Strengths and limitations

- This is a multi-centre, national questionnaire-based survey of medical and nursing student mental health and wellbeing in the UK.
- This is the first study to assess mental health and wellbeing of the student multi-disciplinary team during the new normal after the Covid-19 pandemic.

For peer review only - http://bmjopen.bmj.com/site/about/guidelines.xhtml

- The study results will provide valuable insight into healthcare workers, which can be used to identify and target further areas of wellbeing support and inform policy.

- Response bias may overestimate participation of students based on age, sex, and medical/nursing school, with those interested in neuroscience and mental health more likely to participate.

INTRODUCTION

Medical and nursing students in the UK have been significantly affected by the COVID-19 pandemic. During the first wave, many were active components of the clinical team, either by undertaking extending clinical placements, paid work in the NHS, or through voluntary roles, while others saw placements reduced, suspended, or cancelled completely.(1, 2) We evaluated medical student wellbeing and mental health during the acute phase of the pandemic with the Social and Psychological Impact of COVID-19 on medical students: a national service evaluation (SPICE-19) study,(3) a cross sectional study with a prospective component, which identified several points for service provision improvement based on 2275 responses across 34 medical schools in the UK.(4-6)

It is pertinent to re-evaluate the mental health of medical students, one year removed from SPICE-19, to explore how they are navigating their training, education, and the impact of adapting to the 'new normal' of medical education provision.(7, 8) It is also important to elucidate the effect of COVID-19 on the wellbeing and mental health of UK nursing students, many of whom were asked to volunteer on the front line in COVID wards,(2) and a large-scale study examining this has not been completed. A single centre survey in the US noted that nursing students reported increased levels of anxiety, reduction in concentration, and difficulties with academic workload throughout the pandemic,(9) and a large multi-institutional study is required to elucidate this further. This is in accordance with previously published work by the SPICE-19 research team. (10, 11)

Medical and nursing schools have each responded differently to the pandemic, with the accessibility of wellbeing and mental health services variable between universities.(12) A national survey assessing mental health and wellbeing in medical and nursing students, as well as support systems available for those facing mental health difficulty is required. Evaluating the mental health and wellbeing of the student multi-disciplinary team is vital for

For peer review only

ensuring adequate service provision is provided - it is paramount to safeguard the wellbeing of the next generation of healthcare professionals.(13, 14)

The primary aim of this study is to comprehensively evaluate mental health and wellbeing of medical and nursing students during the evolving COVID-19 pandemic, one year after the initial outbreak. The secondary aims are to elucidate students' perceptions of institutional efforts to provide support, and accessibility and appropriateness of such support. The study logos are shown in Figure 1.

Figure 1. SPICE-20 study logos.

METHODS AND ANALYSIS

Study partners

NANSIG

The Neurology and Neurosurgery Interest Group (NANSIG) was formed in 2010. It is a student and junior doctor led interest group designed to increase participation, diversity and engagement in Neurosciences, and has over 1000 members internationally.(15) The organisation has an affiliation with the Society of British Neurosurgeons (SBNS) and the Association of British Neurologists (ABN) (www.nansig.org). The organisation led the SPICE-19 national cohort study last year, assessing wellbeing in 2275 medical students in response to the pandemic, and is familiar with survey design and distribution. The collective organisation runs established national and international events, and has published over 20 peer-reviewed publications.

Study design

SPICE-20 is a national, multi-institution, cross-sectional study. An online survey will be used to record participant responses. The survey contains 53 items - section 1 of the survey includes background and demographic information. Section 2 assesses wellbeing by asking participants to complete the Warwick-Edinburgh Mental Well Being Scale (WEMWBS); a 14-point, validated evaluation of wellbeing.(16) Section 3 encompasses the Patient Health Questionnaire-9 (PHQ-9) and Generalised Anxiety Disorder-7 (GAD-7). The PHQ-9 is a valid diagnostic and severity measure for depressive disorder,(17) and GAD-7 is a seven-item,

For peer review only

validated assessment of generalised anxiety.(18, 19) Section 4 includes questions about university support offered by each institution, if participants had accessed this support, and any significant changes to teaching encountered during the pandemic. Section 4 contains study specific unvalidated questions. These questions were defined for the current study to meet the specific secondary aims and were not adapted from the SPICE-19 survey, due to a difference in study objectives.

The survey was iteratively defined by the student-led study management team until a consensus was reached. Based on our experience with SPICE-19 survey, we have reduced the number of questions and limited the survey to validated measures of wellbeing and mental health. The final, 53-item questionnaire is shown in Figure 2.

Figure 2: Final SPICE-20 survey design.

Survey piloting

The survey was piloted by a group of 15 medical students from the NANSIG core committee. These students were not involved in the design of the study and were consulted in order to provide feedback, improve clarity and ensure objectivity. Data was analysed to identify any points of concern, estimated completion time, and difficult survey items/questions. Students were contacted to identify any suggestions for improvement, with none identified. Therefore, no further alterations were made.

Survey administration

The survey was hosted on the Qualtrics™ survey platform (Provo, Utah, USA), a GDPR-compliant online survey platform, that facilitates both mobile and desktop devices.

Study dissemination

To maximise distribution across the UK, a national network of SPICE-20 Collaborative members were recruited, representing all 33 medical schools in the UK, using a purposive sampling method. Most held concurrent committee or regional lead roles in NANSIG at time of recruitment. Each member was asked to acquire the most up to date (as of April 2021)

For peer review only

available resources, guidance and support policies in place for their current medical school, plus an additional nursing school. A standard advertising method was developed by the steering committee and based on the successful recruitment strategy employed in the SPICE-19 study, which involved members contacting their respective medical school deans to request distribution of the survey at inception, as well as regular advertising over the 8-week study collection period, from 7st June 2021 to the 7rd August 2021. The distribution links for the survey includes university mailing lists, student society pages, and social media platforms. Figure 3 provides an example of the email template used to disseminate the survey through student mailing lists. Adverts will also be placed on NANSIG social media platforms, as well as the monthly NANSIG newsletter.

In the invitation to complete the study, based on their medical/nursing school, participants will also receive wellbeing, mental health, and guidance services specific to their institution, should they need to access them at any point. Participants will also be made aware of our patient and public involvement organisation, and their contact details. No incentives for participation will be offered.

Figure 3. Standard email template used for all survey dissemination through student email lists, developed by the study team and approved by the research ethics committee.

Eligibility and representation

All current students enrolled at UK medical schools recognised by the General Medical Council (GMC) and the Medical Schools Council (MSC) will be eligible to participate. Additionally, all current home and international students enrolled on an adult, child and mental health nursing degree at a UK university are eligible for inclusion. A list of eligible medical schools, and approved programmes by the Nursing and Midwifery Council (NMC) is included in the supplementary materials (document “Supplementary Material – MSC and NMC”).

Consent and confidentiality

A patient information sheet will be shown on the first page of the study (supplementary material - patient information). This encompasses the rationale, purpose, and voluntary nature of participation. Participants must verify that they are over 18 years old and provide

informed consent via tick box. It will be emphasised that participation is anonymous, confidential, voluntary, and that participants can withdraw consent at any time.

Data security

All data will be collected and stored on the secure online server, Qualtrics™. Survey data will be extracted from the software to a password-protected, Microsoft Excel spreadsheet (Microsoft, California, USA) only available to the study team. Data handling and record keeping will adhere to the University of Oxford standards for data security.

Statistical analysis

Data will be analysed using R version 4.0.3. The overall wellbeing of medical and nursing students, as assessed using the WEMWBS, and mental health, using the PHQ-9 and GAD-7, will be presented using descriptive statistics. The scores of WEMWBS, PHQ-9 and GAD-7 will be categorised using established cut off points into dichotomous groups, corresponding to individuals expressing symptoms and those which do not. (20-22) No information that identifies or is specific to institutions will be presented. Appropriate statistical methods will be selected based on the distribution and type of data; it is anticipated data will be non-parametric, and, will therefore be compared using tests such as $\chi 2$ or Kruskal-Wallis tests. Differences between medical and nursing students will be compared using these statistical tests. To identify factors associated with an increased scores on WEMWBS, PHQ-9, and GAD-7, multi-variate generalised linear regression will be used.

Sample size calculation

Approximate estimates of total UK medical and nursing students were calculated from yearly undergraduate intake figures. The total number of UK medical and adult nursing students was estimated to be 84,000. An online calculator was used to determine the sample size needed for the survey. The minimum sample size required from our target population for a margin error of 5% and a confidence interval level of 95% is 383, assuming a 50% response distribution for each question, the most conservative response distribution for study power.

Patient and public involvement

For peer review only

As the study population was medical and nursing student wellbeing and mental health, we worked collaboratively with the Be Free Campaign - a mental health and wellbeing awareness charity, from study inception. Be Free were involved in the conception, design and development of the study. The charity works with young people and students, emphasising mental health and wellbeing, to tackle the stigma behind mental health, and promote expression of individual values (UK registered charity number 1189704). The charity director and clinical study team for the charity comprehensively reviewed the protocol for appropriateness of content, question format, and ensured the questions asked were sensitive and pertinent to medical and nursing students.

Be Free have extensive knowledge of indicators of mental health, and the questions selected (WEMWBS, PHQ-9 and GAD-7) were done so in concordance with their advice. The number of questions, layout, and design were also prepared in accordance with their suggestions. The charity inspired the conception of this study and approved the final survey design. The charity founder and director (SK) is also a member of the steering committee for this study.

DISCUSSION

Study rationale

International multi-institutional studies have demonstrated that during the initial COVID-19 outbreak, medical students were less likely to experience deterioration in mental health and wellbeing when compared to non-medical students. (23, 24) This is the first national, multi-institutional study to evaluate the mental health of UK medical and nursing students during the COVID-19 pandemic.(25) It will therefore be possible to elucidate the mental health and wellbeing of healthcare students across the UK. By comparing medical and nursing schools, it will be possible to evaluate the nature of successful support, as well as students' confidence and ability to access this support. The findings will inform service provision, and highlight areas where improvement is required. The survey will also highlight local and national wellbeing resources available to students.

Why assess medical and nursing student wellbeing and mental health?

For peer review only

Medical and nursing students form the healthcare multi-disciplinary team of the future. Mental health and wellbeing are positively correlated with job satisfaction, retention, and performance, and is an essential component of good health.(26) If the NHS is to retain, recruit and develop its workforce of the future, ascertaining their mental health at this point in the COVID-19 pandemic is essential. Young people have been disproportionately affected by the pandemic, and most medical and nursing students fall into this age category. As non-emergency care is delayed and rescheduled, students will have to deal with the long acting repercussions of the pandemic, including those directly affected by the virus itself, including long-COVID,(27) declining mental health in the population exacerbated by the pandemic,(28) and implications of delayed access to healthcare.(29, 30)

Many medical students may not be aware that such support exists, either at their respective institution, or at a regional/national level, and we would do these students a disservice by not highlighting existing mental health and wellbeing services.

<u>Benefits of a national medical student wellbeing survey</u>

Medical and nursing schools across the UK adopt heterogenous practices to achieve the outcomes set in the GMC and NMC core competencies and curricula.(31) A survey of both medical and nursing students on a national scale is the best way to evaluate mental health and wellbeing. Many institutions reacted differently to the pandemic, and a survey is best placed to simultaneously decipher wellbeing and mental health at each institution, while assessing the impact of the pandemic on a national scale, and highlighting heterogeneity between institutions. Furthermore, there is a need to appreciate that multi-institutional studies of large magnitude are required to answer important clinical questions and elucidate concerns not identified by smaller studies. Multi-centre studies are also less susceptible to identity issues because the data is aggregated between multiple medical schools, and can be presented as all centres.

<u>Limitations</u>

Online surveys have the advantage of ubiquitous presence, and increased accessibility to medical and nursing schools in the UK. However, several limitations can arise from such a study method.

For peer review only

Firstly, those with an existing interest in wellbeing and mental health may be more likely to engage with and complete the survey. This could lead to selection bias towards those adversely affected by the pandemic. The anticipated magnitude of this effect is unclear. In addition, as the survey is being distributed through NANSIG, there may be over-participation from students with an interest in Neurosciences and their surrounding connections.

In order to ameliorate this as best as possible, we have adopted a varied dissemination approach. This includes having representatives at each UK medical school, survey distribution to medical and nursing school bulletins, and newsletters. We hope this will improve visibility and participation. We decided not to incentivise participants with a potential prize for completing the survey, as we wanted responses to be motivated by a desire to complete the questions in as open a way as possible.

The study is also limited by its cross-sectional design, and will therefore only provide a snapshot of mental health and wellbeing at the time of survey completion. In addition, during capricious times such as COVID, fluctuations in mental health may occur in accordance with policy changes, as well as institutional policies towards placements and assessment, and we will assess date of study completion as a potential confounder as a variable in our analysis.

The self-reporting of symptoms, will also be a significant limitation, in addition to the fact that the questions included are screening tools for mental health and wellbeing, and are not immediately diagnostic. Furthermore, a number of important variables including socio-economic status, co-morbid psychiatric conditions, and levels of isolation, have not been included within the survey. Omission of these and other variables may cause confounding of study results. The decision not to include these variables was made to ensure minimal barriers existed to completion of the survey, however we recognise that the results must be interpreted within the context of this limitation. Finally, there are no imbedded security measures to confirm a respondents medical or nursing school, and therefore we cannot guarantee that all participants were UK medical or nursing students.

Ethics and dissemination

Ethical approval for this study was granted by the Oxford Research ethics committee [R75719/RE001]. The findings of the SPICE-20 study as described in this protocol, will be presented at scientific conferences, and submitted for publication in a peer-review journal once finalised.

For peer review only - http://bmjopen.bmj.com/site/about/guidelines.xhtml

Acknowledgments

The authors would like to thank the SPICE-19 collaborative, whose work inspired this study, and are grateful to the Be Free Campaign for their assistance with the conception, design and inspiration for components of the study. We also thank NANSIG and members of the SPICE-20 collaborative, for their kind assistance disseminating the survey.

Funding statement

No external funding has been sought in the conception, design or implementation of this study. CSG is a recipient of a grant from the Wolfson Foundation. GER is the recipient of funding from North West Cancer Research. KS is supported by the NIHR Oxford Health Biomedical Research Centre. The views expressed are those of the author(s) and not necessarily those of the NHS, the NIHR or the Department of Health.

Competing interests' statement

None to declare.

Presented at: N.A.

Author Contributions

GER, CSG, and OM were responsible for initial drafting of protocol manuscript, conceptualisation and designing the study. AC, SZYO, JP, ERB, IG, SB, and KS formed the wider study group and reviewed and approved drafts of the manuscript. KS provided supervision of the project. SK reviewed the manuscript and also provided PPI involvement and approval for the study.

Data Sharing Statement

Data are available upon reasonable request. All data must be anonymised entirely prior to any sharing request completion.

For peer review only

12

REFERENCES

1. Miller DG, Pierson L, Doernberg S. The Role of Medical Students During the COVID-19 Pandemic. Annals of Internal Medicine. 2020;173(2):145-6.
2. Swift A, Banks L, Baleswaran A, Cooke N, Little C, McGrath L, et al. COVID-19 and student nurses: A view from England. Journal of Clinical Nursing. 2020;29(17-18):3111-4.
3. Soham B, Ioannis G, Bibire B, Conor SG, Marta de Andres C, Mohammad Talha B, et al. Research Square. 2021.
4. Coyle C, Ghazi H, Georgiou I. The mental health and well-being benefits of exercise during the COVID-19 pandemic: a cross-sectional study of medical students and newly qualified doctors in the UK. Irish Journal of Medical Science (1971 -). 2020.
5. Norton EJ, Georgiou I, Fung A, Nazari A, Bandyopadhyay S, Saunders KEA. Personal protective equipment and infection prevention and control: a national survey of UK medical students and interim foundation doctors during the COVID-19 pandemic. Journal of Public Health. 2020.
6. Georgiou I, Hounat A, Park JJ, Gillespie C, Bandyopadhyay S, Saunders KEA. The Factors That Influenced Medical Students' Decision to Work Within the NHS During the COVID-19 Pandemic-A National, Cross-sectional Study. J Occup Environ Med. 2021;63(4):296-301.
7. Lucey CR, Johnston SC. The Transformational Effects of COVID-19 on Medical Education. JAMA. 2020;324(11):1033-4.
8. Yuen J, Xie F. Medical education during the COVID-19 pandemic: perspectives from UK trainees. Postgraduate Medical Journal. 2020;96(1137):432.
9. Fitzgerald A, Konrad S. Transition in learning during COVID-19: Student nurse anxiety, stress, and resource support. Nurs Forum. 2021:10.1111/nuf.12547.
10. Coyle C, Ghazi H, Georgiou I. The mental health and well-being benefits of exercise during the COVID-19 pandemic: a cross-sectional study of medical students and newly qualified doctors in the UK. Ir J Med Sci. 2020:1-2.
11. Norton EJ, Georgiou I, Fung A, Nazari A, Bandyopadhyay S, Saunders KEA. Personal protective equipment and infection prevention and control: a national survey of UK medical students and interim foundation doctors during the COVID-19 pandemic. J Public Health (Oxf). 2021;43(1):67-75.
12. Arora A, Solomou G, Bandyopadhyay S, Simons J, Osborne A, Georgiou I, et al. Adjusting to Disrupted Assessments, Placements and Teaching (ADAPT): a snapshot of the early response by UK medical schools to COVID-19. medRxiv. 2020:2020.07.29.20163907.
13. Kinder F, Harvey A. Covid-19: the medical students responding to the pandemic. BMJ. 2020;369:m2160.
14. Karaca A, Yildirim N, Cangur S, Acikgoz F, Akkus D. Relationship between mental health of nursing students and coping, self-esteem and social support. Nurse Educ Today. 2019;76:44-50.
15. Gillespie CS, Bandyopadhyay S, Jenkins AJ, Hutchinson P. Inspiring the next generation. The Lancet Neurology. 2021;20(4):256-7.
16. Tennant R, Hiller L, Fishwick R, Platt S, Joseph S, Weich S, et al. The Warwick-Edinburgh Mental Well-being Scale (WEMWBS): development and UK validation. Health Qual Life Outcomes. 2007;5:63.
17. Kroenke K, Strine TW, Spitzer RL, Williams JB, Berry JT, Mokdad AH. The PHQ-8 as a measure of current depression in the general population. J Affect Disord. 2009;114(1-3):163-73.
18. Spitzer RL, Kroenke K, Williams JBW, Löwe B. A Brief Measure for Assessing Generalized Anxiety Disorder: The GAD-7. Archives of Internal Medicine. 2006;166(10):1092-7.

19. Löwe B, Decker O, Müller S, Brähler E, Schellberg D, Herzog W, et al. Validation and standardization of the Generalized Anxiety Disorder Screener (GAD-7) in the general population. Med Care. 2008;46(3):266-74.
20. Johnson SU, Ulvenes PG, Øktedalen T, Hoffart A. Psychometric Properties of the General Anxiety Disorder 7-Item (GAD-7) Scale in a Heterogeneous Psychiatric Sample. Front Psychol. 2019;10:1713-.
21. Levis B, Benedetti A, Thombs BD. Accuracy of Patient Health Questionnaire-9 (PHQ-9) for screening to detect major depression: individual participant data meta-analysis. BMJ. 2019;365:l1476.
22. School WM. Collect, Score, Analyse and Interpret WEMWBS 2022 [Available from: https://warwick.ac.uk/fac/sci/med/research/platform/wemwbs/using/howto/.
23. Xie L, Luo H, Li M, Ge W, Xing B, Miao Q. The immediate psychological effects of Coronavirus Disease 2019 on medical and non-medical students in China. Int J Public Health. 2020;65(8):1445-53.
24. Leroy A, Wathelet M, Fovet T, Habran E, Granon B, Martignène N, et al. Mental health among medical, healthcare, and other university students during the first COVID-19 lockdown in France. J Affect Disord Rep. 2021;6:100260-.
25. Lyons Z, Wilcox H, Leung L, Dearsley O. COVID-19 and the mental well-being of Australian medical students: impact, concerns and coping strategies used. Australasian Psychiatry. 2020;28(6):649-52.
26. Spoorthy MS, Pratapa SK, Mahant S. Mental health problems faced by healthcare workers due to the COVID-19 pandemic-A review. Asian J Psychiatr. 2020;51:102119-.
27. Mandal S, Barnett J, Brill SE, Brown JS, Denneny EK, Hare SS, et al. 'Long-COVID': a cross-sectional study of persisting symptoms, biomarker and imaging abnormalities following hospitalisation for COVID-19. Thorax. 2020:thoraxjnl-2020-215818.
28. Xiong J, Lipsitz O, Nasri F, Lui LMW, Gill H, Phan L, et al. Impact of COVID-19 pandemic on mental health in the general population: A systematic review. J Affect Disord. 2020;277:55-64.
29. Lazzerini M, Barbi E, Apicella A, Marchetti F, Cardinale F, Trobia G. Delayed access or provision of care in Italy resulting from fear of COVID-19. Lancet Child Adolesc Health. 2020;4(5):e10-e1.
30. Czeisler M, Marynak K, Clarke KEN, Salah Z, Shakya I, Thierry JM, et al. Delay or Avoidance of Medical Care Because of COVID-19-Related Concerns - United States, June 2020. MMWR Morb Mortal Wkly Rep. 2020;69(36):1250-7.
31. McManus IC, Harborne AC, Horsfall HL, Joseph T, Smith DT, Marshall-Andon T, et al. Exploring UK medical school differences: the MedDifs study of selection, teaching, student and F1 perceptions, postgraduate outcomes and fitness to practise. BMC Med. 2020;18(1):136.

For peer review only

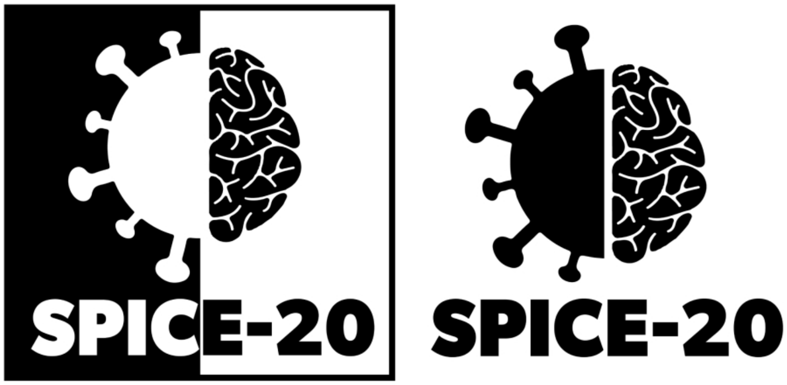

Figure 1. SPICE-20 study logos.

533x755mm (118 x 118 DPI)

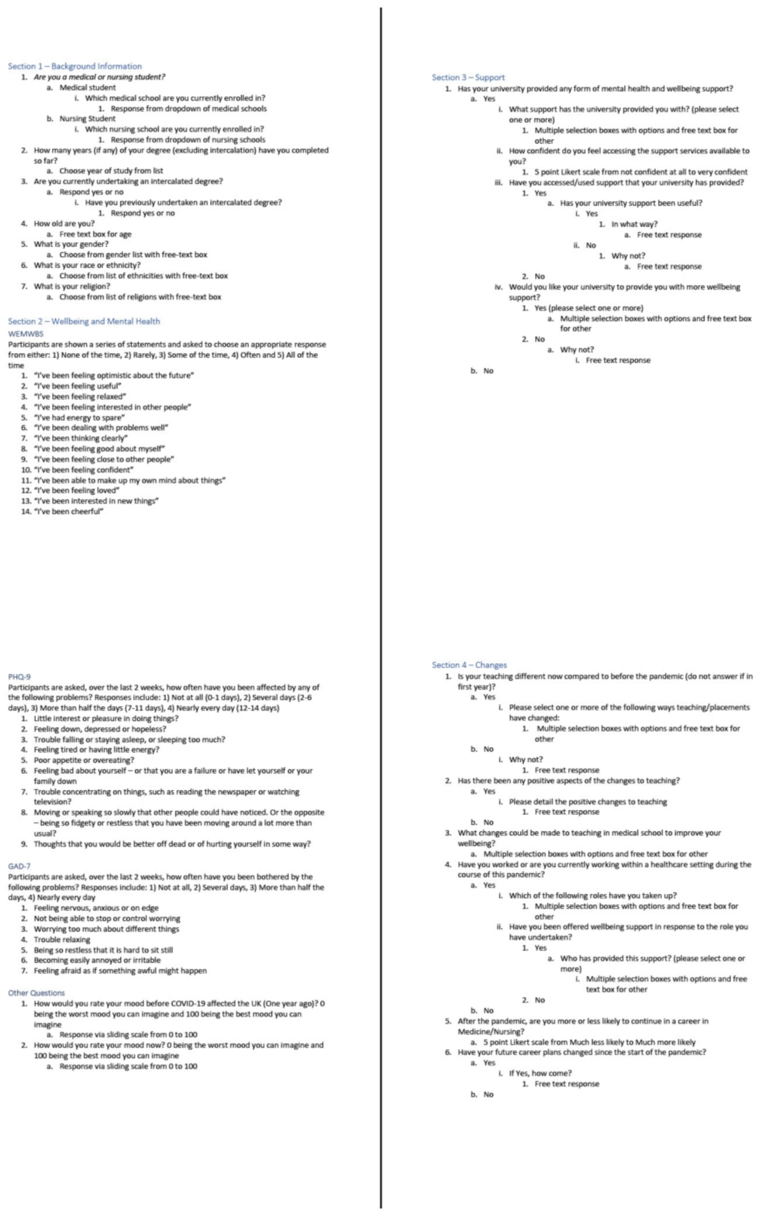

Section 1 – Background Information

1. *Are you a medical or nursing student?*
    a. Medical student
        i. Which medical school are you currently enrolled in?
            1. Response from dropdown of medical schools
    b. Nursing Student
        i. Which nursing school are you currently enrolled in?
            1. Response from dropdown of nursing schools
2. How many years (if any) of your degree (excluding intercalation) have you completed so far?
    a. Choose year of study from list
3. Are you currently undertaking an intercalated degree?
    a. Respond yes or no
        i. Have you previously undertaken an intercalated degree?
            1. Respond yes or no
4. How old are you?
    a. Free text box for age
5. What is your gender?
    a. Choose from gender list with free-text box
6. What is your race or ethnicity?
    a. Choose from list of ethnicities with free-text box
7. What is your religion?
    a. Choose from list of religions with free-text box

Section 2 – Wellbeing and Mental Health

WEMWBS

Participants are shown a series of statements and asked to choose an appropriate response from either: 1) None of the time, 2) Rarely, 3) Some of the time, 4) Often and 5) All of the time

1. "I've been feeling optimistic about the future"
2. "I've been feeling useful"
3. "I've been feeling relaxed"
4. "I've been feeling interested in other people"
5. "I've had energy to spare"
6. "I've been dealing with problems well"
7. "I've been thinking clearly"
8. "I've been feeling good about myself"
9. "I've been feeling close to other people"
10. "I've been feeling confident"
11. "I've been able to make up my own mind about things"
12. "I've been feeling loved"
13. "I've been interested in new things"
14. "I've been cheerful"

PHQ-9

Participants are asked, over the last 2 weeks, how often have you been affected by any of the following problems? Responses include: 1) Not at all (0-1 days), 2) Several days (2-6 days), 3) More than half the days (7-11 days), 4) Nearly every day (12-14 days)

1. Little interest or pleasure in doing things?
2. Feeling down, depressed or hopeless?
3. Trouble falling or staying asleep, or sleeping too much?
4. Feeling tired or having little energy?
5. Poor appetite or overeating?
6. Feeling bad about yourself – or that you are a failure or have let yourself or your family down
7. Trouble concentrating on things, such as reading the newspaper or watching television?
8. Moving or speaking so slowly that other people could have noticed. Or the opposite – being so fidgety or restless that you have been moving around a lot more than usual?
9. Thoughts that you would be better off dead or of hurting yourself in some way?

GAD-7

Participants are asked, over the last 2 weeks, how often have you been bothered by the following problems? Responses include: 1) Not at all, 2) Several days, 3) More than half the days, 4) Nearly every day

1. Feeling nervous, anxious or on edge
2. Not being able to stop or control worrying
3. Worrying too much about different things
4. Trouble relaxing
5. Being so restless that it is hard to sit still
6. Becoming easily annoyed or irritable
7. Feeling afraid as if something awful might happen

Other Questions

1. How would you rate your mood before COVID-19 affected the UK (One year ago)? 0 being the worst mood you can imagine and 100 being the best mood you can imagine
    a. Response via sliding scale from 0 to 100
2. How would you rate your mood now? 0 being the worst mood you can imagine and 100 being the best mood you can imagine
    a. Response via sliding scale from 0 to 100

Section 3 – Support

1. Has your university provided any form of mental health and wellbeing support?
    a. Yes
        i. What support has the university provided you with? (please select one or more)
            1. Multiple selection boxes with options and free text box for other
        ii. How confident do you feel accessing the support services available to you?
            1. 5 point Likert scale from not confident at all to very confident
        iii. Have you accessed/used support that your university has provided?
            1. Yes
                a. Has your university support been useful?
                    i. Yes
                        1. In what way?
                            a. Free text response
                    ii. No
                        1. Why not?
                            a. Free text response
            2. No
        iv. Would you like your university to provide you with more wellbeing support?
            1. Yes (please select one or more)
                a. Multiple selection boxes with options and free text box for other
            2. No
                a. Why not?
                    i. Free text response
    b. No

Section 4 – Changes

1. Is your teaching different now compared to before the pandemic (do not answer if in first year)?
    a. Yes
        i. Please select one or more of the following ways teaching/placements have changed:
            1. Multiple selection boxes with options and free text box for other
    b. No
        i. Why not?
            1. Free text response
2. Has there been any positive aspects of the changes to teaching?
    a. Yes
        i. Please detail the positive changes to teaching
            1. Free text response
    b. No
3. What changes could be made to teaching in medical school to improve your wellbeing?
    a. Multiple selection boxes with options and free text box for other
4. Have you worked or are you currently working within a healthcare setting during the course of this pandemic?
    a. Yes
        i. Which of the following roles have you taken up?
            1. Multiple selection boxes with options and free text box for other
        ii. Have you been offered wellbeing support in response to the role you have undertaken?
            1. Yes
                a. Who has provided this support? (please select one or more)
                    i. Multiple selection boxes with options and free text box for other
            2. No
    b. No
5. After the pandemic, are you more or less likely to continue in a career in Medicine/Nursing?
    a. 5 point Likert scale from Much less likely to Much more likely
6. Have your future career plans changed since the start of the pandemic?
    a. Yes
        i. If Yes, how come?
            1. Free text response
    b. No

Figure 2: Final SPICE-20 survey design.

913x1291mm (118 x 118 DPI)

Dear Students,

We are a group of student researchers that are interested in investigating the current state of medical student mental health and wellbeing, one year on from the onset of the pandemic. We are inviting all UK medical students, from years 1 to 5 (and intercalating) to complete a short 15 minute survey to help us better understand how you are doing. Whilst completing the survey, you will be asked a variety of questions about your mental health and wellbeing, and the support that you have received. Your IP address will not be recorded and responses will be anonymous. There will not be any follow up survey. Useful links to support services have been provided along with the post-survey debrief. Additional information about the risks versus benefits of taking part in the survey can be found by following the study link below. **Please access the survey by the link below:**

https://psychiatryoxford.qualtrics.com/jfe/form/SV_eQmCH0FLewvbcWO

If you have any questions about the study or your involvement please contact a member of the study team at the following email and we will aim to reply within 24hrs: hlgricha@liverpool.ac.uk

Should you have concerns about your wellbeing do consider contacting you GP or accessing the resources available: <LOCAL SERVICE PROVISION INSERTED HERE>

Many thanks for taking the time to read this,

George Richardson
SPICE-20 Study Team

Figure 3. Standard email template used for all survey dissemination through student email lists, developed by the study team and approved by the research ethics committee.

554x668mm (57 x 57 DPI)

UK N.M.C Approved Adult Nursing Programmes

Approved education institution	Course
**University Of Essex**	Adult Nursing
**University Of Essex**	Adult Nursing (nursing degree apprenticeship route)
**Edinburgh Napier University**	Bachelor of Nursing (Adult)
**University Of Wolverhampton**	Bachelor of Nursing (BNurs (Hons)) Adult Nursing
**University Of Wolverhampton**	Bachelor of Nursing (BNurs (Hons)) Adult Nursing [Apprenticeship]
**University Of Glasgow**	Bachelor of Nursing (Honours)
**University Of Manchester**	Bachelor of Nursing (Hons) (Adult)
**University Of Birmingham**	Bachelor of Nursing (Hons) (Adult)
**University Of Liverpool**	Bachelor of Nursing (Hons) Nursing (Adult)
**University Of Glasgow**	Bachelor of Nursing (ordinary) [fall back award]
**Cardiff University**	Bachelor of Nursing Adult
**University of Derby**	Bachelor of Science (Honours) in Nursing (Adult)
**University of Derby**	Bachelor of Science (Honours) in Nursing (adult) (apprenticeship)
**University Of Sheffield**	BMed Sci (Hons) Nursing (Adult)
**University Of Sheffield**	BMedSci (Hons) Nursing (Adult) Apprenticeship route
**University of Chester**	BN (Hons) Adult Nursing
**University of Chester**	BN (Hons) Adult Nursing
**University of Chester**	BN (Hons) Adult Nursing
**University of Chester**	BN (Hons) Adult Nursing
**University of Chester**	BN (Hons) Adult Nursing

Approved education institution	Course
**University of Chester**	BN (Hons) Adult Nursing Apprenticeship
**University of Chester**	BN (Hons) Adult Nursing Apprenticeship
**University Of Portsmouth**	BN (Hons) Nursing (Adult)
**University of Winchester**	BN (Hons) Nursing (Adult)
**Glyndwr University (formerly North East Wales Inst. of HE)**	BN Nursing
**University Of Edinburgh**	BN nursing (adult)
**University Of Edinburgh**	BN nursing with Honours (adult)
**University of Chester**	BNurs (Hons) Adult
**University of Chester**	BNurs (Hons) Adult (part-time)
**University of Chester**	BNurs (Hons) Adult Apprenticeship
**University Of Wolverhampton**	BNurs (Hons) Adult Nursing
**University Of Wolverhampton**	BNurs (Hons) Adult Nursing Apprenticeship
**University of West London**	BNursing (Hons) Adult
**University Of Hertfordshire**	BSc Hons Nursing (Adult)
**University Of Stirling**	BSc (Honours) Nursing (Adult)
**Coventry University**	BSc (Hons) Adult Nursing
**Coventry University**	BSc (Hons) Adult Nursing (Degree apprenticeship)
**Oxford Brookes University**	BSc (Hons) (Adult)
**London South Bank University**	BSc (Hons) adult nursing
**University of Suffolk**	BSc (Hons) Adult Nursing
**University of West of Scotland**	BSc (Hons) Adult Nursing
**Leeds Beckett University**	BSc (Hons) Adult Nursing
**University of Northampton**	BSc (Hons) Adult Nursing
**Bournemouth University**	BSc (Hons) Adult Nursing

For peer review only

Approved education institution	Course
**University of Roehampton**	BSc (Hons) Adult Nursing
**University Of Dundee**	BSc (Hons) Adult Nursing
**University of West of Scotland**	BSc (Hons) Adult Nursing
**University Of Greenwich**	BSc (Hons) Adult Nursing
**University of Bedfordshire**	BSc (Hons) Adult Nursing
**Bournemouth University**	BSc (Hons) Adult Nursing (Apprenticeship)
**University of Bedfordshire**	BSc (Hons) Adult Nursing (apprenticeship)
**University of Sunderland**	BSc (Hons) Adult Nursing (blended)
**University of Sunderland**	BSc (Hons) Adult Nursing (blended)
**Leeds Beckett University**	BSc (Hons) Adult Nursing (degree apprenticeship)
**Manchester Metropolitan University**	BSc (Hons) Adult Nursing (Pre-registration)
**University of Suffolk**	BSc (Hons) Adult Nursing [degree apprenticeship]
**Coventry University**	BSc (Hons) Adult Nursing Blended Learning
**Coventry University**	BSc (Hons) Adult Nursing Blended Learning Nursing Degree Apprenticeship
**University Of Greenwich**	BSc (Hons) Adult Nursing Degree Apprenticeship
**Solent University**	BSc (Hons) Adult Nursing Practice – apprenticeship
**University of Sunderland**	BSc (Hons) adult nursing practice
**Solent University**	BSc (Hons) Adult Nursing Practice
**University Of Huddersfield**	BSc (Hons) Degree Apprenticeship - Registered Nurse Child (Blended Learning)
**University Of Huddersfield**	BSc (Hons) Degree Apprenticeship-Registered Nurse: Adult

For peer review only

Approved education institution	Course
**BPP**	BSc (Hons) Nursing (Adult) [Nursing Degree Apprenticeship]
**BPP**	BSc (Hons) Nursing (Adult) [pre registration nursing]
**University of East Anglia**	BSc (Hons) nursing - Adult
**University Of Huddersfield**	BSc (Hons) Nursing - Adult
**University Of The West Of England, Bristol**	BSc (Hons) Nursing - Adult
**University of East Anglia**	BSc (Hons) Nursing - Adult Degree Apprenticeship
**Queen Margaret University**	BSc (Hons) Nursing
**University Of Plymouth**	BSc (Hons) Nursing (Adult Health)
**University Of Plymouth**	BSc (Hons) Nursing (Adult Health) - Nurse Degree Apprenticeship
**University Of York**	BSc (hons) Nursing (Adult)
**University of Salford**	BSc (Hons) Nursing (adult)
**University of Bolton**	BSc (Hons) Nursing (adult)
**University of Nottingham**	BSc (Hons) Nursing (adult)
**Buckinghamshire New University**	BSc (Hons) Nursing (Adult)
**Anglia Ruskin University**	BSc (Hons) Nursing (Adult)
**Swansea University**	BSc (Hons) Nursing (Adult)
**Edge Hill University**	BSc (Hons) Nursing (Adult)
**Edge Hill University**	BSc (Hons) Nursing (Adult)
**The Open University**	BSc (Hons) Nursing (Adult)
**The University of Hull**	BSc (Hons) Nursing (Adult)
**The University of Hull**	BSc (Hons) Nursing (Adult)
**University of Ulster**	BSc (Hons) Nursing (Adult)

For peer review only

Approved education institution	Course
**University Of East London**	BSc (Hons) Nursing (Adult)
**University Of Leeds**	BSc (Hons) Nursing (Adult)
**University College Birmingham**	BSc (Hons) Nursing (Adult)
**University Of Southampton**	BSc (Hons) Nursing (Adult)
**Canterbury Christ Church University**	BSc (Hons) Nursing (Adult)
**Birmingham City University**	BSc (Hons) Nursing (Adult)
**Anglia Ruskin University**	BSc (Hons) Nursing (Adult)
**Swansea University**	BSc (Hons) Nursing (Adult)
**The Robert Gordon University**	BSc (Hons) Nursing (Adult)
**University of Bradford**	BSc (Hons) Nursing (Adult)
**The Open University**	BSc (Hons) Nursing (Adult)
**The Open University**	BSc (Hons) Nursing (Adult)
**University of Gloucestershire**	BSc (Hons) Nursing (Adult)
**University Of Hertfordshire**	BSc (Hons) Nursing (adult) - Nursing Degree Apprenticeship
**The Open University**	BSc (Hons) Nursing (Adult) - Nursing Degree Apprenticeship
**Birmingham City University**	BSc (Hons) Nursing (Adult) - Nursing Degree Apprenticeship
**University of Gloucestershire**	BSc (Hons) Nursing (Adult) (Apprenticeship)
**University of Gloucestershire**	BSc (Hons) Nursing (Adult) (Blended Learning)
**Edge Hill University**	BSc (Hons) Nursing (Adult) (fall back award)
**University Of Leeds**	BSc (Hons) Nursing (Adult) (International)
**Canterbury Christ Church University**	BSc (Hons) Nursing (Adult) (NDA)
**University of Bolton**	BSc (Hons) Nursing (Adult) (Part-time)

For peer review only

Approved education institution	Course
**University of Bolton**	BSc (Hons) Nursing (Adult) (Registered Nursing Higher Apprenticeship) (Part-time)
**University of Bradford**	BSc (Hons) Nursing (Adult) [fall back award]
**Buckinghamshire New University**	BSc (Hons) Nursing (Adult) Apprenticeship
**University of Salford**	BSc (Hons) Nursing (adult) Nurse Degree Apprentice
**University of Bolton**	BSc (Hons) Nursing (adult) nurse degree apprenticeship (NDA) route
**Middlesex University**	BSc (Hons) Nursing (adult) seconded pathway
**De Montfort University**	BSc (Hons) Nursing (Adult) with NMC Registration (Apprenticeship route)
**University Of East London**	BSc (Hons) Nursing (Adult)-Registered Nurse (Degree) Apprenticeship Route
**Queen Margaret University**	BSc (Hons) Nursing (fall back award)
**University of Lincoln**	BSc (Hons) Nursing (Registered Nurse - Adult)
**University of South Wales**	BSc (Hons) Nursing Adult
**Nottingham Trent University**	BSc (Hons) Nursing Adult
**University Of Brighton**	BSc (Hons) Nursing Adult
**University of Worcester**	BSc (Hons) Nursing Adult
**Sheffield Hallam University**	BSc (Hons) Nursing Adult (Part Time)
**University Of The West Of England, Bristol**	BSc (Hons) Nursing apprenticeship - Adult
**University Of Huddersfield**	BSc (Hons) Nursing Child (Blended Learning)
**University of Sunderland**	BSc (Hons) nursing degree apprenticeship (adult)
**Nottingham Trent University**	BSc (Hons) Nursing Degree Apprenticeship Adult
**Swansea University**	BSc (Hons) Nursing part-time (Adult)

For peer review only - http://bmjopen.bmj.com/site/about/guidelines.xhtml

Approved education institution	Course
**University Of Northumbria At Newcastle**	BSc (Hons) nursing science (adult)
**University Of Northumbria At Newcastle**	BSc (Hons) nursing science apprenticeship (adult)
**Teesside University**	BSc (Hons) Nursing Studies (Adult) (Pre-registration) Apprenticeship route
**Glasgow Caledonian University**	BSc (Hons) Nursing Studies: Adult Nursing
**Liverpool John Moores University**	BSc (Hons) Nursing with Registered Nurse Status (Adult)
**Liverpool John Moores University**	BSc (Hons) Nursing with Registered Nurse Status (Adult)
**University of Cumbria**	BSc (Hons) Nursing/RN:Adult Nursing
**University of Cumbria**	BSc (Hons) Nursing/RN:Adult Nursing (Apprenticeship)
**Keele University**	BSc (Hons) Nursing: Adult
**Keele University**	BSc (Hons) Nursing: Adult (NDA)
**Kingston University & St George's University of London**	BSc Adult Nursing
**University Of Dundee**	BSc Adult Nursing
**University of West of Scotland**	BSc Adult Nursing
**University Of Greenwich**	BSc Adult Nursing (Degree Apprenticeship) (fall back award)
**University Of Greenwich**	BSc Adult Nursing (fall back award)
**Middlesex University**	BSc Hons Nursing Adult
**University of Highlands and Islands**	BSc Nursing
**City, University of London**	BSc Nursing (Adult)
**Oxford Brookes University**	BSc Nursing (Adult)
**The Robert Gordon University**	BSc Nursing (Adult)

Approved education institution	Course
**The Robert Gordon University**	BSc Nursing (Adult)
**University Of Stirling**	BSc Nursing (Adult)
**The Robert Gordon University**	BSc Nursing (Adult) (fall back award)
**Glasgow Caledonian University**	BSc Nursing Studies: Adult Nursing
**King's College London**	BSc Nursing with Registration as an Adult Nurse
**University of Ulster**	BSc(Hons) Nursing (Adult)
**University of Lincoln**	Degree Science Nursing (Registered Nurse - Adult: Apprenticeship Route)
**University of Salford**	MA Nursing (adult)
**University Of Wolverhampton**	Master of Adult Nursing
**University Of Wolverhampton**	Master of Nursing (Adult Nursing)
**University Of Birmingham**	Master of Nursing (Adult)
**University Of Wolverhampton**	Master of Nursing (MNurs) Adult Nursing
**University Of Birmingham**	Master of Science in Nursing (Adult)
**University of Derby**	Master of Science in Nursing (Adult)
**University of Derby**	Master of Science in Nursing (adult) (apprenticeship)
**University Of Manchester**	Masters in Nursing (Adult)
**University Of Edinburgh**	Masters in Nursing with pre-registration (adult)
**University Of Sheffield**	MMedSci Nursing (Adult)
**Queen Margaret University**	MN Nursing
**Edge Hill University**	MNSW Adult Nursing and Social Work
**University of Sunderland**	MNurse (Adult)
**University Of York**	MNursing (Adult)
**University Of Hertfordshire**	MSc Hons Nursing (Adult)

For peer review only - http://bmjopen.bmj.com/site/about/guidelines.xhtml

Approved education institution	Course
**University Of Northumbria At Newcastle**	MSc (Hons) nursing science (adult)
**University Of Dundee**	MSc Adult Nursing
**University Of Greenwich**	MSc Adult Nursing
**University of Chester**	MSc Adult Nursing
**University of Chester**	MSc Adult Nursing
**University of Chester**	MSc Adult Nursing
**University of Bedfordshire**	MSc Adult Nursing
**London South Bank University**	MSc adult nursing
**University of West of Scotland**	MSc Adult Nursing
**University of Chester**	MSc Adult Nursing
**Bournemouth University**	MSc Adult Nursing
**Kingston University & St George's University of London**	MSc Adult Nursing
**Coventry University**	MSc Adult Nursing
**University of Bedfordshire**	MSc Adult Nursing (apprenticeship)
**Birmingham City University**	MSc Adult Nursing (pre-registration)
**Manchester Metropolitan University**	MSc Adult Nursing (Pre-registration)
**University of Chester**	MSc Adult Nursing Apprenticeship
**Coventry University**	MSc Adult Nursing Blended Learning (pre-registration)
**Coventry University**	MSc Adult Nursing Blended Learning (pre-registration) Nursing Degree Apprenticeship
**University Of Southampton**	MSc Nursing (Adult)
**Canterbury Christ Church University**	MSc Nursing (Adult)
**City, University of London**	MSc Nursing (Adult)

Approved education institution	Course
**University Of Hertfordshire**	MSc Nursing (adult) - Nursing Degree Apprenticeship (part time route)
**University of Nottingham**	MSc nursing (graduate entry) (adult)
**University of Lincoln**	MSc Nursing (Pre-Registration – Adult)
**Edge Hill University**	MSc Nursing (Pre-registration Adult)
**University Of Huddersfield**	MSc Nursing (pre-registration) - Adult
**Swansea University**	MSc Nursing Pre-registration (Adult)
**Teesside University**	MSc Nursing Studies (Adult Apprenticeship)
**Glasgow Caledonian University**	MSc Nursing Studies: Adult Nursing
**University of Central Lancashire**	MSc nursing with registered nurse (adult)
**Liverpool John Moores University**	MSc Nursing with Registered Nurse Status (Adult)
**Liverpool John Moores University**	MSc Nursing with Registered Nurse Status (Adult)
**King's College London**	MSc Nursing with Registration as an Adult Nurse
**University Of Plymouth**	MSc Pre-registration Nursing (Adult Health)
**University Of Huddersfield**	MSc Pre-registration Nursing apprenticeship-Adult
**University Of Huddersfield**	MSc Pre-registration Nursing Apprenticeship Child (Blended Learning)
**University Of Huddersfield**	MSc Pre-registration Nursing Child (Blended Learning
**University of Cumbria**	MSc Pre-Registration Nursing/Registered Nurse: Adult Nursing
**Edge Hill University**	MSci Nurse Paramedic (Adult)
**University Of Exeter**	MSci nursing (adult)
**University of Leicester**	MSci Nursing with Leadership Adult Nursing

For peer review only - http://bmjopen.bmj.com/site/about/guidelines.xhtml

Approved education institution	Course
**Middlesex University**	Nurse Degree Apprenticeship Adult (4 year direct entry route)
**Middlesex University**	Nurse Degree Apprenticeship Adult (4 year part-time employed route)
**University of West London**	Nurse Degree Apprenticeship BNursing (Hons) Adult
**University of Central Lancashire**	Nursing Degree Apprenticeship route – Adult
**Teesside University**	Nursing Studies (Adult) (Pre-registration) BSc (Hons)
**Teesside University**	Nursing Studies (Adult) (Pre-Registration) MSc
**University of West of Scotland**	PG Dip Adult Nursing
**London South Bank University**	PG Dip adult nursing
**University Of Greenwich**	PG Dip Adult Nursing (fall back award)
**University of South Wales**	PG Dip Nursing Adult
**University of West London**	PG Diploma Nursing Adult
**Edge Hill University**	PgDip Nursing (second registration Adult)
**University Of Southampton**	Postgraduate Diploma Nursing (Adult)
**The Queen's University Of Belfast**	Pre-registration nursing - Adult
**Sheffield Hallam University**	Pre-registration nursing - Adult
**The Queen's University Of Belfast**	Pre-registration nursing - Adult (Graduate Entry)
**Staffordshire University**	Registered Nurse (Adult)
**University Of Brighton**	Registered Nurse Degree Apprenticeship (Adult)

UK M.S.C Approved Medicine and Surgery Programmes

Institution Name
University of Aberdeen School of Medicine and Dentistry
Anglia Ruskin University School of Medicine
Aston University Medical School
Barts and The London School of Medicine and Dentistry
University of Birmingham College of Medical and Dental Sciences
Brighton and Sussex Medical School
University of Bristol Medical School
University of Buckingham Medical School
University of Cambridge School of Clinical Medicine
Cardiff University School of Medicine
University of Dundee School of Medicine
Edge Hill University Medical School
The University of Edinburgh Medical School
University of Exeter Medical School
University of Glasgow School of Medicine
Hull York Medical School
Imperial College London Faculty of Medicine
Keele University School of Medicine
Kent and Medway Medical School
King's College London GKT School of Medical Education
Lancaster University Medical School
University of Leeds School of Medicine
University of Leicester Medical School
University of Liverpool School of Medicine
University of Manchester Medical School
Newcastle University School of Medical Education
Norwich Medical School
University of Nottingham School of Medicine

For peer review only

University of Nottingham - Lincoln Medical School
University of Oxford Medical Sciences Division
Plymouth University Peninsula Schools of Medicine and Dentistry
Queen's University Belfast School of Medicine
University of Sheffield Medical School
University of Southampton School of Medicine
University of St Andrews School of Medicine
St George's, University of London
University of Sunderland School of Medicine
Swansea University Medical School
University of Central Lancashire School of Medicine
University College London Medical School
University of Warwick Medical School

For peer review only

Patient Information Section

General information

The aim of this study is to determine the impact of the COVID-19 pandemic on the mental health of UK medical and nursing students, one year down the line from the initial outbreak. We are inviting all medical and nursing students enrolled at university in the UK to participate in this study. Please read the following section carefully before taking part. If you have any questions regarding the study please contact the research team via the following email: kate.saunders@psych.ox.ac.uk

What is the purpose of the study?

The purpose of SPICE-20 is to evaluate the continued impact of the COVID-19 pandemic on UK medical students, one year on from the SPICE-19 study. In addition, we aim to determine the effect on UK nursing students. In doing so we hope to provide feedback to UK medical schools and Nursing institutions about the current collective mental health of student healthcare professionals. We believe this information is vital to ensure adequate mental wellbeing service provision is in place at higher education institutions.

Why am I being invited to participate in this study?

You have been invited to take part in this study because we are recruiting medical and nursing students to participate and feedback on their continued mental wellbeing during the UK pandemic. You are eligible to participate if you are a current UK medical or nursing student in the UK and have access to the internet.

What happens if I choose to participate?

Participation in this study is entirely voluntary. To be involved you will be required to complete a short survey online. The survey will ask for general background details about you including: your current educational status, age, gender, ethnicity. Additionally, questions will ask about your current mood and mental wellbeing. Your decision to participate in this study will not have any academic impact and will be entirely anonymous. The survey is expected to take approximately 10-15 minutes to complete however it may be done as quick or slow as you like. There is no prior knowledge required to participate. We will ask for your consent for collection and storage of data in accordance with General

For peer review only

Data Protection Regulation (GDPR) before the survey begins. More information regarding the rules and regulations of GDPR can be found at www.gdpr-info.eu .

Do I have to participate?

As stated above, participation in the SPICE-20 study is entirely voluntary and anonymous. Consent for involvement may be withdrawn anytime prior to completion of the survey. To do so, simply close the tab or browser containing the survey. Following this, no personal data provided up to that point in the survey will be stored or used for research or any other purpose. Completion of all questions is entirely optional. This means that participation is still possible even if there are certain questions you would prefer not to answer. If there is a question you would like to omit from your responses, simply select the option "Prefer not to answer". Any data collected will not be provided to your higher education institution without being anonymised, meaning your feedback will not make your identifiable.

Are there any risks of being involved?

Some of the questions included in the questionnaire may potentially cause some distress by brining up painful memories. If at any point during completion of the survey you are feeling distressed you may stop or choose not to answer troubling questions. Included with the study information is mental wellbeing support services that are available to access.

Are there any benefits to being involved?

The completion of this survey will not provide any academic benefit at your respective institutions. However, the results of SPICE-20 will be used to inform university wellbeing service provision. This means that by participating in the study you have the opportunity to provide anonymous feedback to your institution about what you feel is being done well, or could be done better to support students. Additionally, this survey may provide be useful as a personal opportunity for reflection.

How will my data be used?

Your answers will be completely anonymous and no identifiable data will be included. Data will be stored in a password protected file and will only be accessible by individuals on the steering/writing committee that have a reason to access it. IP addresses will not be stored. If you provide your email address, it will be deleted at the end of the study and not linked to any information you provide.

For peer review only

Anonymised responses to the survey will be stored for a minimum of ten years after publication or public release.

Who will have access to your data?

Qualtrics is the data platform used for SPICE-20. They are the data controller in respect to the personal data they hold and as such they determine how personal data is used. Their privacy notice can be found at: www.qualtrics.com/privacy-statement. Qualtrics will share any email address you provide with the University of Oxford for the purposes of research only. Responsible members of the University of Oxford may be given access to your data for monitoring or audit of the study and ensuring that guidelines are complied with.

Where will the results be published?

The results of SPICE-20 may be published in a peer reviewed academic journal. In addition, the results of the study may be presented at conferences, either in oral or poster format. The study is targeting a completion date of September 2021. Following this provisional date a summary of the results and outcomes will be available on request to the study team.

Who do I contact if I have any queries or wish to make a complaint about the study?

Our study team aims to operate in a transparent manner. If you have any queries or complaints regarding the study aims, distribution, or any other aspect please contact the study team at kate.saunders@psych.ox.ac.uk and we will do our best to provide an adequate response. Our research team aims to reply within 10 working days. If you have any further issues or wish to escalate your inquiry please contact the Chair of the Medical Sciences Inter-Divisional Research Ethics Committee. Email: ethics@medsci.ox.ac.uk. Address: Research Services, University of Oxford, Wellington Square, Oxford, OX1 2JD.

Who do I contact for further details?

For any queries or other details, contact the principle investigator Professor Kate Saunders at kate.saunders@psych.ox.ac.uk.

For peer review only

Please check the box to confirm you are the age of 18 years old or older

☐ I certify that I am 18 years or older.

If you have read the information above and agree to participate with the understanding that the data (including personal data) you submit will be processed accordingly, please check the relevant box to get started

☐ Yes, I agree to take part.

For peer review only

STROBE Statement—checklist of items that should be included in reports of observational studies

	Item No.	Recommendation	Page No.	Relevant text from manuscript
**Title and abstract**	1	(*a*) Indicate the study's design with a commonly used term in the title or the abstract	P1, Title	
		(*b*) Provide in the abstract an informative and balanced summary of what was done and what was found	P2, Abstract	
**Introduction**				
Background/rationale	2	Explain the scientific background and rationale for the investigation being reported	P3	
Objectives	3	State specific objectives, including any prespecified hypotheses	P4, Last Paragraph	
**Methods**				
Study design	4	Present key elements of study design early in the paper	From P4	
Setting	5	Describe the setting, locations, and relevant dates, including periods of recruitment, exposure, follow-up, and data collection	From P4 To P8	
Participants	6	(*a*) *Cohort study*—Give the eligibility criteria, and the sources and methods of selection of participants. Describe methods of follow-up	N/A	
		Case-control study—Give the eligibility criteria, and the sources and methods of case ascertainment and control selection. Give the rationale for the choice of cases and controls	N/A	
		Cross-sectional study—Give the eligibility criteria, and the sources and methods of selection of participants	P6	
		(*b*) *Cohort study*—For matched studies, give matching criteria and number of exposed and unexposed	N/A	
		Case-control study—For matched studies, give matching criteria and the number of controls per case	N/A	
Variables	7	Clearly define all outcomes, exposures, predictors, potential confounders, and effect modifiers. Give diagnostic criteria, if applicable	P5	
Data sources/ measurement	8*	For each variable of interest, give sources of data and details of methods of assessment (measurement). Describe comparability of assessment methods if there is more than one group	P5	
Bias	9	Describe any efforts to address potential sources of bias	N/A	
Study size	10	Explain how the study size was arrived at	P7	

Continued on next page

For peer review only - http://bmjopen.bmj.com/site/about/guidelines.xhtml
For peer review only

Quantitative variables	11	Explain how quantitative variables were handled in the analyses. If applicable, describe which groupings were chosen and why	P7
Statistical methods	12	(*a*) Describe all statistical methods, including those used to control for confounding	P7
		(*b*) Describe any methods used to examine subgroups and interactions	N/A
		(*c*) Explain how missing data were addressed	N/A
		(*d*) *Cohort study*—If applicable, explain how loss to follow-up was addressed	N/A
		Case-control study—If applicable, explain how matching of cases and controls was addressed	N/A
		Cross-sectional study—If applicable, describe analytical methods taking account of sampling strategy	P5
		(*e*) Describe any sensitivity analyses	N/A
**Results**			
Participants	13*	(a) Report numbers of individuals at each stage of study—eg numbers potentially eligible, examined for eligibility, confirmed eligible, included in the study, completing follow-up, and analysed	N/A
		(b) Give reasons for non-participation at each stage	N/A
		(c) Consider use of a flow diagram	N/A
Descriptive data	14*	(a) Give characteristics of study participants (eg demographic, clinical, social) and information on exposures and potential confounders	N/A
		(b) Indicate number of participants with missing data for each variable of interest	N/A
		(c) *Cohort study*—Summarise follow-up time (eg, average and total amount)	N/A
Outcome data	15*	*Cohort study*—Report numbers of outcome events or summary measures over time	N/A
		Case-control study—Report numbers in each exposure category, or summary measures of exposure	N/A
		Cross-sectional study—Report numbers of outcome events or summary measures	N/A
Main results	16	(*a*) Give unadjusted estimates and, if applicable, confounder-adjusted estimates and their precision (eg, 95% confidence interval). Make clear which confounders were adjusted for and why they were included	N/A
		(*b*) Report category boundaries when continuous variables were categorized	N/A
		(*c*) If relevant, consider translating estimates of relative risk into absolute risk for a meaningful time period	N/A

Continued on next page

For peer review only - http://bmjopen.bmj.com/site/about/guidelines.xhtml

For peer review only

Other analyses	17	Report other analyses done—eg analyses of subgroups and interactions, and sensitivity analyses	N/A
**Discussion**			
Key results	18	Summarise key results with reference to study objectives	N/A
Limitations	19	Discuss limitations of the study, taking into account sources of potential bias or imprecision. Discuss both direction and magnitude of any potential bias	From P9 To P10
Interpretation	20	Give a cautious overall interpretation of results considering objectives, limitations, multiplicity of analyses, results from similar studies, and other relevant evidence	N/A
Generalisability	21	Discuss the generalisability (external validity) of the study results	N/A
**Other information**			
Funding	22	Give the source of funding and the role of the funders for the present study and, if applicable, for the original study on which the present article is based	P11

*Give information separately for cases and controls in case-control studies and, if applicable, for exposed and unexposed groups in cohort and cross-sectional studies.

Note: An Explanation and Elaboration article discusses each checklist item and gives methodological background and published examples of transparent reporting. The STROBE checklist is best used in conjunction with this article (freely available on the Web sites of PLoS Medicine at http://www.plosmedicine.org/, Annals of Internal Medicine at http://www.annals.org/, and Epidemiology at http://www.epidem.com/). Information on the STROBE Initiative is available at www.strobe-statement.org.

For peer review only - http://bmjopen.bmj.com/site/about/guidelines.xhtml

BMJ Open

Social and Psychological Impact of the Covid-19 pandemic on UK medical and nursing students: Protocol for a national medical and nursing student survey

Journal:	*BMJ Open*
Manuscript ID	bmjopen-2021-057467.R2
Article Type:	Protocol
Date Submitted by the Author:	09-Apr-2022
Complete List of Authors:	Richardson, George; University of Liverpool School of Medicine, Institute of Translational Medicine<br>Gillespie, Conor; University of Liverpool, Institute of Systems Molecular and Integrative Biology<br>Mantle, Orla; King's College London Faculty of Life Sciences and Medicine<br>Clynch, Abigail; University of Liverpool, Institute of Systems Molecular and Integrative Biology<br>Ooi, Setthasorn Zhi Yang; Cardiff University School of Medicine<br>Park, Jay; The University of Edinburgh Edinburgh Medical School<br>Bligh, Emily R.; The University of Sheffield, School of Medicine<br>Kundu, Shantanu; University of Liverpool, School of Medicine<br>Georgiou, Ioannis; University of Aberdeen, School of Medicine<br>Bandyopadhyay, Soham; University of Oxford, Oxford University Medical School<br>Saunders, Kate E ; University of Oxford, Department of Psychiatry; Oxford Health NHS Foundation Trust<br>Interest Group, Neurology and Neurosurgery; Neurology and Neurosurgery Interest Group<br>SPICE-20, Collaborative; SPICE-20 Collaborative
**Primary Subject Heading**:	Medical education and training
Secondary Subject Heading:	Medical education and training, Mental health
Keywords:	MENTAL HEALTH, MEDICAL EDUCATION & TRAINING, Child & adolescent psychiatry < PSYCHIATRY

SCHOLARONE™
Manuscripts

1

Social and Psychological Impact of the Covid-19 pandemic on UK medical and nursing students: Protocol for a national medical and nursing student survey

Authors and affiliations:
George E. Richardson*[1], Conor S. Gillespie*^[2], Orla Mantle[3], Abigail Clynch[2], Setthasorn Z. Y. Ooi[4], Jay Park[5], Emily R. Bligh[6], Shantanu Kundu[7], Ioannis Georgiou[8], Soham Bandyopadhyay[9], Kate E.A. Saunders[10,11], Neurology and Neurosurgery Interest Group (NANSIG), SPICE-20 Collaborative.

[1]Institute of Translational Medicine, University of Liverpool, Liverpool, UK
[2]Institute of Systems, Molecular and Integrative Biology, University of Liverpool, Liverpool, UK
[3]Faculty of Life Sciences and Medicine, King's College London, London, United Kingdom
[4]Cardiff University School of Medicine, University Hospital of Wales Main Building, Heath Park, Cardiff, UK
[5]Edinburgh Medical School, University of Edinburgh, Edinburgh, UK
[6]School of Medicine, University of Sheffield, Sheffield, UK
[7]School of Medicine, University of Liverpool, Liverpool, UK
[8]School of Medicine, University of Aberdeen, Aberdeen, UK
[9]Oxford University Global Surgery Group, Nuffield Department of Surgical Sciences, Medical Sciences Division, University of Oxford, Oxford, UK.
[10]Department of Psychiatry, Warneford Hospital, University of Oxford, Warneford Lane, Oxford, UK
[11]Oxford Health NHS Foundation Trust, Warneford Hospital, University of Oxford, Warneford Lane, Oxford, UK

*Joint first author, each author contributed equally to the work.

^Corresponding author

Abstract word count: 296

Full text word count: 2804

Corresponding author contact details:
Conor S. Gillespie
Institute of Systems, Molecular and Integrative Biology (ISMIB)
University of Liverpool
Biosciences Building
Crown Street
Liverpool
L69 7BE
Email address: hlcgill2@liv.ac.uk

For peer review only

Keywords: Wellbeing, Mental Health, Survey, Medical Students

ABSTRACT

Introduction

Healthcare students have played a significant role in the NHS during the COVID-19 pandemic. We captured data on the wellbeing of medical students during the acute phase of the pandemic with the Social and Psychological Impact of COVID-19 on medical students: a national survey Evaluation (SPICE-19) study. We will evaluate changes in mental health and wellbeing of medical and nursing students one year after SPICE-19, in a cross-sectional study, to understand the impact of the pandemic, and inform wellbeing policies.

Methods and Analysis

This study will be a national, multi-institution, cross-discipline study. An online, 53-item survey of demographics, mental health and wellbeing will be used to record responses. Students studying for a medical or nursing degree at any UK universities will be eligible to participate. The survey will be advertised through the Neurology and Neurosurgery Interest Group (NANSIG) national network. Participation is anonymous and voluntary, with relevant mental health resources made available to participants.

Ethics and Dissemination

Ethical approval was granted by the University of Oxford Central University Research Ethics Committee (R75719/RE001) on 21st May 2021. Study findings will be presented at national and international meetings, and submitted for publication in a peer-reviewed journal.

Strengths and limitations

- This is a multi-centre, national questionnaire-based survey of medical and nursing student mental health and wellbeing in the UK.
- This is the first study to assess mental health and wellbeing of the student multi-disciplinary team during the new normal after the Covid-19 pandemic.

For peer review only

3

- The study results will provide valuable insight into healthcare workers, which can be used to identify and target further areas of wellbeing support and inform policy.

- Response bias may overestimate participation of students based on age, sex, and medical/nursing school, with those interested in neuroscience and mental health more likely to participate.

INTRODUCTION

Medical and nursing students in the UK have been significantly affected by the COVID-19 pandemic. During the first wave, many were active components of the clinical team, either by undertaking extending clinical placements, paid work in the NHS, or through voluntary roles, while others saw placements reduced, suspended, or cancelled completely.(1, 2) We evaluated medical student wellbeing and mental health during the acute phase of the pandemic with the Social and Psychological Impact of COVID-19 on medical students: a national service evaluation (SPICE-19) study,(3) a cross sectional study with a prospective component, which identified several points for service provision improvement based on 2275 responses across 34 medical schools in the UK.(4-6)

It is pertinent to re-evaluate the mental health of medical students, one year removed from SPICE-19, to explore how they are navigating their training, education, and the impact of adapting to the 'new normal' of medical education provision.(7, 8) It is also important to elucidate the effect of COVID-19 on the wellbeing and mental health of UK nursing students, many of whom were asked to volunteer on the front line in COVID wards,(2) and a large-scale study examining this has not been completed. A single centre survey in the US noted that nursing students reported increased levels of anxiety, reduction in concentration, and difficulties with academic workload throughout the pandemic,(9) and a large multi-institutional study is required to elucidate this further. This is in accordance with previously published work by the SPICE-19 research team. (10, 11)

Medical and nursing schools have each responded differently to the pandemic, with the accessibility of wellbeing and mental health services variable between universities.(12) A national survey assessing mental health and wellbeing in medical and nursing students, as well as support systems available for those facing mental health difficulty is required. Evaluating the mental health and wellbeing of the student multi-disciplinary team is vital for

For peer review only

ensuring adequate service provision is provided - it is paramount to safeguard the wellbeing of the next generation of healthcare professionals.(13, 14)

The primary aim of this study is to comprehensively evaluate mental health and wellbeing of medical and nursing students during the evolving COVID-19 pandemic, one year after the initial outbreak. The secondary aims are to elucidate students' perceptions of institutional efforts to provide support, and accessibility and appropriateness of such support. The study logos are shown in Figure 1.

Figure 1. SPICE-20 study logos.

METHODS AND ANALYSIS

Study partners

NANSIG

The Neurology and Neurosurgery Interest Group (NANSIG) was formed in 2010. It is a student and junior doctor led interest group designed to increase participation, diversity and engagement in Neurosciences, and has over 1000 members internationally.(15) The organisation has an affiliation with the Society of British Neurosurgeons (SBNS) and the Association of British Neurologists (ABN) (www.nansig.org). The organisation led the SPICE-19 national cohort study last year, assessing wellbeing in 2275 medical students in response to the pandemic, and is familiar with survey design and distribution. The collective organisation runs established national and international events, and has published over 20 peer-reviewed publications.

Study design

SPICE-20 is a national, multi-institution, cross-sectional study. An online survey will be used to record participant responses. The survey contains 53 items - section 1 of the survey includes background and demographic information. Section 2 assesses wellbeing by asking participants to complete the Warwick-Edinburgh Mental Well Being Scale (WEMWBS); a 14-point, validated evaluation of wellbeing.(16) Section 3 encompasses the Patient Health Questionnaire-9 (PHQ-9) and Generalised Anxiety Disorder-7 (GAD-7). The PHQ-9 is a valid diagnostic and severity measure for depressive disorder,(17) and GAD-7 is a seven-item,

For peer review only

validated assessment of generalised anxiety.(18, 19) Section 4 includes questions about university support offered by each institution, if participants had accessed this support, and any significant changes to teaching encountered during the pandemic. Section 4 contains study specific unvalidated questions. These questions were defined for the current study to meet the specific secondary aims and were not adapted from the SPICE-19 survey, due to a difference in study objectives.

The survey was iteratively defined by the student-led study management team until a consensus was reached. Based on our experience with SPICE-19 survey, we have reduced the number of questions and limited the survey to validated measures of wellbeing and mental health. The final, 53-item questionnaire is shown in Figure 2.

Figure 2: Final SPICE-20 survey design.

Survey piloting

The survey was piloted by a group of 15 medical students from the NANSIG core committee. These students were not involved in the design of the study and were consulted in order to provide feedback, improve clarity and ensure objectivity. Data was analysed to identify any points of concern, estimated completion time, and difficult survey items/questions. Students were contacted to identify any suggestions for improvement, with none identified. Therefore, no further alterations were made.

Survey administration

The survey was hosted on the Qualtrics™ survey platform (Provo, Utah, USA), a GDPR-compliant online survey platform, that facilitates both mobile and desktop devices.

Study dissemination

To maximise distribution across the UK, a national network of SPICE-20 Collaborative members were recruited, representing all 33 medical schools in the UK, using a purposive sampling method. Most held concurrent committee or regional lead roles in NANSIG at time of recruitment. Each member was asked to acquire the most up to date (as of April 2021)

For peer review only

available resources, guidance and support policies in place for their current medical school, plus an additional nursing school. A standard advertising method was developed by the steering committee and based on the successful recruitment strategy employed in the SPICE-19 study, which involved members contacting their respective medical school deans to request distribution of the survey at inception, as well as regular advertising over the 8-week study collection period, from 7st June 2021 to the 7rd August 2021. The distribution links for the survey includes university mailing lists, student society pages, and social media platforms. Figure 3 provides an example of the email template used to disseminate the survey through student mailing lists. Adverts will also be placed on NANSIG social media platforms, as well as the monthly NANSIG newsletter.

In the invitation to complete the study, based on their medical/nursing school, participants will also receive wellbeing, mental health, and guidance services specific to their institution, should they need to access them at any point. Participants will also be made aware of our patient and public involvement organisation, and their contact details. No incentives for participation will be offered.

Figure 3. Standard email template used for all survey dissemination through student email lists, developed by the study team and approved by the research ethics committee.

Eligibility and representation

All current students enrolled at UK medical schools recognised by the General Medical Council (GMC) and the Medical Schools Council (MSC) will be eligible to participate. Additionally, all current home and international students enrolled on an adult, child and mental health nursing degree at a UK university are eligible for inclusion. A list of eligible medical schools, and approved programmes by the Nursing and Midwifery Council (NMC) is included in the supplementary materials (document "Supplementary Material – MSC and NMC").

Consent and confidentiality

A patient information sheet will be shown on the first page of the study (supplementary material - patient information). This encompasses the rationale, purpose, and voluntary nature of participation. Participants must verify that they are over 18 years old and provide

For peer review only

informed consent via tick box. It will be emphasised that participation is anonymous, confidential, voluntary, and that participants can withdraw consent at any time.

Data security

All data will be collected and stored on the secure online server, Qualtrics™. Survey data will be extracted from the software to a password-protected, Microsoft Excel spreadsheet (Microsoft, California, USA) only available to the study team. Data handling and record keeping will adhere to the University of Oxford standards for data security.

Statistical analysis

Data will be analysed using R version 4.0.3. The overall wellbeing of medical and nursing students, as assessed using the WEMWBS, and mental health, using the PHQ-9 and GAD-7, will be presented using descriptive statistics. The scores of WEMWBS, PHQ-9 and GAD-7 will be categorised using established cut off points into dichotomous groups, corresponding to individuals expressing symptoms and those which do not. (20-22) No information that identifies or is specific to institutions will be presented. Appropriate statistical methods will be selected based on the distribution and type of data; it is anticipated data will be non-parametric, and, will therefore be compared using tests such as $\chi 2$ or Kruskal-Wallis tests. Differences between medical and nursing students will be compared using these statistical tests. To identify factors associated with an increased scores on WEMWBS, PHQ-9, and GAD-7, multi-variate generalised linear regression will be used.

Sample size calculation

Approximate estimates of total UK medical and nursing students were calculated from yearly undergraduate intake figures. The total number of UK medical and adult nursing students was estimated to be 84,000. An online calculator was used to determine the sample size needed for the survey. The minimum sample size required from our target population for a margin error of 5% (around a 50% distribution) and a confidence interval level of 95% is 383, assuming a 50% response distribution for each question, the most conservative response distribution for study power.

Patient and public involvement

For peer review only

As the study population was medical and nursing student wellbeing and mental health, we worked collaboratively with the Be Free Campaign - a mental health and wellbeing awareness charity, from study inception. Be Free were involved in the conception, design and development of the study. The charity works with young people and students, emphasising mental health and wellbeing, to tackle the stigma behind mental health, and promote expression of individual values (UK registered charity number 1189704). The charity director and clinical study team for the charity comprehensively reviewed the protocol for appropriateness of content, question format, and ensured the questions asked were sensitive and pertinent to medical and nursing students.

Be Free have extensive knowledge of indicators of mental health, and the questions selected (WEMWBS, PHQ-9 and GAD-7) were done so in concordance with their advice. The number of questions, layout, and design were also prepared in accordance with their suggestions. The charity inspired the conception of this study and approved the final survey design. The charity founder and director (SK) is also a member of the steering committee for this study.

DISCUSSION

Study rationale

International multi-institutional studies have demonstrated that during the initial COVID-19 outbreak, medical students were less likely to experience deterioration in mental health and wellbeing when compared to non-medical students. (23, 24) This is the first national, multi-institutional study to evaluate the mental health of UK medical and nursing students during the COVID-19 pandemic.(25) It will therefore be possible to elucidate the mental health and wellbeing of healthcare students across the UK. By comparing medical and nursing schools, it will be possible to evaluate the nature of successful support, as well as students' confidence and ability to access this support. The findings will inform service provision, and highlight areas where improvement is required. The survey will also highlight local and national wellbeing resources available to students.

Why assess medical and nursing student wellbeing and mental health?

For peer review only

Medical and nursing students form the healthcare multi-disciplinary team of the future. Mental health and wellbeing are positively correlated with job satisfaction, retention, and performance, and is an essential component of good health.(26) If the NHS is to retain, recruit and develop its workforce of the future, ascertaining their mental health at this point in the COVID-19 pandemic is essential. Young people have been disproportionately affected by the pandemic, and most medical and nursing students fall into this age category. As non-emergency care is delayed and rescheduled, students will have to deal with the long acting repercussions of the pandemic, including those directly affected by the virus itself, including long-COVID,(27) declining mental health in the population exacerbated by the pandemic,(28) and implications of delayed access to healthcare.(29, 30)

Many medical students may not be aware that such support exists, either at their respective institution, or at a regional/national level, and we would do these students a disservice by not highlighting existing mental health and wellbeing services.

Benefits of a national medical student wellbeing survey

Medical and nursing schools across the UK adopt heterogenous practices to achieve the outcomes set in the GMC and NMC core competencies and curricula.(31) A survey of both medical and nursing students on a national scale is the best way to evaluate mental health and wellbeing. Many institutions reacted differently to the pandemic, and a survey is best placed to simultaneously decipher wellbeing and mental health at each institution, while assessing the impact of the pandemic on a national scale, and highlighting heterogeneity between institutions. Furthermore, there is a need to appreciate that multi-institutional studies of large magnitude are required to answer important clinical questions and elucidate concerns not identified by smaller studies. Multi-centre studies are also less susceptible to identity issues because the data is aggregated between multiple medical schools, and can be presented as all centres.

Limitations

Online surveys have the advantage of ubiquitous presence, and increased accessibility to medical and nursing schools in the UK. However, several limitations can arise from such a study method.

For peer review only

10

Firstly, those with an existing interest in wellbeing and mental health may be more likely to engage with and complete the survey. This could lead to selection bias towards those adversely affected by the pandemic. The anticipated magnitude of this effect is unclear. In addition, as the survey is being distributed through NANSIG, there may be over-participation from students with an interest in Neurosciences and their surrounding connections.

In order to ameliorate this as best as possible, we have adopted a varied dissemination approach. This includes having representatives at each UK medical school, survey distribution to medical and nursing school bulletins, and newsletters. We hope this will improve visibility and participation. We decided not to incentivise participants with a potential prize for completing the survey, as we wanted responses to be motivated by a desire to complete the questions in as open a way as possible.

The study is also limited by its cross-sectional design, and will therefore only provide a snapshot of mental health and wellbeing at the time of survey completion. In addition, during capricious times such as COVID, fluctuations in mental health may occur in accordance with policy changes, as well as institutional policies towards placements and assessment, and we will assess date of study completion as a potential confounder as a variable in our analysis.

The self-reporting of symptoms will also be a significant limitation, in addition to the fact that the questions included are screening tools for mental health and wellbeing, and are not immediately diagnostic. Furthermore, a number of important variables including socio-economic status, co-morbid psychiatric conditions, and levels of isolation, have not been included within the survey.(32, 33) These factors are known to be associated with adverse mental health at baseline in young healthcare students,(34) and finances have been noted specifically to contribute to adverse medical and nursing student wellbeing.(32) The reason we omitted these from the survey, but included other variables such as ethnicity and religion, was for two reasons. First, it was felt that, during the conception of the study, that including religion and ethnicity as part of the participant demographics section was feasible, and would not affect participants' responses. Second, it was felt that requesting participants to divulge finances and report ongoing social isolation would be too intrusive, and is not a common component of participant demographics for most surveys. Omission of these and other variables may cause confounding of study results. The decision not to include these variables was made to ensure minimal barriers existed to completion of the survey, however we understand that the results must be interpreted within the context of this limitation.

For peer review only

Finally, there are no imbedded security or validation measures to confirm the medical or nursing school of each participant, and therefore we cannot guarantee that all participants were UK medical or nursing student

Ethics and dissemination

Ethical approval for this study was granted by the Oxford Research ethics committee [R75719/RE001]. The findings of the SPICE-20 study as described in this protocol, will be presented at scientific conferences, and submitted for publication in a peer-review journal once finalised.

Acknowledgments

The authors would like to thank the SPICE-19 collaborative, whose work inspired this study, and are grateful to the Be Free Campaign for their assistance with the conception, design and inspiration for components of the study. We also thank NANSIG and members of the SPICE-20 collaborative, for their kind assistance disseminating the survey.

Funding statement

No external funding has been sought in the conception, design or implementation of this study. CSG is a recipient of a grant from the Wolfson Foundation. GER is the recipient of funding from North West Cancer Research. KS is supported by the NIHR Oxford Health Biomedical Research Centre. The views expressed are those of the author(s) and not necessarily those of the NHS, the NIHR or the Department of Health.

Competing interests' statement

None to declare.

Presented at: N.A.

For peer review only

Author Contributions

GER, CSG, and OM were responsible for initial drafting of protocol manuscript, conceptualisation and designing the study. AC, SZYO, JP, ERB, IG, SB, and KS formed the wider study group and reviewed and approved drafts of the manuscript. KS provided supervision of the project. SK reviewed the manuscript and also provided PPI involvement and approval for the study. Neurology and Neurosurgery Interest Group (NANSIG) forms a collaborative that advised the conceptualisation, and initial design of the study. SPICE-20 Collaborative informed and approved the draft of the manuscript, and will ensure participant recruitment and completion of the final study.

Collaborators

Neurology and Neurosurgery Interest Group (NANSIG): Conor S Gillespie, Emily R Bligh, Soham Bandyopadhyay, Jay J Park, Setthasorn ZY Ooi, George E Richardson, Abigail Clynch, Oliver Burton, Avani Shanbhag, Moritz Steinruecke, William Bolton, Bharti Kewlani, Alvaro Yanez Touzet, Hannah Redpath, Seong Hoon Lee, Abdullah Egiz, Joshua Erhabor, Orla Mantle.

SPICE-20 Collaborative: Gideon Adegboyega, Soham Bandyopadhyay, Mohammed Talha Bashir, Prithvi Bahu, Emily Bligh, Oliver Burton, Yasmin Cantwell, Catinca Ciuculete, Abigail Clynch, George Davies, Anjali Deepak, Rosaline de Koning, Ritika Dilip, Tomas Ferreira, Ioannis Georgiou, Shankari Gnanakumar, Anita Golash, Conor S Gillespie, Hanya Ghazi, Rohan Gupta, La-Dantai Henriques, David Henshall, Tom Hess, Dana Hutton, Neslihan Isleyen, Fareha Khalil, Mehdi Khan, Shantanu Kundu, Electra Lerou, Cora Lowe, Orla Mantle, Setthasorn Zhi Yang Ooi, Mc Stephen Padilla, Jay J. Park, Jacob Porter, Hannah Redpath, George E Richardson, Sumaiya Rizaam, Kate E.A. Saunders, Jashan Selvakumar, Ishta Sharma, Amy Schranz, Anson Wong, Robyn Wilcha,

Data Sharing Statement

Data are available upon reasonable request. All data must be anonymised entirely prior to any sharing request completion.

REFERENCES

1. Miller DG, Pierson L, Doernberg S. The Role of Medical Students During the COVID-19 Pandemic. Annals of Internal Medicine. 2020;173(2):145-6.
2. Swift A, Banks L, Baleswaran A, Cooke N, Little C, McGrath L, et al. COVID-19 and student nurses: A view from England. Journal of Clinical Nursing. 2020;29(17-18):3111-4.
3. Soham B, Ioannis G, Bibire B, Conor SG, Marta de Andres C, Mohammad Talha B, et al. Research Square. 2021.
4. Coyle C, Ghazi H, Georgiou I. The mental health and well-being benefits of exercise during the COVID-19 pandemic: a cross-sectional study of medical students and newly qualified doctors in the UK. Irish Journal of Medical Science (1971 -). 2020.
5. Norton EJ, Georgiou I, Fung A, Nazari A, Bandyopadhyay S, Saunders KEA. Personal protective equipment and infection prevention and control: a national survey of UK medical students and interim foundation doctors during the COVID-19 pandemic. Journal of Public Health. 2020.
6. Georgiou I, Hounat A, Park JJ, Gillespie C, Bandyopadhyay S, Saunders KEA. The Factors That Influenced Medical Students' Decision to Work Within the NHS During the COVID-19 Pandemic-A National, Cross-sectional Study. J Occup Environ Med. 2021;63(4):296-301.
7. Lucey CR, Johnston SC. The Transformational Effects of COVID-19 on Medical Education. JAMA. 2020;324(11):1033-4.
8. Yuen J, Xie F. Medical education during the COVID-19 pandemic: perspectives from UK trainees. Postgraduate Medical Journal. 2020;96(1137):432.
9. Fitzgerald A, Konrad S. Transition in learning during COVID-19: Student nurse anxiety, stress, and resource support. Nurs Forum. 2021:10.1111/nuf.12547.
10. Coyle C, Ghazi H, Georgiou I. The mental health and well-being benefits of exercise during the COVID-19 pandemic: a cross-sectional study of medical students and newly qualified doctors in the UK. Ir J Med Sci. 2020:1-2.
11. Norton EJ, Georgiou I, Fung A, Nazari A, Bandyopadhyay S, Saunders KEA. Personal protective equipment and infection prevention and control: a national survey of UK medical students and interim foundation doctors during the COVID-19 pandemic. J Public Health (Oxf). 2021;43(1):67-75.
12. Arora A, Solomou G, Bandyopadhyay S, Simons J, Osborne A, Georgiou I, et al. Adjusting to Disrupted Assessments, Placements and Teaching (ADAPT): a snapshot of the early response by UK medical schools to COVID-19. medRxiv. 2020:2020.07.29.20163907.
13. Kinder F, Harvey A. Covid-19: the medical students responding to the pandemic. BMJ. 2020;369:m2160.
14. Karaca A, Yildirim N, Cangur S, Acikgoz F, Akkus D. Relationship between mental health of nursing students and coping, self-esteem and social support. Nurse Educ Today. 2019;76:44-50.
15. Gillespie CS, Bandyopadhyay S, Jenkins AJ, Hutchinson P. Inspiring the next generation. The Lancet Neurology. 2021;20(4):256-7.
16. Tennant R, Hiller L, Fishwick R, Platt S, Joseph S, Weich S, et al. The Warwick-Edinburgh Mental Well-being Scale (WEMWBS): development and UK validation. Health Qual Life Outcomes. 2007;5:63.
17. Kroenke K, Strine TW, Spitzer RL, Williams JB, Berry JT, Mokdad AH. The PHQ-8 as a measure of current depression in the general population. J Affect Disord. 2009;114(1-3):163-73.
18. Spitzer RL, Kroenke K, Williams JBW, Löwe B. A Brief Measure for Assessing Generalized Anxiety Disorder: The GAD-7. Archives of Internal Medicine. 2006;166(10):1092-7.
19. Löwe B, Decker O, Müller S, Brähler E, Schellberg D, Herzog W, et al. Validation and standardization of the Generalized Anxiety Disorder Screener (GAD-7) in the general population. Med Care. 2008;46(3):266-74.
20. Johnson SU, Ulvenes PG, Øktedalen T, Hoffart A. Psychometric Properties of the General Anxiety Disorder 7-Item (GAD-7) Scale in a Heterogeneous Psychiatric Sample. Front Psychol. 2019;10:1713-.

For peer review only

21. Levis B, Benedetti A, Thombs BD. Accuracy of Patient Health Questionnaire-9 (PHQ-9) for screening to detect major depression: individual participant data meta-analysis. BMJ. 2019;365:l1476.
22. School WM. Collect, Score, Analyse and Interpret WEMWBS 2022 [Available from: https://warwick.ac.uk/fac/sci/med/research/platform/wemwbs/using/howto/.
23. Xie L, Luo H, Li M, Ge W, Xing B, Miao Q. The immediate psychological effects of Coronavirus Disease 2019 on medical and non-medical students in China. Int J Public Health. 2020;65(8):1445-53.
24. Leroy A, Wathelet M, Fovet T, Habran E, Granon B, Martignène N, et al. Mental health among medical, healthcare, and other university students during the first COVID-19 lockdown in France. J Affect Disord Rep. 2021;6:100260-.
25. Lyons Z, Wilcox H, Leung L, Dearsley O. COVID-19 and the mental well-being of Australian medical students: impact, concerns and coping strategies used. Australasian Psychiatry. 2020;28(6):649-52.
26. Spoorthy MS, Pratapa SK, Mahant S. Mental health problems faced by healthcare workers due to the COVID-19 pandemic-A review. Asian J Psychiatr. 2020;51:102119-.
27. Mandal S, Barnett J, Brill SE, Brown JS, Denneny EK, Hare SS, et al. 'Long-COVID': a cross-sectional study of persisting symptoms, biomarker and imaging abnormalities following hospitalisation for COVID-19. Thorax. 2020:thoraxjnl-2020-215818.
28. Xiong J, Lipsitz O, Nasri F, Lui LMW, Gill H, Phan L, et al. Impact of COVID-19 pandemic on mental health in the general population: A systematic review. J Affect Disord. 2020;277:55-64.
29. Lazzerini M, Barbi E, Apicella A, Marchetti F, Cardinale F, Trobia G. Delayed access or provision of care in Italy resulting from fear of COVID-19. Lancet Child Adolesc Health. 2020;4(5):e10-e1.
30. Czeisler M, Marynak K, Clarke KEN, Salah Z, Shakya I, Thierry JM, et al. Delay or Avoidance of Medical Care Because of COVID-19-Related Concerns - United States, June 2020. MMWR Morb Mortal Wkly Rep. 2020;69(36):1250-7.
31. McManus IC, Harborne AC, Horsfall HL, Joseph T, Smith DT, Marshall-Andon T, et al. Exploring UK medical school differences: the MedDifs study of selection, teaching, student and F1 perceptions, postgraduate outcomes and fitness to practise. BMC Med. 2020;18(1):136.
32. Pisaniello MS, Asahina AT, Bacchi S, Wagner M, Perry SW, Wong ML, et al. Effect of medical student debt on mental health, academic performance and specialty choice: a systematic review. BMJ Open. 2019;9(7):e029980.
33. Bandyopadhyay S, Georgiou I, Bligh E, Coyle C, Pancharatnam R, Saunders KEA, et al. SPICE-19: a 3-Month Prospective Cohort Study of 640 Medical Students and Foundation Doctors. Med Sci Educ. 2021;31(5):1621-37.
34. Reverté-Villarroya S, Ortega L, Raigal-Aran L, Sauras-Colón E, Ricomà-Muntané R, Ballester-Ferrando D, et al. Psychological Well-Being in Nursing Students: A Multicentric, Cross-Sectional Study. Int J Environ Res Public Health. 2021;18(6):3020.

For peer review only

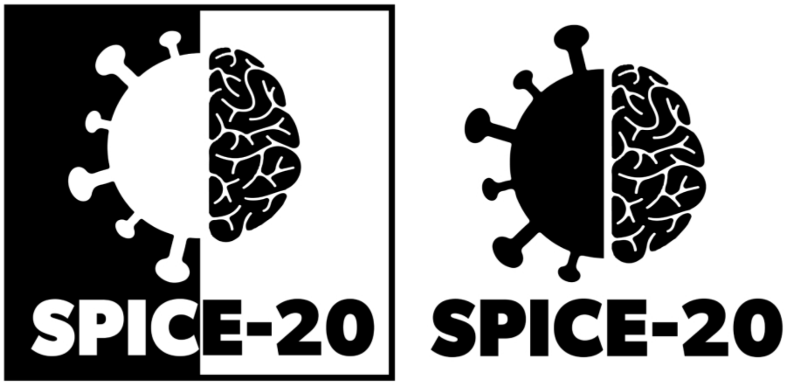

Figure 1. SPICE-20 study logos.

533x755mm (118 x 118 DPI)

Section 1 – Background Information

1. *Are you a medical or nursing student?*
   a. Medical student
      i. Which medical school are you currently enrolled in?
         1. Response from dropdown of medical schools
   b. Nursing Student
      i. Which nursing school are you currently enrolled in?
         1. Response from dropdown of nursing schools
2. How many years (if any) of your degree (excluding intercalation) have you completed so far?
   a. Choose year of study from list
3. Are you currently undertaking an intercalated degree?
   a. Respond yes or no
      i. Have you previously undertaken an intercalated degree?
         1. Respond yes or no
4. How old are you?
   a. Free text box for age
5. What is your gender?
   a. Choose from gender list with free-text box
6. What is your race or ethnicity?
   a. Choose from list of ethnicities with free-text box
7. What is your religion?
   a. Choose from list of religions with free-text box

Section 2 – Wellbeing and Mental Health

WEMWBS

Participants are shown a series of statements and asked to choose an appropriate response from either: 1) None of the time, 2) Rarely, 3) Some of the time, 4) Often and 5) All of the time

1. "I've been feeling optimistic about the future"
2. "I've been feeling useful"
3. "I've been feeling relaxed"
4. "I've been feeling interested in other people"
5. "I've had energy to spare"
6. "I've been dealing with problems well"
7. "I've been thinking clearly"
8. "I've been feeling good about myself"
9. "I've been feeling close to other people"
10. "I've been feeling confident"
11. "I've been able to make up my own mind about things"
12. "I've been feeling loved"
13. "I've been interested in new things"
14. "I've been cheerful"

PHQ-9

Participants are asked, over the last 2 weeks, how often have you been affected by any of the following problems? Responses include: 1) Not at all (0-1 days), 2) Several days (2-6 days), 3) More than half the days (7-11 days), 4) Nearly every day (12-14 days)

1. Little interest or pleasure in doing things?
2. Feeling down, depressed or hopeless?
3. Trouble falling or staying asleep, or sleeping too much?
4. Feeling tired or having little energy?
5. Poor appetite or overeating?
6. Feeling bad about yourself – or that you are a failure or have let yourself or your family down
7. Trouble concentrating on things, such as reading the newspaper or watching television?
8. Moving or speaking so slowly that other people could have noticed. Or the opposite – being so fidgety or restless that you have been moving around a lot more than usual?
9. Thoughts that you would be better off dead or of hurting yourself in some way?

GAD-7

Participants are asked, over the last 2 weeks, how often have you been bothered by the following problems? Responses include: 1) Not at all, 2) Several days, 3) More than half the days, 4) Nearly every day

1. Feeling nervous, anxious or on edge
2. Not being able to stop or control worrying
3. Worrying too much about different things
4. Trouble relaxing
5. Being so restless that it is hard to sit still
6. Becoming easily annoyed or irritable
7. Feeling afraid as if something awful might happen

Other Questions

1. How would you rate your mood before COVID-19 affected the UK (One year ago)? 0 being the worst mood you can imagine and 100 being the best mood you can imagine
   a. Response via sliding scale from 0 to 100
2. How would you rate your mood now? 0 being the worst mood you can imagine and 100 being the best mood you can imagine
   a. Response via sliding scale from 0 to 100

Section 3 – Support

1. Has your university provided any form of mental health and wellbeing support?
   a. Yes
      i. What support has the university provided you with? (please select one or more)
         1. Multiple selection boxes with options and free text box for other
      ii. How confident do you feel accessing the support services available to you?
         1. 5 point Likert scale from not confident at all to very confident
      iii. Have you accessed/used support that your university has provided?
         1. Yes
            a. Has your university support been useful?
               i. Yes
                  1. In what way?
                     a. Free text response
               ii. No
                  1. Why not?
                     a. Free text response
         2. No
      iv. Would you like your university to provide you with more wellbeing support?
         1. Yes (please select one or more)
            a. Multiple selection boxes with options and free text box for other
         2. No
            a. Why not?
               i. Free text response
   b. No

Section 4 – Changes

1. Is your teaching different now compared to before the pandemic (do not answer if in first year)?
   a. Yes
      i. Please select one or more of the following ways teaching/placements have changed:
         1. Multiple selection boxes with options and free text box for other
   b. No
      i. Why not?
         1. Free text response
2. Has there been any positive aspects of the changes to teaching?
   a. Yes
      i. Please detail the positive changes to teaching
         1. Free text response
   b. No
3. What changes could be made to teaching in medical school to improve your wellbeing?
   a. Multiple selection boxes with options and free text box for other
4. Have you worked or are you currently working within a healthcare setting during the course of this pandemic?
   a. Yes
      i. Which of the following roles have you taken up?
         1. Multiple selection boxes with options and free text box for other
      ii. Have you been offered wellbeing support in response to the role you have undertaken?
         1. Yes
            a. Who has provided this support? (please select one or more)
               i. Multiple selection boxes with options and free text box for other
         2. No
   b. No
5. After the pandemic, are you more or less likely to continue in a career in Medicine/Nursing?
   a. 5 point Likert scale from Much less likely to Much more likely
6. Have your future career plans changed since the start of the pandemic?
   a. Yes
      i. If Yes, how come?
         1. Free text response
   b. No

Figure 2: Final SPICE-20 survey design.

913x1291mm (118 x 118 DPI)

Dear Students,

We are a group of student researchers that are interested in investigating the current state of medical student mental health and wellbeing, one year on from the onset of the pandemic. We are inviting all UK medical students, from years 1 to 5 (and intercalating) to complete a short 15 minute survey to help us better understand how you are doing. Whilst completing the survey, you will be asked a variety of questions about your mental health and wellbeing, and the support that you have received. Your IP address will not be recorded and responses will be anonymous. There will not be any follow up survey. Useful links to support services have been provided along with the post-survey debrief. Additional information about the risks versus benefits of taking part in the survey can be found by following the study link below. **Please access the survey by the link below:**

https://psychiatryoxford.qualtrics.com/jfe/form/SV_eQmCH0FLewvbcWO

If you have any questions about the study or your involvement please contact a member of the study team at the following email and we will aim to reply within 24hrs: hlgricha@liverpool.ac.uk

Should you have concerns about your wellbeing do consider contacting you GP or accessing the resources available: <LOCAL SERVICE PROVISION INSERTED HERE>

Many thanks for taking the time to read this,

George Richardson
SPICE-20 Study Team

Figure 3. Standard email template used for all survey dissemination through student email lists, developed by the study team and approved by the research ethics committee.

554x668mm (57 x 57 DPI)

UK N.M.C Approved Adult Nursing Programmes

Approved education institution	Course
**University Of Essex**	Adult Nursing
**University Of Essex**	Adult Nursing (nursing degree apprenticeship route)
**Edinburgh Napier University**	Bachelor of Nursing (Adult)
**University Of Wolverhampton**	Bachelor of Nursing (BNurs (Hons)) Adult Nursing
**University Of Wolverhampton**	Bachelor of Nursing (BNurs (Hons)) Adult Nursing [Apprenticeship]
**University Of Glasgow**	Bachelor of Nursing (Honours)
**University Of Manchester**	Bachelor of Nursing (Hons) (Adult)
**University Of Birmingham**	Bachelor of Nursing (Hons) (Adult)
**University Of Liverpool**	Bachelor of Nursing (Hons) Nursing (Adult)
**University Of Glasgow**	Bachelor of Nursing (ordinary) [fall back award]
**Cardiff University**	Bachelor of Nursing Adult
**University of Derby**	Bachelor of Science (Honours) in Nursing (Adult)
**University of Derby**	Bachelor of Science (Honours) in Nursing (adult) (apprenticeship)
**University Of Sheffield**	BMed Sci (Hons) Nursing (Adult)
**University Of Sheffield**	BMedSci (Hons) Nursing (Adult) Apprenticeship route
**University of Chester**	BN (Hons) Adult Nursing
**University of Chester**	BN (Hons) Adult Nursing
**University of Chester**	BN (Hons) Adult Nursing
**University of Chester**	BN (Hons) Adult Nursing
**University of Chester**	BN (Hons) Adult Nursing

For peer review only

Approved education institution	Course
**University of Chester**	BN (Hons) Adult Nursing Apprenticeship
**University of Chester**	BN (Hons) Adult Nursing Apprenticeship
**University Of Portsmouth**	BN (Hons) Nursing (Adult)
**University of Winchester**	BN (Hons) Nursing (Adult)
**Glyndwr University (formerly North East Wales Inst. of HE)**	BN Nursing
**University Of Edinburgh**	BN nursing (adult)
**University Of Edinburgh**	BN nursing with Honours (adult)
**University of Chester**	BNurs (Hons) Adult
**University of Chester**	BNurs (Hons) Adult (part-time)
**University of Chester**	BNurs (Hons) Adult Apprenticeship
**University Of Wolverhampton**	BNurs (Hons) Adult Nursing
**University Of Wolverhampton**	BNurs (Hons) Adult Nursing Apprenticeship
**University of West London**	BNursing (Hons) Adult
**University Of Hertfordshire**	BSc Hons Nursing (Adult)
**University Of Stirling**	BSc (Honours) Nursing (Adult)
**Coventry University**	BSc (Hons) Adult Nursing
**Coventry University**	BSc (Hons) Adult Nursing (Degree apprenticeship)
**Oxford Brookes University**	BSc (Hons) (Adult)
**London South Bank University**	BSc (Hons) adult nursing
**University of Suffolk**	BSc (Hons) Adult Nursing
**University of West of Scotland**	BSc (Hons) Adult Nursing
**Leeds Beckett University**	BSc (Hons) Adult Nursing
**University of Northampton**	BSc (Hons) Adult Nursing
**Bournemouth University**	BSc (Hons) Adult Nursing

For peer review only

Approved education institution	Course
**University of Roehampton**	BSc (Hons) Adult Nursing
**University Of Dundee**	BSc (Hons) Adult Nursing
**University of West of Scotland**	BSc (Hons) Adult Nursing
**University Of Greenwich**	BSc (Hons) Adult Nursing
**University of Bedfordshire**	BSc (Hons) Adult Nursing
**Bournemouth University**	BSc (Hons) Adult Nursing (Apprenticeship)
**University of Bedfordshire**	BSc (Hons) Adult Nursing (apprenticeship)
**University of Sunderland**	BSc (Hons) Adult Nursing (blended)
**University of Sunderland**	BSc (Hons) Adult Nursing (blended)
**Leeds Beckett University**	BSc (Hons) Adult Nursing (degree apprenticeship)
**Manchester Metropolitan University**	BSc (Hons) Adult Nursing (Pre-registration)
**University of Suffolk**	BSc (Hons) Adult Nursing [degree apprenticeship]
**Coventry University**	BSc (Hons) Adult Nursing Blended Learning
**Coventry University**	BSc (Hons) Adult Nursing Blended Learning Nursing Degree Apprenticeship
**University Of Greenwich**	BSc (Hons) Adult Nursing Degree Apprenticeship
**Solent University**	BSc (Hons) Adult Nursing Practice – apprenticeship
**University of Sunderland**	BSc (Hons) adult nursing practice
**Solent University**	BSc (Hons) Adult Nursing Practice
**University Of Huddersfield**	BSc (Hons) Degree Apprenticeship - Registered Nurse Child (Blended Learning)
**University Of Huddersfield**	BSc (Hons) Degree Apprenticeship-Registered Nurse: Adult

Approved education institution	Course
**BPP**	BSc (Hons) Nursing (Adult) [Nursing Degree Apprenticeship]
**BPP**	BSc (Hons) Nursing (Adult) [pre registration nursing]
**University of East Anglia**	BSc (Hons) nursing - Adult
**University Of Huddersfield**	BSc (Hons) Nursing - Adult
**University Of The West Of England, Bristol**	BSc (Hons) Nursing - Adult
**University of East Anglia**	BSc (Hons) Nursing - Adult Degree Apprenticeship
**Queen Margaret University**	BSc (Hons) Nursing
**University Of Plymouth**	BSc (Hons) Nursing (Adult Health)
**University Of Plymouth**	BSc (Hons) Nursing (Adult Health) - Nurse Degree Apprenticeship
**University Of York**	BSc (hons) Nursing (Adult)
**University of Salford**	BSc (Hons) Nursing (adult)
**University of Bolton**	BSc (Hons) Nursing (adult)
**University of Nottingham**	BSc (Hons) Nursing (adult)
**Buckinghamshire New University**	BSc (Hons) Nursing (Adult)
**Anglia Ruskin University**	BSc (Hons) Nursing (Adult)
**Swansea University**	BSc (Hons) Nursing (Adult)
**Edge Hill University**	BSc (Hons) Nursing (Adult)
**Edge Hill University**	BSc (Hons) Nursing (Adult)
**The Open University**	BSc (Hons) Nursing (Adult)
**The University of Hull**	BSc (Hons) Nursing (Adult)
**The University of Hull**	BSc (Hons) Nursing (Adult)
**University of Ulster**	BSc (Hons) Nursing (Adult)

Approved education institution	Course
**University Of East London**	BSc (Hons) Nursing (Adult)
**University Of Leeds**	BSc (Hons) Nursing (Adult)
**University College Birmingham**	BSc (Hons) Nursing (Adult)
**University Of Southampton**	BSc (Hons) Nursing (Adult)
**Canterbury Christ Church University**	BSc (Hons) Nursing (Adult)
**Birmingham City University**	BSc (Hons) Nursing (Adult)
**Anglia Ruskin University**	BSc (Hons) Nursing (Adult)
**Swansea University**	BSc (Hons) Nursing (Adult)
**The Robert Gordon University**	BSc (Hons) Nursing (Adult)
**University of Bradford**	BSc (Hons) Nursing (Adult)
**The Open University**	BSc (Hons) Nursing (Adult)
**The Open University**	BSc (Hons) Nursing (Adult)
**University of Gloucestershire**	BSc (Hons) Nursing (Adult)
**University Of Hertfordshire**	BSc (Hons) Nursing (adult) - Nursing Degree Apprenticeship
**The Open University**	BSc (Hons) Nursing (Adult) - Nursing Degree Apprenticeship
**Birmingham City University**	BSc (Hons) Nursing (Adult) - Nursing Degree Apprenticeship
**University of Gloucestershire**	BSc (Hons) Nursing (Adult) (Apprenticeship)
**University of Gloucestershire**	BSc (Hons) Nursing (Adult) (Blended Learning)
**Edge Hill University**	BSc (Hons) Nursing (Adult) (fall back award)
**University Of Leeds**	BSc (Hons) Nursing (Adult) (International)
**Canterbury Christ Church University**	BSc (Hons) Nursing (Adult) (NDA)
**University of Bolton**	BSc (Hons) Nursing (Adult) (Part-time)

Approved education institution	Course
**University of Bolton**	BSc (Hons) Nursing (Adult) (Registered Nursing Higher Apprenticeship) (Part-time)
**University of Bradford**	BSc (Hons) Nursing (Adult) [fall back award]
**Buckinghamshire New University**	BSc (Hons) Nursing (Adult) Apprenticeship
**University of Salford**	BSc (Hons) Nursing (adult) Nurse Degree Apprentice
**University of Bolton**	BSc (Hons) Nursing (adult) nurse degree apprenticeship (NDA) route
**Middlesex University**	BSc (Hons) Nursing (adult) seconded pathway
**De Montfort University**	BSc (Hons) Nursing (Adult) with NMC Registration (Apprenticeship route)
**University Of East London**	BSc (Hons) Nursing (Adult)-Registered Nurse (Degree) Apprenticeship Route
**Queen Margaret University**	BSc (Hons) Nursing (fall back award)
**University of Lincoln**	BSc (Hons) Nursing (Registered Nurse - Adult)
**University of South Wales**	BSc (Hons) Nursing Adult
**Nottingham Trent University**	BSc (Hons) Nursing Adult
**University Of Brighton**	BSc (Hons) Nursing Adult
**University of Worcester**	BSc (Hons) Nursing Adult
**Sheffield Hallam University**	BSc (Hons) Nursing Adult (Part Time)
**University Of The West Of England, Bristol**	BSc (Hons) Nursing apprenticeship - Adult
**University Of Huddersfield**	BSc (Hons) Nursing Child (Blended Learning)
**University of Sunderland**	BSc (Hons) nursing degree apprenticeship (adult)
**Nottingham Trent University**	BSc (Hons) Nursing Degree Apprenticeship Adult
**Swansea University**	BSc (Hons) Nursing part-time (Adult)

For peer review only - http://bmjopen.bmj.com/site/about/guidelines.xhtml

Approved education institution	Course
**University Of Northumbria At Newcastle**	BSc (Hons) nursing science (adult)
**University Of Northumbria At Newcastle**	BSc (Hons) nursing science apprenticeship (adult)
**Teesside University**	BSc (Hons) Nursing Studies (Adult) (Pre-registration) Apprenticeship route
**Glasgow Caledonian University**	BSc (Hons) Nursing Studies: Adult Nursing
**Liverpool John Moores University**	BSc (Hons) Nursing with Registered Nurse Status (Adult)
**Liverpool John Moores University**	BSc (Hons) Nursing with Registered Nurse Status (Adult)
**University of Cumbria**	BSc (Hons) Nursing/RN:Adult Nursing
**University of Cumbria**	BSc (Hons) Nursing/RN:Adult Nursing (Apprenticeship)
**Keele University**	BSc (Hons) Nursing: Adult
**Keele University**	BSc (Hons) Nursing: Adult (NDA)
**Kingston University & St George's University of London**	BSc Adult Nursing
**University Of Dundee**	BSc Adult Nursing
**University of West of Scotland**	BSc Adult Nursing
**University Of Greenwich**	BSc Adult Nursing (Degree Apprenticeship) (fall back award)
**University Of Greenwich**	BSc Adult Nursing (fall back award)
**Middlesex University**	BSc Hons Nursing Adult
**University of Highlands and Islands**	BSc Nursing
**City, University of London**	BSc Nursing (Adult)
**Oxford Brookes University**	BSc Nursing (Adult)
**The Robert Gordon University**	BSc Nursing (Adult)

For peer review only

Approved education institution	Course
**The Robert Gordon University**	BSc Nursing (Adult)
**University Of Stirling**	BSc Nursing (Adult)
**The Robert Gordon University**	BSc Nursing (Adult) (fall back award)
**Glasgow Caledonian University**	BSc Nursing Studies: Adult Nursing
**King's College London**	BSc Nursing with Registration as an Adult Nurse
**University of Ulster**	BSc(Hons) Nursing (Adult)
**University of Lincoln**	Degree Science Nursing (Registered Nurse - Adult: Apprenticeship Route)
**University of Salford**	MA Nursing (adult)
**University Of Wolverhampton**	Master of Adult Nursing
**University Of Wolverhampton**	Master of Nursing (Adult Nursing)
**University Of Birmingham**	Master of Nursing (Adult)
**University Of Wolverhampton**	Master of Nursing (MNurs) Adult Nursing
**University Of Birmingham**	Master of Science in Nursing (Adult)
**University of Derby**	Master of Science in Nursing (Adult)
**University of Derby**	Master of Science in Nursing (adult) (apprenticeship)
**University Of Manchester**	Masters in Nursing (Adult)
**University Of Edinburgh**	Masters in Nursing with pre-registration (adult)
**University Of Sheffield**	MMedSci Nursing (Adult)
**Queen Margaret University**	MN Nursing
**Edge Hill University**	MNSW Adult Nursing and Social Work
**University of Sunderland**	MNurse (Adult)
**University Of York**	MNursing (Adult)
**University Of Hertfordshire**	MSc Hons Nursing (Adult)

For peer review only - http://bmjopen.bmj.com/site/about/guidelines.xhtml

Approved education institution	Course
**University Of Northumbria At Newcastle**	MSc (Hons) nursing science (adult)
**University Of Dundee**	MSc Adult Nursing
**University Of Greenwich**	MSc Adult Nursing
**University of Chester**	MSc Adult Nursing
**University of Chester**	MSc Adult Nursing
**University of Chester**	MSc Adult Nursing
**University of Bedfordshire**	MSc Adult Nursing
**London South Bank University**	MSc adult nursing
**University of West of Scotland**	MSc Adult Nursing
**University of Chester**	MSc Adult Nursing
**Bournemouth University**	MSc Adult Nursing
**Kingston University & St George's University of London**	MSc Adult Nursing
**Coventry University**	MSc Adult Nursing
**University of Bedfordshire**	MSc Adult Nursing (apprenticeship)
**Birmingham City University**	MSc Adult Nursing (pre-registration)
**Manchester Metropolitan University**	MSc Adult Nursing (Pre-registration)
**University of Chester**	MSc Adult Nursing Apprenticeship
**Coventry University**	MSc Adult Nursing Blended Learning (pre-registration)
**Coventry University**	MSc Adult Nursing Blended Learning (pre-registration) Nursing Degree Apprenticeship
**University Of Southampton**	MSc Nursing (Adult)
**Canterbury Christ Church University**	MSc Nursing (Adult)
**City, University of London**	MSc Nursing (Adult)

Approved education institution	Course
**University Of Hertfordshire**	MSc Nursing (adult) - Nursing Degree Apprenticeship (part time route)
**University of Nottingham**	MSc nursing (graduate entry) (adult)
**University of Lincoln**	MSc Nursing (Pre-Registration – Adult)
**Edge Hill University**	MSc Nursing (Pre-registration Adult)
**University Of Huddersfield**	MSc Nursing (pre-registration) - Adult
**Swansea University**	MSc Nursing Pre-registration (Adult)
**Teesside University**	MSc Nursing Studies (Adult Apprenticeship)
**Glasgow Caledonian University**	MSc Nursing Studies: Adult Nursing
**University of Central Lancashire**	MSc nursing with registered nurse (adult)
**Liverpool John Moores University**	MSc Nursing with Registered Nurse Status (Adult)
**Liverpool John Moores University**	MSc Nursing with Registered Nurse Status (Adult)
**King's College London**	MSc Nursing with Registration as an Adult Nurse
**University Of Plymouth**	MSc Pre-registration Nursing (Adult Health)
**University Of Huddersfield**	MSc Pre-registration Nursing apprenticeship-Adult
**University Of Huddersfield**	MSc Pre-registration Nursing Apprenticeship Child (Blended Learning)
**University Of Huddersfield**	MSc Pre-registration Nursing Child (Blended Learning
**University of Cumbria**	MSc Pre-Registration Nursing/Registered Nurse: Adult Nursing
**Edge Hill University**	MSci Nurse Paramedic (Adult)
**University Of Exeter**	MSci nursing (adult)
**University of Leicester**	MSci Nursing with Leadership Adult Nursing

For peer review only - http://bmjopen.bmj.com/site/about/guidelines.xhtml

Approved education institution	Course
**Middlesex University**	Nurse Degree Apprenticeship Adult (4 year direct entry route)
**Middlesex University**	Nurse Degree Apprenticeship Adult (4 year part-time employed route)
**University of West London**	Nurse Degree Apprenticeship BNursing (Hons) Adult
**University of Central Lancashire**	Nursing Degree Apprenticeship route – Adult
**Teesside University**	Nursing Studies (Adult) (Pre-registration) BSc (Hons)
**Teesside University**	Nursing Studies (Adult) (Pre-Registration) MSc
**University of West of Scotland**	PG Dip Adult Nursing
**London South Bank University**	PG Dip adult nursing
**University Of Greenwich**	PG Dip Adult Nursing (fall back award)
**University of South Wales**	PG Dip Nursing Adult
**University of West London**	PG Diploma Nursing Adult
**Edge Hill University**	PgDip Nursing (second registration Adult)
**University Of Southampton**	Postgraduate Diploma Nursing (Adult)
**The Queen's University Of Belfast**	Pre-registration nursing - Adult
**Sheffield Hallam University**	Pre-registration nursing - Adult
**The Queen's University Of Belfast**	Pre-registration nursing - Adult (Graduate Entry)
**Staffordshire University**	Registered Nurse (Adult)
**University Of Brighton**	Registered Nurse Degree Apprenticeship (Adult)

For peer review only - http://bmjopen.bmj.com/site/about/guidelines.xhtml

UK M.S.C Approved Medicine and Surgery Programmes

Institution Name
University of Aberdeen School of Medicine and Dentistry
Anglia Ruskin University School of Medicine
Aston University Medical School
Barts and The London School of Medicine and Dentistry
University of Birmingham College of Medical and Dental Sciences
Brighton and Sussex Medical School
University of Bristol Medical School
University of Buckingham Medical School
University of Cambridge School of Clinical Medicine
Cardiff University School of Medicine
University of Dundee School of Medicine
Edge Hill University Medical School
The University of Edinburgh Medical School
University of Exeter Medical School
University of Glasgow School of Medicine
Hull York Medical School
Imperial College London Faculty of Medicine
Keele University School of Medicine
Kent and Medway Medical School
King's College London GKT School of Medical Education
Lancaster University Medical School
University of Leeds School of Medicine
University of Leicester Medical School
University of Liverpool School of Medicine
University of Manchester Medical School
Newcastle University School of Medical Education
Norwich Medical School
University of Nottingham School of Medicine

For peer review only

University of Nottingham - Lincoln Medical School
University of Oxford Medical Sciences Division
Plymouth University Peninsula Schools of Medicine and Dentistry
Queen's University Belfast School of Medicine
University of Sheffield Medical School
University of Southampton School of Medicine
University of St Andrews School of Medicine
St George's, University of London
University of Sunderland School of Medicine
Swansea University Medical School
University of Central Lancashire School of Medicine
University College London Medical School
University of Warwick Medical School

For peer review only

Patient Information Section

General information

The aim of this study is to determine the impact of the COVID-19 pandemic on the mental health of UK medical and nursing students, one year down the line from the initial outbreak. We are inviting all medical and nursing students enrolled at university in the UK to participate in this study. Please read the following section carefully before taking part. If you have any questions regarding the study please contact the research team via the following email: kate.saunders@psych.ox.ac.uk

For peer review only

What is the purpose of the study?

The purpose of SPICE-20 is to evaluate the continued impact of the COVID-19 pandemic on UK medical students, one year on from the SPICE-19 study. In addition, we aim to determine the effect on UK nursing students. In doing so we hope to provide feedback to UK medical schools and Nursing institutions about the current collective mental health of student healthcare professionals. We believe this information is vital to ensure adequate mental wellbeing service provision is in place at higher education institutions.

Why am I being invited to participate in this study?

You have been invited to take part in this study because we are recruiting medical and nursing students to participate and feedback on their continued mental wellbeing during the UK pandemic. You are eligible to participate if you are a current UK medical or nursing student in the UK and have access to the internet.

What happens if I choose to participate?

Participation in this study is entirely voluntary. To be involved you will be required to complete a short survey online. The survey will ask for general background details about you including: your current educational status, age, gender, ethnicity. Additionally, questions will ask about your current mood and mental wellbeing. Your decision to participate in this study will not have any academic impact and will be entirely anonymous. The survey is expected to take approximately 10-15 minutes to complete however it may be done as quick or slow as you like. There is no prior knowledge required to participate. We will ask for your consent for collection and storage of data in accordance with General

Data Protection Regulation (GDPR) before the survey begins. More information regarding the rules and regulations of GDPR can be found at www.gdpr-info.eu .

Do I have to participate?

As stated above, participation in the SPICE-20 study is entirely voluntary and anonymous. Consent for involvement may be withdrawn anytime prior to completion of the survey. To do so, simply close the tab or browser containing the survey. Following this, no personal data provided up to that point in the survey will be stored or used for research or any other purpose. Completion of all questions is entirely optional. This means that participation is still possible even if there are certain questions you would prefer not to answer. If there is a question you would like to omit from your responses, simply select the option "Prefer not to answer". Any data collected will not be provided to your higher education institution without being anonymised, meaning your feedback will not make your identifiable.

Are there any risks of being involved?

Some of the questions included in the questionnaire may potentially cause some distress by brining up painful memories. If at any point during completion of the survey you are feeling distressed you may stop or choose not to answer troubling questions. Included with the study information is mental wellbeing support services that are available to access.

Are there any benefits to being involved?

The completion of this survey will not provide any academic benefit at your respective institutions. However, the results of SPICE-20 will be used to inform university wellbeing service provision. This means that by participating in the study you have the opportunity to provide anonymous feedback to your institution about what you feel is being done well, or could be done better to support students. Additionally, this survey may provide be useful as a personal opportunity for reflection.

How will my data be used?

Your answers will be completely anonymous and no identifiable data will be included. Data will be stored in a password protected file and will only be accessible by individuals on the steering/writing committee that have a reason to access it. IP addresses will not be stored. If you provide your email address, it will be deleted at the end of the study and not linked to any information you provide.

For peer review only

Anonymised responses to the survey will be stored for a minimum of ten years after publication or public release.

Who will have access to your data?

Qualtrics is the data platform used for SPICE-20. They are the data controller in respect to the personal data they hold and as such they determine how personal data is used. Their privacy notice can be found at: www.qualtrics.com/privacy-statement. Qualtrics will share any email address you provide with the University of Oxford for the purposes of research only. Responsible members of the University of Oxford may be given access to your data for monitoring or audit of the study and ensuring that guidelines are complied with.

Where will the results be published?

The results of SPICE-20 may be published in a peer reviewed academic journal. In addition, the results of the study may be presented at conferences, either in oral or poster format. The study is targeting a completion date of September 2021. Following this provisional date a summary of the results and outcomes will be available on request to the study team.

Who do I contact if I have any queries or wish to make a complaint about the study?

Our study team aims to operate in a transparent manner. If you have any queries or complaints regarding the study aims, distribution, or any other aspect please contact the study team at kate.saunders@psych.ox.ac.uk and we will do our best to provide an adequate response. Our research team aims to reply within 10 working days. If you have any further issues or wish to escalate your inquiry please contact the Chair of the Medical Sciences Inter-Divisional Research Ethics Committee. Email: ethics@medsci.ox.ac.uk. Address: Research Services, University of Oxford, Wellington Square, Oxford, OX1 2JD.

Who do I contact for further details?

For any queries or other details, contact the principle investigator Professor Kate Saunders at kate.saunders@psych.ox.ac.uk.

For peer review only

Please check the box to confirm you are the age of 18 years old or older

☐ I certify that I am 18 years or older.

If you have read the information above and agree to participate with the understanding that the data (including personal data) you submit will be processed accordingly, please check the relevant box to get started

☐ Yes, I agree to take part.

For peer review only

STROBE Statement—checklist of items that should be included in reports of observational studies

	Item No.	Recommendation	Page No.	Relevant text from manuscript
**Title and abstract**	1	(*a*) Indicate the study's design with a commonly used term in the title or the abstract	P1, Title	
		(*b*) Provide in the abstract an informative and balanced summary of what was done and what was found	P2, Abstract	
**Introduction**				
Background/rationale	2	Explain the scientific background and rationale for the investigation being reported	P3	
Objectives	3	State specific objectives, including any prespecified hypotheses	P4, Last Paragraph	
**Methods**				
Study design	4	Present key elements of study design early in the paper	From P4	
Setting	5	Describe the setting, locations, and relevant dates, including periods of recruitment, exposure, follow-up, and data collection	From P4 To P8	
Participants	6	(*a*) *Cohort study*—Give the eligibility criteria, and the sources and methods of selection of participants. Describe methods of follow-up	N/A	
		Case-control study—Give the eligibility criteria, and the sources and methods of case ascertainment and control selection. Give the rationale for the choice of cases and controls	N/A	
		Cross-sectional study—Give the eligibility criteria, and the sources and methods of selection of participants	P6	
		(*b*) *Cohort study*—For matched studies, give matching criteria and number of exposed and unexposed	N/A	
		Case-control study—For matched studies, give matching criteria and the number of controls per case	N/A	
Variables	7	Clearly define all outcomes, exposures, predictors, potential confounders, and effect modifiers. Give diagnostic criteria, if applicable	P5	
Data sources/ measurement	8*	For each variable of interest, give sources of data and details of methods of assessment (measurement). Describe comparability of assessment methods if there is more than one group	P5	
Bias	9	Describe any efforts to address potential sources of bias	N/A	
Study size	10	Explain how the study size was arrived at	P7	

Continued on next page

For peer review only - http://bmjopen.bmj.com/site/about/guidelines.xhtml

Quantitative variables	11	Explain how quantitative variables were handled in the analyses. If applicable, describe which groupings were chosen and why	P7
Statistical methods	12	(*a*) Describe all statistical methods, including those used to control for confounding	P7
		(*b*) Describe any methods used to examine subgroups and interactions	N/A
		(*c*) Explain how missing data were addressed	N/A
		(*d*) *Cohort study*—If applicable, explain how loss to follow-up was addressed	N/A
		Case-control study—If applicable, explain how matching of cases and controls was addressed	N/A
		Cross-sectional study—If applicable, describe analytical methods taking account of sampling strategy	P5
		(*e*) Describe any sensitivity analyses	N/A
**Results**			
Participants	13*	(a) Report numbers of individuals at each stage of study—eg numbers potentially eligible, examined for eligibility, confirmed eligible, included in the study, completing follow-up, and analysed	N/A
		(b) Give reasons for non-participation at each stage	N/A
		(c) Consider use of a flow diagram	N/A
Descriptive data	14*	(a) Give characteristics of study participants (eg demographic, clinical, social) and information on exposures and potential confounders	N/A
		(b) Indicate number of participants with missing data for each variable of interest	N/A
		(c) *Cohort study*—Summarise follow-up time (eg, average and total amount)	N/A
Outcome data	15*	*Cohort study*—Report numbers of outcome events or summary measures over time	N/A
		Case-control study—Report numbers in each exposure category, or summary measures of exposure	N/A
		Cross-sectional study—Report numbers of outcome events or summary measures	N/A
Main results	16	(*a*) Give unadjusted estimates and, if applicable, confounder-adjusted estimates and their precision (eg, 95% confidence interval). Make clear which confounders were adjusted for and why they were included	N/A
		(*b*) Report category boundaries when continuous variables were categorized	N/A
		(*c*) If relevant, consider translating estimates of relative risk into absolute risk for a meaningful time period	N/A

Continued on next page

For peer review only

Other analyses	17	Report other analyses done—eg analyses of subgroups and interactions, and sensitivity analyses	N/A
**Discussion**			
Key results	18	Summarise key results with reference to study objectives	N/A
Limitations	19	Discuss limitations of the study, taking into account sources of potential bias or imprecision. Discuss both direction and magnitude of any potential bias	From P9 To P10
Interpretation	20	Give a cautious overall interpretation of results considering objectives, limitations, multiplicity of analyses, results from similar studies, and other relevant evidence	N/A
Generalisability	21	Discuss the generalisability (external validity) of the study results	N/A
**Other information**			
Funding	22	Give the source of funding and the role of the funders for the present study and, if applicable, for the original study on which the present article is based	P11

*Give information separately for cases and controls in case-control studies and, if applicable, for exposed and unexposed groups in cohort and cross-sectional studies.

Note: An Explanation and Elaboration article discusses each checklist item and gives methodological background and published examples of transparent reporting. The STROBE checklist is best used in conjunction with this article (freely available on the Web sites of PLoS Medicine at http://www.plosmedicine.org/, Annals of Internal Medicine at http://www.annals.org/, and Epidemiology at http://www.epidem.com/). Information on the STROBE Initiative is available at www.strobe-statement.org.

For peer review only

For peer review only - http://bmjopen.bmj.com/site/about/guidelines.xhtml
